# Internet-based and mobile-based cognitive behavioral therapy for chronic diseases: a systematic review and meta-analysis

**DOI:** 10.1038/s41746-023-00809-8

**Published:** 2023-04-28

**Authors:** Tiffany Junchen Tao, Teck Kuan Lim, Ernest Tsun Fung Yeung, Huinan Liu, Phoenix Bibha Shris, Lawrence Ka Yin Ma, Tatia Mei Chun Lee, Wai Kai Hou

**Affiliations:** 1 Centre for Psychosocial Health, The Education University of Hong Kong, Hong Kong SAR, China; 2 Department of Special Education and Counselling, The Education University of Hong Kong, Hong Kong SAR, China; 3 Department of Psychology, The Education University of Hong Kong, Hong Kong SAR, China; 4 State Key Laboratory of Brain and Cognitive Sciences, The University of Hong Kong, Hong Kong SAR, China; 5 Laboratory of Neuropsychology & Human Neuroscience, The University of Hong Kong, Hong Kong SAR, China

**Keywords:** Outcomes research, Diseases, Health care

## Abstract

Positive adjustment to chronic diseases reduces psychiatric comorbidity and enhances quality of life. Very little is known about the benefit of internet-based and mobile-based Cognitive Behavioral Therapy (IM-CBT) on physical outcomes and its reciprocal interactions with psychiatric outcomes, the active therapeutic elements, and effect moderators among people with major chronic medical conditions. In this systematic review and meta-analysis (PROSPERO: CRD42022265738), CINAHL of Systematic Reviews, MEDLINE, PsycINFO, PubMed, Web of Science are systematically searched up to 1 June 2022, for randomized controlled trials (RCTs) comparing IM-CBT against non-CBT control condition(s) among people with chronic disease(s). Primary outcomes include improvements in psychiatric symptoms (depressive, anxiety, PTSD symptoms, general psychological distress) from baseline to post-intervention and follow-ups. Secondary outcomes include improvements in physical distress (physical symptoms, functional impairment, self-rated ill health, objective physiological dysfunction). Among 44 RCTs (5077 patients with seven different chronic diseases), IM-CBT improves depressive symptoms, anxiety symptoms, and general psychological distress at post-intervention and across follow-ups, and improves physical distress and functional impairment at post-intervention. Preliminary evidence suggests that behavioral modification and problem-solving could be necessary components to reduce psychiatric symptoms in IM-CBT, whereas cognitive restructuring, psychoeducation, and mindfulness elements relate to reduced physical distress. IM-CBT shows stronger benefits in chronic pain, cancer, arthritis, and cardiovascular disease, relative to other conditions. Changes in psychiatric symptoms and physical distress prospectively predict each other over time. IM-CBT is an effective intervention for comprehensive symptom management among people with chronic diseases.

## Introduction

Chronic diseases are responsible for not only deaths but also years lived with disability, a common expansion of morbidity^1^. Growing numbers of people live with chronic ill health and compromised quality of life over the past decades^1^, among which one-third experience multiple conditions^2^. Interventions for mental health are also prioritized to be integrated into the management of chronic medical conditions^3–6^. Those patients are 2-3 times more likely to have comorbid mental ill health such as depressive/anxiety disorders relative to the general population^3,7^. Comorbid physical and psychiatric conditions could jointly predict poorer prognosis^3,5^ and add financial and psychosocial burden^3,6,8^. With the ever-increasing burden on the healthcare system, digitalizing the management of chronic conditions^9–11^ could overcome practical barriers such as immune compromise, mobility difficulties, shortage of clinical personnel, and health disparity^12,13^.

The clinical benefits of specialized psychological treatment namely Cognitive Behavioral Therapy (CBT) delivered across the internet and/or mobile devices [Internet-based and mobile-based CBT (IM-CBT)] for people with chronic diseases should be rigorously reviewed. IM-CBT has been shown to be as effective as face-to-face CBT^14,15^ and increase the accessibility of care for underserved patients^16,17^. Two meta-analyses of different chronic diseases^18,19^ and one systematic review of people with rheumatic conditions^20^ have documented the effectiveness of internet-based CBT in reducing psychiatric and/or physical symptoms. However, previous work did not comprehensively study how IM-CBT effects might differ across various diagnostic conditions and/or health outcomes. More importantly, very little is known about the therapeutic elements specifically responsible for the improved clinical outcomes, the effect moderators, and the reciprocity between mental health outcomes and secondary physical health outcomes.

This systematic review and meta-analysis aims to examine the effectiveness of IM-CBT in reducing psychiatric symptoms among patients across most common chronic medical conditions in randomized controlled trials. In-depth analyses were also conducted on active CBT treatment components, the influence of patient-related/treatment-related factors, and the prospective association between resulting psychiatric symptoms and physical distress.

IM-CBT relates to reduced psychiatric symptoms and physical distress, with the two improvements prospectively predicting each other. Among the CBT components, behavioral modification and problem-solving reduce psychiatric symptoms whereas cognitive restructuring, psychoeducation, and mindfulness reduce physical distress. IM-CBT benefits patients with chronic pain, cancer, arthritis, and cardiovascular disease more, psychologically and physically, relative to those with other diseases. Our results attest the clinical utility of IM-CBT for patients with chronic diseases.

## Results

**Fig. 1:**
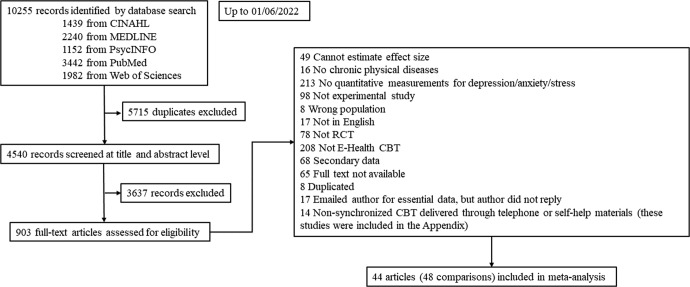
PRISMA flowchart.

The study selection process is shown in Fig. 1. This study included 44 eligible RCTs^21–64^ reporting 48 IM-CBT-to-control comparisons among a total of 5077 patients (2728 in intervention, 2349 in control groups). Descriptive information on included studies is summarized in Table 1 and Supplementary Tables 1–5.

**Table 1:** Descriptive statistics of 44 included studies.

Characteristics	Studies with characteristics, No. (%)
Country	
North America	12 (27.27)
Europe	20 (45.45)
Australia	10 (22.73)
Asia	2 (4.55)
Sample size, *mean* (*SD*) [range]	
Total	115.39 (122.48) [15–562]
Intervention group	59.30 (63.07) [7–280]
Control group	51.07 (54.26) [8–282]
Risk of bias	
High risk	17 (38.64)
Some concerns	24 (54.55)
Low risk	3 (6.82)
Attrition rate at post-intervention	
High (>20%)	7 (15.91)
Moderate (5–20%)	32 (72.73)
Low (<5%)	5 (11.36)
Control group type	
Active control	21 (47.73)
Non-active control	24 (54.55)
Inclusion of follow-up data	
First follow-up data reported	19 (43.18)
Last follow-up data reported	6 (13.64)
Follow-up duration after intervention (weeks)	
First follow-up, *mean* (*SD*) [range]	15.78 (7.31) [8–36]
Last follow-up, *mean* (*SD*) [range]	26 (12.81) [12–48]
Psychiatric symptoms	
Depressive symptoms	39 (88.64)
Anxiety symptoms	30 (68.18)
Depressive and anxiety symptoms	6 (13.64)
Post-traumatic stress disorder (PTSD) symptoms	3 (6.82)
General psychological distress	10 (22.73)
Physical distress	
Physical symptoms	27 (61.36)
Functional impairment	19 (43.18)
Self-rated ill health	4 (9.09)
Objective physiological dysfunction	2 (4.55)
Proportion of female, *mean* (*SD*) [range]	71.11% (18.97%) [28.81%–100.00%]
Age of all included patients, *mean* (*SD*) [range]	47.61 (13.27) [11–91]
Chronic diseases	
Chronic pain	19 (43.18)
Cancer	7 (15.91)
Arthritis	6 (13.64)
Cardiovascular disease	4 (9.09)
Diabetes	2 (4.55)
HIV	1 (2.27)
Multiple sclerosis	1 (2.27)
Different chronic diseases	4 (9.09)
Physical or psychiatric comorbidity	
Yes	23 (52.27)
No	21 (47.73)
Medication received for physical condition(s)	
Yes	22 (50.00)
No	22 (50.00)
Surgery received for physical condition(s)	
Yes	6 (13.64)
No	38 (86.36)
Supplement and/or other received for physical condition(s)	
Yes	5 (11.36)
No	39 (88.64)
Medication received for psychiatric condition(s)	
Yes	12 (27.27)
No	32 (72.73)
Psychotherapy received for psychiatric condition(s)	
Yes	3 (6.82)
No	41 (93.18)
Intervention delivery platform	
Videoconference	3 (6.82)
Web-based	39 (88.64)
Mobile app	3 (6.82)
Guidance	
Guided	32 (72.73)
Unguided	14 (31.82)
Intervention duration (no. of sessions)	
Short (<12 sessions) *n* (%) [range]	35 (79.55) [4–10]
Medium/long (≥12 sessions) *n* (%) [range]	9 (20.45) [12–48]
Therapeutic elements	
Behavioral modification	43 (97.73)
Cognitive restructuring	30 (68.18)
Problem-solving	43 (97.73)
Psychoeducation	37 (84.09)
Mindfulness	28 (63.64)
Intention-to-treat analysis	
Yes	33 (75.00)
No	11 (25.00)

The detailed information of individual studies is available in Supplementary Tables 1–5. Only 1 article did not include behavioral modification^30^. Only 1 article did not include problem-solving^51^.

Definitions. “Attrition rate at post-intervention” was defined as: <5% = low, 5–20% = moderate, and >20% = high^106^. “Non-active” control group included waitlist control (WLC) and treatment-as-usual (TAU) / standard care (SC); “Active” control group included information/education (*k* = 10), discussion forum (*k* = 5), relaxation (*k* = 2), attention control (scheduled contact) (*k* = 2), supportive therapy (*k* = 1), computerized cognitive remediation therapy (*k* = 1), and lifestyle management (*k* = 1). (1 article contained both active and non-active control groups;^42^ 1 article contained two active control groups^59^.) “General psychological distress” included distress (e.g., Kessler 10-item Psychological Distress Scale [K-10]) and stress (e.g., “Depression Anxiety Stress Scale-21 (DASS-21) Stress Subscale”).

Age range was compiled based on retrievable information from *n* = 24 (54.55%) studies (the remaining *n* = 20 studies did not provide such information). For “Intervention delivery format”, 1 article offered intervention through a mix of web-based (main) and mobile-app (complementary) platforms^40^. “Guidance” was defined as: “Guided” refers to therapists’ therapeutic input, including active provision of intervention, feedback, and/or support; “Unguided” refers to technical/adherence or other non-specified assistance only^12^. (2 articles contained both guided and unguided interventions^21,33^.) “Intervention duration” was defined as: <12 sessions = short, 12–16 sessions = medium, >16 sessions = long^19^.

### Included studies

Twelve studies were conducted in North America (US, Canada)^22,29,34,36,38,51,52,54,56,60,63,64^, 20 in Europe (Netherlands, Sweden, UK, Ireland, Germany, Norway)^21,24–27,31,35,37,39,40,43–45,47,53,55,58,59,61,62^, 10 in Australia^23,28,30,32,33,41,46,48–50^, and 2 in Asia (Japan, Korea)^42,57^. Three (6.82%), 24 (54.55%), and 17 (38.64%) studies were assessed to have low, some, and high risks of overall bias, respectively (Supplementary Table 6).

Included patients had a mean age of 47.61 (*SD* = 13.27) years (range = 11–91 years, based on retrievable information in *n* = 24 studies) (Supplementary Table 1). Proportions of females ranged 28.81–100%. Chronic diseases included chronic pain (*n* = 19, 43.18%), cancer (*n* = 7, 15.91%), arthritis (*n* = 6, 13.64%), cardiovascular disease (*n* = 4, 9.09%), diabetes (*n* = 2, 4.55%), HIV (*n* = 1, 2.27%), multiple sclerosis (*n* = 1, 2.27%), and different chronic diseases (*n* = 4, 9.09%). Comorbid physical or psychiatric conditions were reported in 23 (52.27%) studies. Complementary treatments for either physical or comorbid psychiatric conditions were reported in 22 (50.00%) studies. For details, see Supplementary Table 1.

Interventions across studies were predominantly delivered through web-based modules (*n* = 39, 88.64%), alongside videoconferences (*n* = 3, 6.82%) and mobile apps (*n* = 3, 6.82%); they were generally short in duration (<12 sessions)^19^ (*n* = 35, 79.55%) and guided^12^ (*n* = 32, 72.73%). Control groups included active (*n* = 21 studies, 47.73%) or non-active (*n* = 24, 54.55%) types. The active control condition included information/education (*k* = 10 comparisons), discussion forum (*k* = 5), relaxation (*k* = 2), attention control (scheduled contact) (*k* = 2), supportive therapy (*k* = 1), computerized cognitive remediation therapy (*k* = 1), and lifestyle management (*k* = 1). For details, see Supplementary Tables 1 and 4. The first follow-up was conducted 8–36 weeks after the intervention ended, whereas the last follow-up 12–48 weeks.

### Effectiveness of IM-CBT

**Fig. 2:**
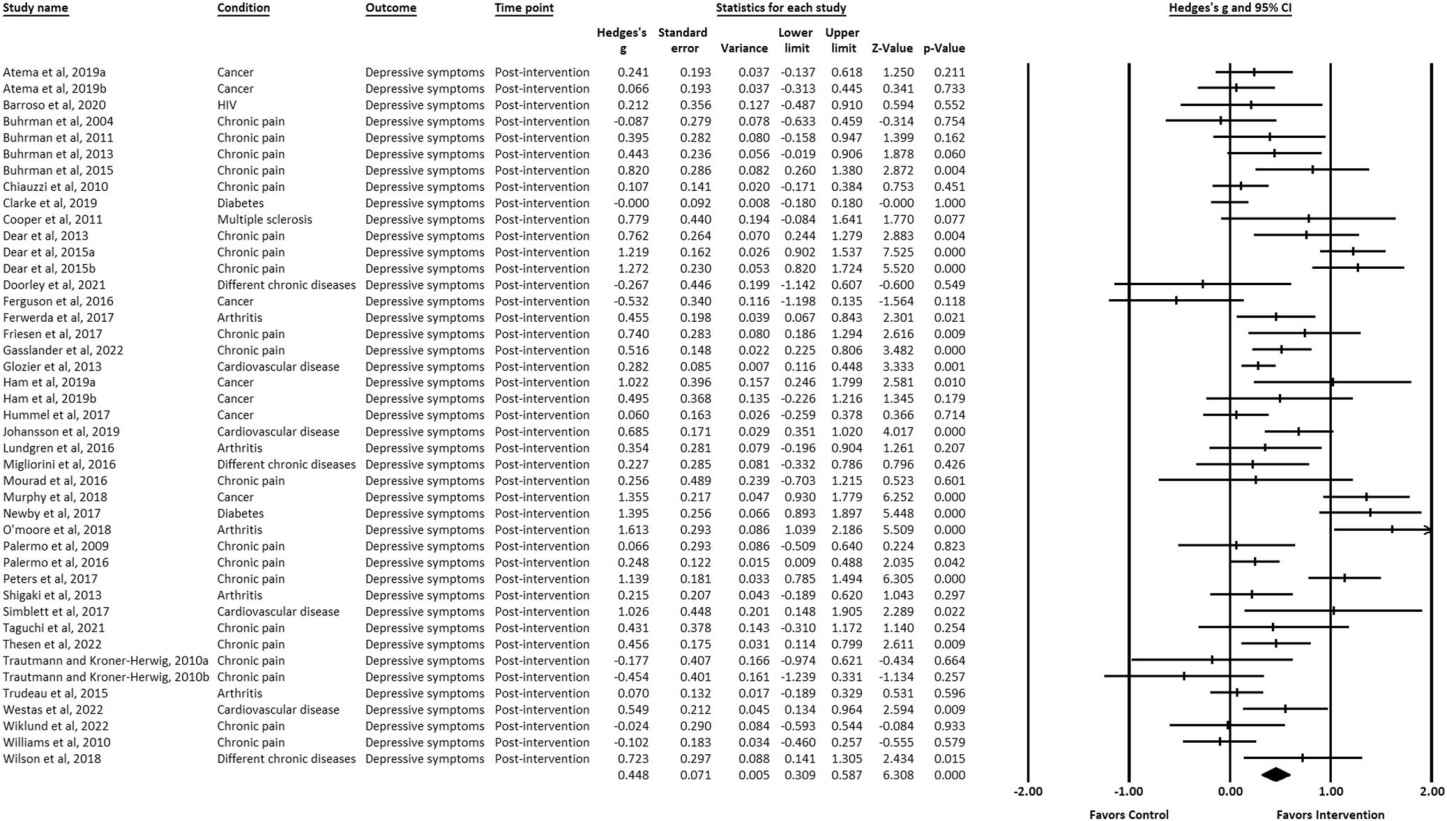
Forest plot for effect sizes of IM-CBT on depressive symptoms at post-intervention.

**Fig. 3:**
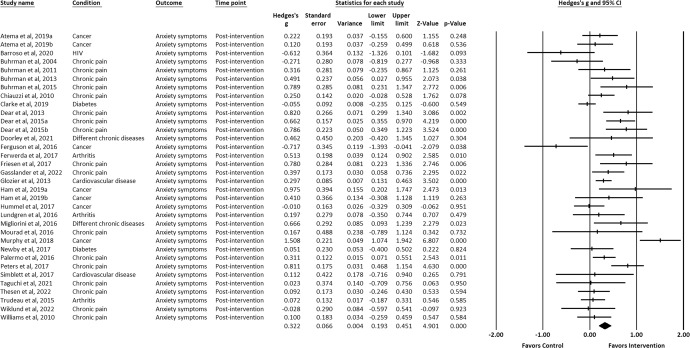
Forest plot for effect sizes of IM-CBT on anxiety symptoms at post-intervention.

**Fig. 4:**
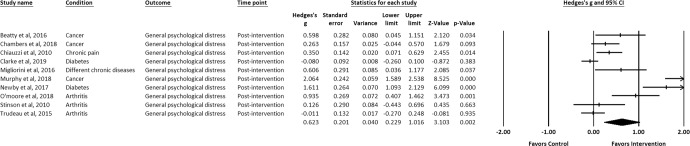
Forest plot for effect sizes of IM-CBT on general psychological distress at post-intervention.

**Fig. 5:**
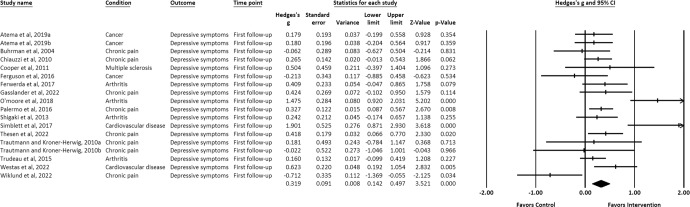
Forest plot for effect sizes of IM-CBT on depressive symptoms at first follow-up.

**Fig. 6:**
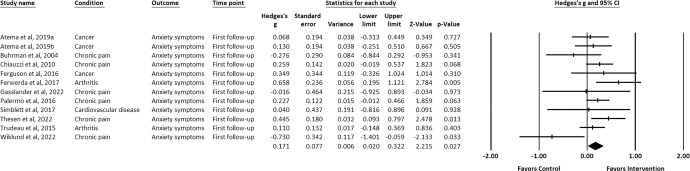
Forest plot for effect sizes of IM-CBT on anxiety symptoms at first follow-up.

**Fig. 7:**
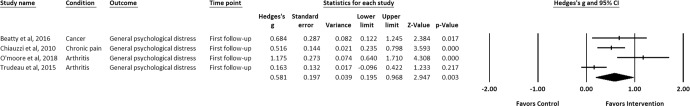
Forest plot for effect sizes of IM-CBT on general psychological distress at first follow-up.

**Fig. 8:**
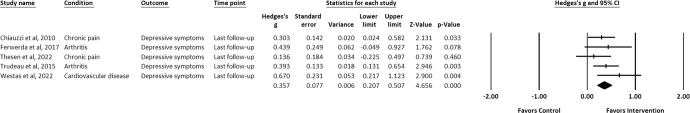
Forest plot for effect sizes of IM-CBT on depressive symptoms at last follow-up.

**Fig. 9:**
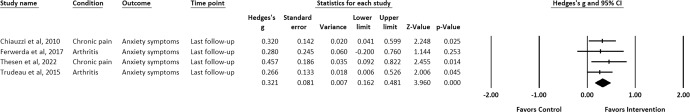
Forest plot for effect sizes of IM-CBT on anxiety symptoms at last follow-up.

**Fig. 10:**
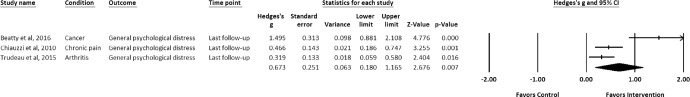
Forest plot for effect sizes of IM-CBT on general psychological distress at last follow-up.

IM-CBT exhibited a small-to-moderate effect on decreased depressive symptoms, anxiety symptoms, and general psychological distress across all timepoints: at post-intervention (depressive symptoms, *g* = 0.448, 95% CI [0.309, 0.587], *p* < 0.001; anxiety symptoms, *g* = 0.322, 95% CI [0.193, 0.451], *p* < 0.001; general psychological distress, *g* = 0.623, 95% CI [0.229, 1.016], *p* = 0.002) (Figs. 2–4)^21–34,36–39,41–64^, first follow-up (depressive symptoms, *g* = 0.319, 95% CI [0.142, 0.497], *p* < 0.001; anxiety symptoms, *g* = 0.171, 95% CI [0.020, 0.322], *p* = 0.027; general psychological distress, *g* = 0.581, 95% CI [0.195, 0.968], *p* = 0.003) (Figs. 5–7)^21,23,24,29,31,36,37,39,50,52,54,55,58–62^, and last-follow-up (depressive symptoms, *g* = 0.357, 95% CI [0.207, 0.507], *p* < 0.001; anxiety symptoms, *g* = 0.321, 95% CI [0.162, 0.481], *p* < 0.001; general psychological distress, *g* = 0.673, 95% CI [0.180, 1.165], *p* = 0.007) (Figs. 8–10)^23,29,37,58,60,61^.

The effects on decreased PTSD symptoms^23,39,40^ and combined depressive and anxiety symptoms^21,35,39,40^ were significant at follow-up(s) only: at first follow-up (PTSD symptoms, *g* = 0.867, 95% CI [0.453, 1.282], *p* < 0.001; combined depressive and anxiety symptoms, *g* = 0.241, 95% CI [0.020, 0.461], *p* = 0.032), and last-follow-up (PTSD symptoms, *g* = 0.576, 95% CI [0.024, 1.128], *p* = 0.041).

**Fig. 11:**
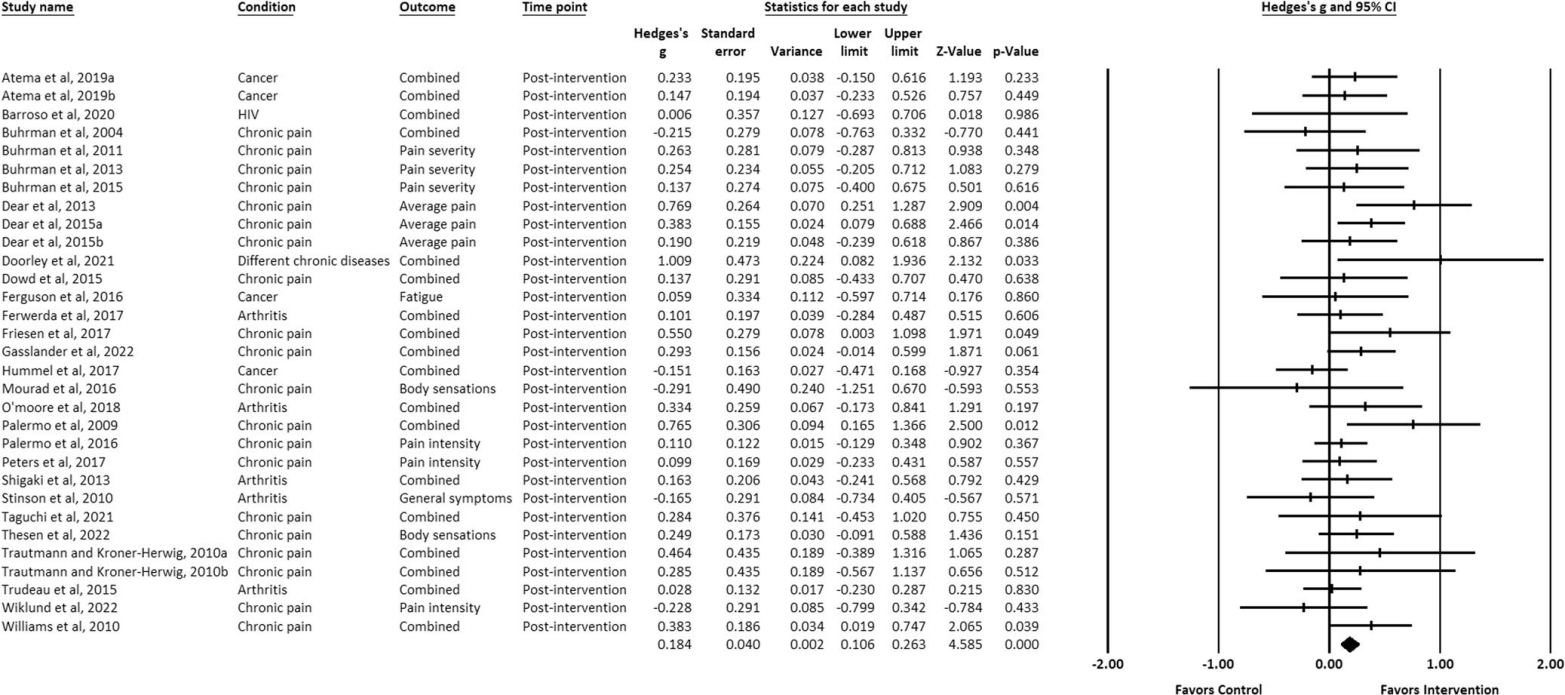
Forest plot for effect sizes of IM-CBT on physical symptoms at post-intervention. Note. “Combined” (under the column “Outcome”) indicates that multiple outcomes on physical symptoms were retrieved and averaged from the same comparison.

**Fig. 12:**
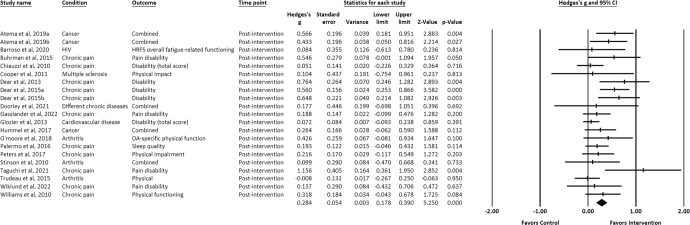
Forest plot for effect sizes of IM-CBT on functional impairment at post-intervention. Note. “Combined” (under the column “Outcome”) indicates that multiple outcomes on functional impairment were retrieved and averaged from the same comparison.

Effect sizes of the positive associations of IM-CBT with decreased physical symptoms (*g* = 0.184) (Fig. 11) and functional impairment (*g* = 0.284) (Fig. 12) were small-to-moderate and only at post-intervention^21,22,24–27,29,31–39,41,43,47,50–54,56–60,62,63^.

Results are summarized in Table 2. A complete list of all forest plots is available in Supplementary Figure 1. No significant differences in the effect of IM-CBT on decreased psychiatric outcomes were found across timepoints, whereas the effect on decreased physical distress was present only at post-intervention (Table 3).

**Table 2:** Pooled effect sizes of IM-CBT on psychiatric symptoms and physical distress at post-intervention and follow-ups (*n* = 44 studies).

Timepoint	Outcome domain	Outcome	*k*	Pooled *g* (95% CI)	*p*	*I*^2^ (%)	*Q*
Post-intervention	Psychiatric	Depressive symptoms	43	0.448 (0.309 to 0.587)	<0.001	78.654	196.757
		Anxiety symptoms	34	0.322 (0.193 to 0.451)	<0.001	70.634	112.376
		Depressive and anxiety symptoms	7	0.447(–0.029 to 0.922)	0.066	89.445	56.846
		Posttraumatic stress disorder (PTSD) symptoms	3	1.083 (–0.266 to 2.432)	0.116	92.235	25.757
		General psychological distress	10	0.623 (0.229 to 1.016)	0.002	91.721	108.709
	Physical	Physical symptoms	31	0.184 (0.106 to 0.263)	<0.001	3.600	31.120
		Functional impairment	21	0.284 (0.178 to 0.390)	<0.001	38.490	32.151
		Self-rated ill health	4	0.080 (–0.279 to 0.483)	0.664	53.830	6.498
		Objective physiological dysfunction	2	0.047 (–0.332 to 0.427)	0.807	0	0.922
First follow-up	Psychiatric	Depressive symptoms	18	0.319 (0.142 to 0.497)	<0.001	62.337	45.137
		Anxiety symptoms	12	0.171 (0.020 to 0.322)	0.027	36.981	17.455
		Depressive and anxiety symptoms	5	0.241 (0.020 to 0.461)	0.032	5.266	4.222
		Posttraumatic stress disorder (PTSD) symptoms	3	0.867 (0.453 to 1.282)	<0.001	0	0.434
		General psychological distress	4	0.581 (0.195 to 0.968)	0.003	76.278	12.647
	Physical	Physical symptoms	15	0.047 (–0.147 to 0.241)	0.636	62.615	37.448
		Functional impairment	9	0.182 (–0.039 to 0.403)	0.106	64.964	22.834
		Self-rated ill health	1	0.000 (–0.414 to 0.414)	>0.999	0	0
		Objective physiological dysfunction	0	–	–	–	–
Last follow-up	Psychiatric	Depressive symptoms	5	0.357 (0.207 to 0.507)	<0.001	0	3.602
		Anxiety symptoms	4	0.321 (0.162 to 0.481)	<0.001	0	0.735
		Depressive and anxiety symptoms	0	–	–	–	–
		Posttraumatic stress disorder (PTSD) symptoms	1	0.576 (0.024 to 1.128)	0.041	0	0
		General psychological distress	3	0.673 (0.180 to 1.165)	0.007	83.308	11.982
	Physical	Physical symptoms	3	0.191 (–0.003 to 0.384)	0.053	0	0.373
		Functional impairment	2	–0.016 (–0.205 to 0.174)	0.872	0	0.124
		Self-rated ill health	0	–	–	–	–
		Objective physiological dysfunction	0	–	–	–	–

*k* = Number of averaged effect sizes (to address the potential dependency issues, when multiple effect sizes were available from the same source, the moderator analyses were done based on the averaged effect sizes). The detailed forest plots with effect sizes from individual studies are available in Supplementary Figure 1. A table presenting pooled effect sizes from *n* = 58 studies (including 44 studies here and another 14 studies included in the Supplementary Information only) is available in Supplementary Table 8.

Definitions. “Physical symptoms” includes: arthritis symptoms, general symptoms, physical symptoms, menopausal symptoms, osteoarthritis (OA)-specific stiffness, fatigue, insomnia, HIV-related fatigue intensity, average pain, pain at rest, bodily pain, pain, pain intensity, pain now, pain severity, pain with activity, osteoarthritis (OA)-specific pain, headache intensity, bodily sensations, headache frequency, hot flush (HF) frequency, HIV-related total fatigue frequency, night sweats (NS) frequency; “Functional impairment” includes: disability, fine motor function, gross motor function, functional well-being, gross motor function, osteoarthritis (OA)-specific physical function, overall sexual functioning, physical function, HIV-related overall fatigue-related functioning, pain disability, physical impairment, physical impact, sleep quality; “Self-rated ill health” includes: general health, physical health, overall health (physical), physical well-being; “Objective physiological dysfunction” includes: Hemoglobin A1c, HIV viral load.

**Table 3:** Moderators of the effectiveness of IM-CBT on psychiatric symptoms and physical distress (*n* = 44 studies).

Moderator	Psychiatric symptoms				Physical distress			
	*k*	Statistic type	Statistic value (95% CI)	*p*	*k*	Statistic type	Statistic value (95% CI)	*p*
Model 1 Psychiatric symptoms								
Subgroup differences	–	*Q*-value	8.236	0.083	–	–	–	–
Depressive symptoms	43	Hedge’s *g*	0.462 (0.323 to 0.601)	<0.001	–	–	–	–
Anxiety symptoms	34	Hedge’s *g*	0.328 (0.199 to 0.456)	<0.001	–	–	–	–
Depressive and anxiety symptoms	7	Hedge’s *g*	0.489 (–0.023 to 1.001)	0.061	–	–	–	–
PTSD symptoms	3	Hedge’s *g*	0.904 (0.431 to 1.377)	<0.001	–	–	–	–
General psychological distress	10	Hedge’s *g*	0.693 (0.297 to 1.090)	0.001	–	–	–	–
Model 2 Physical distress								
Subgroup differences	–	–	–	–	–	*Q*-value	3.320	0.345
Physical symptoms	–	–	–	–	31	Hedge’s *g*	0.173 (0.076 to 0.271)	<0.001
Functional impairment	–	–	–	–	21	Hedge’s *g*	0.282 (0.164 to 0.400)	<0.001
Self-rated ill health	–	–	–	–	4	Hedge’s *g*	0.062 (–0.297 to 0.421)	0.735
Objective physiological dysfunction	–	–	–	–	2	Hedge’s *g*	0.047 (–0.332 to 0.427)	0.807
Model 3 Gender								
Female percentage (28.81%–100.00%)	48	Coefficient	0.002 (–0.005 to 0.009)	0.521	36	Coefficient	0.003 (–0.002 to 0.008)	0.177
Model 4 Chronic disease								
Subgroup differences	–	*Q*-value	4.560	0.714	–	*Q*-value	2.927	0.892
Chronic pain	21	Hedge’s *g*	0.391 (0.216 to 0.566)	<0.001	21	Hedge’s *g*	0.232 (0.111 to 0.353)	<0.001
Cancer	9	Hedge’s *g*	0.495 (0.099 to 0.892)	0.014	4	Hedge’s *g*	0.162 (–0.046 to 0.370)	0.128
Arthritis	6	Hedge’s *g*	0.402 (0.094 to 0.709)	0.010	5	Hedge’s *g*	0.165 (–0.042 to 0.373)	0.119
Cardiovascular disease	4	Hedge’s *g*	0.504 (0.245 to 0.764)	<0.001	1	Hedge’s *g*	0.072 (–0.093 to 0.238)	0.391
Diabetes	2	Hedge’s *g*	0.461 (–0.580 to 1.503)	0.385	1	Hedge’s *g*	0.024 (–0.427 to 0.475)	0.916
Multiple sclerosis	1	Hedge’s *g*	0.641 (–0.240 to 1.523)	0.154	1	Hedge’s *g*	0.278 (–0.587 to 1.142)	0.529
HIV	1	Hedge’s *g*	–0.200 (–0.906 to 0.506)	0.578	1	Hedge’s *g*	0.154 (–0.546 to 0.855)	0.666
Different chronic diseases	4	Hedge’s *g*	0.566 (0.230 to 0.902)	0.001	2	Hedge’s *g*	–0.009 (–1.070 to 1.051)	0.986
Model 5 Physical or psychiatric comorbidity								
Subgroup differences	–	*Q*-value	0.033	0.857	–	*Q*-value	0.048	0.826
Yes	24	Hedge’s *g*	0.441 (0.255 to 0.628)	<0.001	16	Hedge’s *g*	0.171 (0.049 to 0.293)	0.006
No	24	Hedge’s *g*	0.419 (0.264 to 0.573)	<0.001	20	Hedge’s *g*	0.190 (0.070 to 0.309)	0.002
Model 6 Medication received for physical condition(s)								
Subgroup differences	–	*Q*-value	0.114	0.735	–	*Q*-value	0.089	0.766
Yes	25	Hedge’s *g*	0.400 (0.234 to 0.647)	<0.001	18	Hedge’s *g*	0.195 (0.077 to 0.314)	0.001
No	23	Hedge’s *g*	0.400 (0.283 to 0.517)	<0.001	18	Hedge’s *g*	0.170 (0.046 to 0.293)	0.007
Model 7 Surgery received for physical condition(s)								
Subgroup differences	–	*Q*-value	0.268	0.605	–	*Q*-value	0.367	0.545
Yes	8	Hedge’s *g*	0.537 (0.069 to 1.005)	0.024	4	Hedge’s *g*	0.095 (–0.201 to 0.391)	0.530
No	40	Hedge’s *g*	0.410 (0.291 to 0.529)	<0.001	32	Hedge’s *g*	0.191 (0.102 to 0.279)	<0.001
Model 8 Supplement and/or other received for physical condition(s)								
Subgroup differences	–	*Q*-value	0.090	0.764	–	*Q*-value	0.040	0.841
Yes	6	Hedge’s *g*	0.478 (0.094 to 0.862)	0.015	6	Hedge’s *g*	0.197 (–0.072 to 0.466)	0.151
No	42	Hedge’s *g*	0.416 (0.290 to 0.543)	<0.001	30	Hedge’s *g*	0.168 (0.082 to 0.254)	<0.001
Model 9 Medication received for psychiatric condition(s)								
Subgroup differences	–	*Q*-value	3.099	0.078	–	*Q*-value	1.478	0.224
Yes	13	Hedge’s *g*	0.641 (0.300 to 0.982)	<0.001	8	Hedge’s *g*	0.296 (0.055 to 0.536)	0.016
No	35	Hedge’s *g*	0.323 (0.224 to 0.421)	<0.001	28	Hedge’s *g*	0.139 (0.061 to 0.216)	<0.001
Model 10 Psychotherapy (non-CBT) received for psychiatric condition(s)								
Subgroup differences	–	*Q*-value	0.683	0.408	–	*Q*-value	0.476	0.490
Yes	3	Hedge’s *g*	0.893 (–0.280 to 2.066)	0.136	1	Hedge’s *g*	0.024 (–0.427 to 0.475)	0.916
No	45	Hedge’s *g*	0.396 (0.289 to 0.503)	<0.001	35	Hedge’s *g*	0.186 (0.100 to 0.272)	<0.001
Model 11 Main intervention delivery format								
Subgroup differences	–	*Q*-value	4.030	0.133	–	*Q*-value	0.063	0.969
Videoconference	3	Hedge’s *g*	–0.013 (–0.445 to 0.419)	0.953	3	Hedge’s *g*	0.254 (–0.340 to 0.847)	0.402
Web-based	42	Hedge’s *g*	0.448 (0.321 to 0.576)	<0.001	32	Hedge’s *g*	0.180 (0.093 to 0.267)	<0.001
Mobile app	3	Hedge’s *g*	0.403 (–0.272 to 1.077)	0.242	1	Hedge’s *g*	0.154 (–0.546 to 0.855)	0.666
Model 12 Guidance								
Subgroup differences	–	*Q*-value	0.055	0.814	–	*Q*-value	0.452	0.501
Guided	33	Hedge’s *g*	0.434 (0.284 to 0.584)	<0.001	25	Hedge’s *g*	0.205 (0.099 to 0.311)	<0.001
Unguided	15	Hedge’s *g*	0.404 (0.204 to 0.603)	<0.001	11	Hedge’s *g*	0.144 (0.004 to 0.285)	0.044
Model 13 Intervention length (no. of sessions)								
Subgroup differences	–	*Q*-value	6.772	0.009	–	*Q*-value	1.685	0.194
Medium/long (≥12 sessions)	10	Hedge’s *g*	0.186 (0.014 to 0.358)	0.034	7	Hedge’s *g*	0.082 (–0.088 to 0.252)	0.343
Short (<12 sessions)	38	Hedge’s *g*	0.481 (0.341 to 0.621)	<0.001	29	Hedge’s *g*	0.211 (0.116 to 0.307)	<0.001
Model 14 Intervention duration (weeks)								
Duration in weeks (4–26 weeks)	48	Coefficient	0.005 (–0.021 to 0.032)	0.697	36	Coefficient	0.005 (–0.010 to 0.019)	0.530
Model 15 Intervention session number								
Session number (4–48 sessions)	48	Coefficient	–0.001 (–0.018 to 0.015)	0.885	36	Coefficient	–0.021 (–0.039 to –0.002)	0.034
Model 16 Intervention frequency								
Sessions per week (0.15–4.80)	48	Coefficient	–0.044 (–0.207 to 0.119)	0.597	36	Coefficient	–0.237 (–0.387 to –0.087)	0.002
Model 17 Total number of therapeutic elements								
Number of components (2–5)	48	Coefficient	0.063 (–0.087 to 0.213)	0.407	36	Coefficient	0.032 (–0.061 to 0.125)	0.501
Model 18 Intervention has behavioral modification element								
Subgroup differences	–	*Q*-value	19.498	<0.001	–	*Q*-value	–	–
Yes	47	Hedge’s *g*	0.442 (0.322 to 0.561)	<0.001	36	Hedge’s *g*	0.181 (0.097 to 0.265)	<0.001
No	1	Hedge’s *g*	–0.045 (–0.225 to 0.135)	0.624	–	Hedge’s *g*	–	–
Model 19 Intervention has cognitive restructuring element								
Subgroup differences	–	*Q*-value	0.486	0.486	–	*Q*-value	2.016	0.156
Yes	34	Hedge’s *g*	0.454 (0.298 to 0.610)	<0.001	27	Hedge’s *g*	0.212 (0.110 to 0.313)	<0.001
No	14	Hedge’s *g*	0.373 (0.208 to 0.539)	<0.001	9	Hedge’s *g*	0.078 (–0.075 to 0.232)	0.318
Model 20 Intervention has problem-solving element								
Subgroup differences	–	*Q*-value	1.508	0.220	–	*Q*-value	3.714	0.054
Yes	47	Hedge’s *g*	0.433 (0.311 to 0.556)	<0.001	35	Hedge’s *g*	0.170 (0.088 to 0.251)	<0.001
No	1	Hedge’s *g*	0.066 (–0.509 to 0.640)	0.823	1	Hedge’s *g*	0.765 (0.165 to 1.366)	0.012
Model 21 Intervention has psychoeducation element								
Subgroup differences	–	*Q*-value	0.873	0.350	–	*Q*-value	0.034	0.854
Yes	41	Hedge’s *g*	0.404 (0.271 to 0.537)	<0.001	31	Hedge’s *g*	0.176 (0.099 to 0.253)	<0.001
No	7	Hedge’s *g*	0.554 (0.269 to 0.840)	<0.001	5	Hedge’s *g*	0.213 (–0.179 to 0.605)	0.286
Model 22 Intervention has mindfulness element								
Subgroup differences	–	*Q*-value	0.647	0.421	–	*Q*-value	0.245	0.621
Yes	31	Hedge’s *g*	0.460 (0.305 to 0.614)	<0.001	24	Hedge’s *g*	0.173 (0.094 to 0.252)	<0.001
No	17	Hedge’s *g*	0.357 (0.162 to 0.553)	<0.001	12	Hedge’s *g*	0.116 (–0.097 to 0.328)	0.286
Model 23 Measurement timepoint								
Subgroup differences	–	*Q*-value	0.976	0.614	–	*Q*-value	2.260	0.323
Last follow-up (12–48 weeks)	6	Hedge’s *g*	0.431 (0.258 to 0.605)	<0.001	4	Hedge’s *g*	0.088 (–0.071 to 0.246)	0.278
First follow-up (8–36 weeks)	21	Hedge’s *g*	0.328 (0.176 to 0.480)	<0.001	17	Hedge’s *g*	0.075 (–0.858 to 0.391)	0.391
Post-intervention	48	Hedge’s *g*	0.411 (0.288 to 0.534)	<0.001	36	Hedge’s *g*	0.185 (0.118 to 0.252)	<0.001
Model 24 Follow-up duration after intervention (weeks)								
Duration in weeks (8–48 weeks)	27	Coefficient	–0.002 (–0.012 to 0.009)	0.745	21	Coefficient	–0.003 (–0.014 to 0.007)	0.560
Model 25 Control group type								
Subgroup differences	–	*Q*-value	3.941	0.047	–	*Q*-value	6.177	0.013
Active	22	Hedge’s *g*	0.299 (0.160 to 0.437)	<0.001	13	Hedge’s *g*	0.049 (–0.064 to 0.163)	0.394
Non-active	26	Hedge’s *g*	0.535 (0.347 to 0.723)	<0.001	23	Hedge’s *g*	0.243 (0.141 to 0.346)	<0.001
Model 26 Overall risk of bias								
Subgroup differences	–	*Q*-value	3.855	0.146	–	*Q*-value	0.274	0.872
High risk	19	Hedge’s *g*	0.306 (0.145 to 0.467)	<0.001	15	Hedge’s *g*	0.159 (0.047 to 0.270)	0.005
Some concerns	26	Hedge’s *g*	0.530 (0.327 to 0.732)	<0.001	17	Hedge’s *g*	0.211 (0.048 to 0.375)	0.011
Low risk	3	Hedge’s *g*	0.301 (0.170 to 0.431)	<0.001	4	Hedge’s *g*	0.177 (–0.013 to 0.367)	0.068
Model 27 Attrition rate at post-intervention								
Subgroup differences	–	*Q*-value	1.223	0.543	–	*Q*-value	2.199	0.333
High (>20%)	8	Hedge’s *g*	0.424 (0.130 to 0.717)	0.005	5	Hedge’s *g*	0.068 (–0.136 to 0.271)	0.517
Moderate (5–20%)	34	Hedge’s *g*	0.443 (0.284 to 0.602)	<0.001	25	Hedge’s *g*	0.177 (0.065 to 0.288)	0.002
Low (<5%)	6	Hedge’s *g*	0.308 (0.123 to 0.494)	0.001	6	Hedge’s *g*	0.274 (0.092 to 0.457)	0.003
Model 28 Intention-to-treat analysis								
Subgroup differences	–	*Q*-value	0.242	0.623	–	*Q*-value	0.321	0.571
Yes	36	Hedge’s *g*	0.439 (0.294 to 0.583)	<0.001	28	Hedge’s *g*	0.181 (0.106 to 0.255)	<0.001
No	12	Hedge’s *g*	0.377 (0.178 to 0.576)	<0.001	8	Hedge’s *g*	0.091 (–0.209 to 0.392)	0.552

*k* = Number of averaged effect sizes (to address the potential dependency issues, when multiple effect sizes were available from the same source, the moderator analyses were done based on the averaged effect sizes). The moderator analysis for “Follow-up duration after intervention” was only analyzed upon follow-up data points. A table presenting moderator analyses for *n* = 58 studies (including 44 studies here and another 14 studies included in the Supplementary Information only) is available in Supplementary Table 9.

Definitions. “Guidance” was defined as: “Guided” refers to therapists’ therapeutic input, including active provision of intervention, feedback, and/or support; “Unguided” refers to technical/adherence or other non-specified assistance only^12^. “Intervention duration” was defined as: <12 sessions = short, 12–16 sessions = medium, >16 sessions = long^19^. “Non-active” control group included waitlist control (WLC) and treatment-as-usual (TAU) / standard care (SC); “Active” control group included information/education (*k* = 10), discussion forum (*k* = 5), relaxation (*k* = 2), attention control (scheduled contact) (*k* = 2), supportive therapy (*k* = 1), computerized cognitive remediation therapy (*k* = 1), and lifestyle management (*k* = 1). “Attrition rate at post-intervention” was defined as: <5% = low, 5–20% = moderate, and >20% = high^106^.

### Reciprocity between changes in psychiatric symptoms and changes in physical distress

**Fig. 13:**
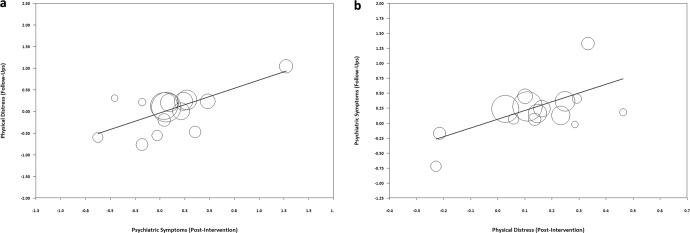
Regression results between effect sizes of improvements in psychiatric symptoms and physical distress (*n* = 44 studies). Note. **a** The regression of averaged effect sizes of physical distress (at follow-ups) on averaged effect sizes of psychiatric symptoms (at post-intervention). **b** The regression of averaged effect sizes of psychiatric symptoms (at follow-ups) on averaged effect sizes of physical distress (at post-intervention). A figure with regressions for *n* = 58 studies (including 44 studies here and another 14 studies included in the Supplementary Information only) is available in Supplementary Fig. 3.

Decreased psychiatric symptoms at post-intervention prospectively predicted decreased physical distress at follow-ups, *B* = 0.761, 95% CI [0.405, 1.118], *p* < 0.001^21,24,35–37,39,50,51,54,58–60,62^. Likewise, decreased physical distress at post-intervention prospectively predicted decreased psychiatric symptoms at follow-ups, *B* = 1.456, 95% CI [0.597, 2.314], *p* = 0.001^21,24,35–37,39,50,52,54,58–60,62^. The results showed bidirectional positive associations (Fig. 13).

### Core therapeutic elements of IM-CBT affecting clinical responses

Therapeutic elements within individual studies are summarized in Supplementary Table 3. With the exception of two studies without either behavioral modification (473 patients)^30^ or problem-solving (45 patients)^51^, the majority of studies (*k*s = 46 comparisons) included both elements. IM-CBT was effective for reducing psychiatric symptoms when the protocols included behavioral modification (*g* = 0.442, 95% CI [0.322, 0.561], *p* < 0.001) or problem-solving (*g* = 0.433, 95% CI [0.311, 0.556], *p* < 0.001). IM-CBT protocols were equally effective for reducing psychiatric symptoms with or without cognitive restructuring, psychoeducation, or mindfulness (all *p*s ≤ 0.001). Meanwhile, IM-CBT significantly reduced physical distress when the protocols included cognitive restructuring (*g* = 0.212, *p* < 0.001)^21,24–26,31–33,35–38,41,43,49–53,56,57,59,60,63,64^, psychoeducation (*g* = 0.176, *p* < 0.001)^21,22,24–27,31–36,38,39,41,43,47,49,52–54,56–60,62,63^, or mindfulness (*g* = 0.173, *p* < 0.001)^21,22,25–27,29,32–35,38,39,41,43,49,53,54,56,57,59,60^, but the effects were non-significant without these elements. The effects on reducing psychiatric symptoms or physical distress were independent of the total number of therapeutic elements (*p*s ≥ 0.407). Results are summarized in Table 3.

### Likely candidates for patient-related and treatment-related moderators

IM-CBT related to decreased psychiatric symptoms among patients diagnosed with chronic pain (*g* = 0.391, *p* < 0.001)^24–27,29,32,33,35,38,39,47,51–53,57–59,62,63^, cancer (*g* = 0.495, *p* = 0.014)^21,23,28,36,42,43,48^, arthritis (*g* = 0.402, *p* = 0.010)^37,45,50,54,56,60^, cardiovascular disease (*g* = 0.504, *p* < 0.001)^41,44,55,61^, and different chronic diseases (*g* = 0.566, *p* = 0.001)^34,40,46,64^, but not those with diabetes, multiple sclerosis, or HIV (*p*s ≥ 0.154). Decreased symptoms were reported by patients who were not on concurrent psychotherapies for psychiatric condition(s) (*g* = 0.396, *p* < 0.001)^21–29,31–47,50–64^ but not those receiving psychotherapies (*p* = 0.136). IM-CBT effects were observed independent of patients’ gender and presence of comorbidity (*p*s ≥ 0.287). Age as a potential moderator was not analyzed because most studies were conducted among people with a wide range of age (11–91 years, information retrievable from *n* = 24 studies)^23,27,29,31–35,37–40,42,44–47,50–53,57,58,62^ while only mean age (*SD*) was available for all included studies.

The effect sizes for the IM-CBT effects on decreased psychiatric symptoms were larger with fewer than 12 sessions (*g* = 0.481, *p* < 0.001)^21–29,31–34,36–40,44–55,58–62^ relative to ≥12 sessions (*g* = 0.186, *p* = 0.034) but independent of guidance (*p* = 0.814). Effects were significant only among interventions delivered through web-based modules (*g* = 0.448, *p* < 0.001), relative to interventions delivered through videoconference^34,36,57^ and mobile app^22,40,42^. Results are summarized in Table 3. Factors associated with stronger IM-CBT effect sizes on decreased physical distress included specific diagnosis (i.e., chronic pain), absence of complementary treatments for physical/psychiatric conditions, fewer than 12 sessions, and lower frequency of the intervention (Table 3).

### Level of confidence in the evidence

IM-CBT effects on psychiatric symptoms were not influenced by methodological factors including overall risk of bias, attrition at post-intervention, and utilization of intention-to-treat analysis, but the effect sizes were larger when comparisons involved non-active (*g* = 0.535, *p* < 0.001) than active (*g* = 0.299, *p* < 0.001) control groups, *p* = 0.047. Meanwhile, the effects on physical distress were significant only when studies showed some-to-high risks of overall bias, low-to-moderate (≤20%) attrition rate, utilization of intention-to-treat analysis, and/or non-active control groups (Table 3). No significant publication bias was found on most outcomes among the pooled studies (Supplementary Table 7 and Supplementary Fig. 2). We performed a sensitivity analysis by replicating all analyses after including 14 studies with non-synchronized CBTs delivered through telephone or self-help materials and found largely consistent results (Supplementary Tables 8–9 and Supplementary Fig. 3).

## Discussion

This study is a comprehensive and up-to-date systematic review and meta-analysis on the effects of internet-based and mobile-based Cognitive Behavioral Therapy (IM-CBT) on psychiatric symptoms, physical distress, and the reciprocity between improved mental and physical health outcomes among people with chronic diseases. We specifically investigated the therapeutic elements and effect moderators. Our conclusions were based on 44 RCTs (48 comparisons) conducted across 11 countries, with a total of 5077 patients affected by seven different chronic diseases. We found immediate and/or sustained effects of IM-CBT on reducing psychiatric symptoms and physical distress, with improved psychiatric symptoms and physical distress prospectively positively predicting each other. Behavioral modification and problem-solving benefited psychiatric symptoms, whereas cognitive restructuring, psychoeducation, and mindfulness benefited physical distress. Diagnoses of chronic diseases and lower intervention intensity moderated the clinical benefits of IM-CBT, suggesting the need to investigate its impact in more diverse chronic diseases and the cautions in applying different therapeutic elements among different patients.

This meta-analysis went beyond previous studies by robustly validating the clinical benefits of IM-CBT with more types of chronic diseases, more varied psychiatric and physical outcomes, and longer durations of follow-up. Telemedicine and digital psychotherapeutic interventions^9,65–67^ have become more common in recent years^68,69^. With comparable effectiveness as face-to-face CBT^14,15^, IM-CBT could contribute added values over its conventional counterparts^9–11,65,66^.

This meta-analysis confirmed the benefits of IM-CBT on the mental health of people with chronic diseases. The effectiveness of CBT for alleviating depressive and/or anxiety symptoms has been attested among people with chronic pain^70^, rheumatoid arthritis^71^, COPD^72^, cardiovascular disease^73^, and kidney disease^74^. Adding to previous evidence on the IM-CBT effects on decreased depressive and anxiety symptoms, this study reported some of the first evidence that suggests sustainability of the positive effect over time and across other psychiatric conditions including PTSD symptoms and general psychological distress. The effects remained significant controlling for study quality. It is important to note, however, that included studies focused on depression, anxiety, PTSD, and general psychological distress only although we intended to review studies with all kinds of psychiatric conditions. Beyond the conventional IM-CBT approaches examined within the current review, a previous meta-analysis on technology-based acceptance and commitment therapy (20 articles/interventions)^75^ reported intervention effects for functioning and acceptance-related outcomes, whereas another systematic review on internet-based mindfulness-based interventions (11 articles on 10 interventions)^76^ reported intervention effects for psychiatric symptoms, coping, and/or quality of life. It warrants further investigation, however, whether the clinical benefits of conventional IM-CBT and its extensions are uniformly comparable or domain-specific^70,77^. Taken together, current and previous evidence generally supported the potential of IM-CBT and its third-wave extensions among patients with chronic diseases.

This study assessed comprehensive dimensions of physical distress, namely physical symptoms, functional impairment, self-rated ill health, and objective physiological dysfunction. Existing evidence on physical health benefits of CBT in chronic diseases has been relatively mixed – both positive^70,71,78,79^ and null^72,77^ effects have been reported among limited scopes of chronic diseases. Similarly, mixed findings have been identified on the physical health benefits of IM-CBT^19,20^. Although our moderator analyses suggested that IM-CBT could ameliorate physical symptoms and functional impairment, the significant effects could be attributable to methodological factors such as comparisons with non-active control groups^21,24,25,31–33,37–39,43,47,49–52,54,57,58,60,63^ and some-to-high risk of bias^21,22,24–27,29,31–36,38,39,43,47,49,50,53,54,56–60,62–64^. Non-significant effects could be attributable to high attrition^26,35,36,39,53^. Additionally, in order to obtain a complete picture on IM-CBT effects on physical distress, our current analysis maximally included all available data categorized under the four pre-specified subcategories, despite potential heterogeneity across specific outcomes. Further meta-analytic reviews are therefore warranted to look into IM-CBT effects on specific individual outcomes under physical distress.

Our findings showed that IM-CBT-related decrease in psychiatric symptoms and physical distress positively predicted each other in the long run, adding to existing cross-sectional evidence on the positive associations in previous empirical studies or meta-analyses^19,20,70–72,77–80^. The reciprocity suggested that the two dimensions of health are complementary to and benefit each other in the long run. Common psychiatric and physical conditions share etiology and maintenance factors^5^. Symptom and treatment management plans could consider psychiatric and physical conditions as a larger syndrome towards a holistic symptom management for people with chronic diseases^81^.

While CBT practically involves skill sets that could be theoretically classified into different categories^82^, most if not all previous studies overlooked the heterogeneity in therapeutic elements across interventions that share the same label of CBT^18–20^. This could limit a full understanding on the therapeutic mechanism(s) of IM-CBT (or CBT in general) responsible for clinical benefits on physical and mental health^83^.

We observed that two therapeutic elements, namely behavioral modification and problem-solving, were most commonly adopted across included RCTs (i.e., 42 out of the 44 studies included both). As such, cautions are warranted in interpreting these two factors as potential moderators of IM-CBT effects on psychiatric symptoms. While our analyses could not fully confirm on an empirical level that the two components are necessary conditions to ensure the benefits on physical and mental health of people with chronic diseases, theoretically, the clinical implications of behavioral modification and problem-solving have been documented in existing literature. For example, subordinate strategies within behavioral modification such as behavioral contracting and physical exercise could enhance activity level and healthy lifestyle, which in turn serve as protective factors of mental health^5,84^. Problem-solving, denoting systematic procedures to identify and address everyday life problems and enhance coping skills, has been found to decrease depressive symptoms among older adults with physical conditions^85^ and among psychiatric patients in the primary care setting^86^.

Cognitive restructuring, psychoeducation, and mindfulness were shown to be important therapeutic components for reducing physical distress. Cognitive restructuring replaces negative and inaccurate thoughts with more realistic and adaptive ones^87^. Relatedly, psychoeducation equips people with knowledge on chronic diseases and guides them to be aware of disease-related cognition and behaviors^88^. Both could increase health literacy and relieve psychological burden, leaving these people with more motivation and energy necessary for symptom management such as medication adherence and health-promoting behaviors^3^. In addition, mindfulness, as the ability or practice to observe one’s present sensations, thoughts, and feelings with an open and nonjudgmental attitude^89^, has been found to improve pain and fatigue, blood pressure, and weight control among people with different chronic diseases, although uncertainties exist in its mechanism, variability, and consistency across different modalities^90^.

The effects of IM-CBT in reducing psychiatric symptoms were more established among chronic pain, cancer, arthritis, and cardiovascular disease, but not diabetes, multiple sclerosis, and HIV. However, it should be noted that the latter three conditions have been investigated by fewer studies^22,30,31,49^.

The significant IM-CBT effects among interventions delivered via web-based modules but not videoconferences and mobile apps could be due to the fact that it was the predominant format adopted across eligible studies. However, because there were few studies on interventions delivered via videoconferences and mobile apps, which also tended to be less methodologically reliable (i.e., absence of intention-to-treat analysis and/or high attrition rates in most of them) compared to those delivered via web-based modules, we were not able to fully assess the impact of delivery platform on IM-CBT effects. More systematic investigation is needed on whether there is true advantage of delivering IM-CBT over particular types of platforms.

Surprisingly, the effects of IM-CBT on reducing psychiatric symptoms and physical distress were stronger with fewer sessions (<12 sessions) and thus shorter intervention durations. The effects for physical outcomes were similarly contingent upon fewer intervention sessions and lower intervention frequency. We followed these up with chi-squared tests, and noticed that on a methodological level, interventions with longer duration (≥12 sessions) and higher frequency tended to include no guidance (i.e., absence of therapists’ active provision of intervention, feedback, and/or support). Interventions with higher frequency were also more likely to include active control groups (e.g., information/education, discussion forum), and interventions with more sessions were more likely to target patients with physical and/or psychiatric comorbidity. These variations across the RCTs in this review in terms of design and quality suggest the importance of considering the multidimensional sources of therapeutic benefits. We found that active control group was a significant moderator. Based on the common factors theory^91^, a part of the IM-CBT effects could be protocol-nonspecific, and thus frequent engagement in the active control activities could be inversely related to psychiatric symptoms or physical distress over a period of time. In addition, our findings could call for more attention and empirical investigation to reconsider whether the effects of IM-CBT vary, positively, as functions of treatment duration and/or frequency. The association between intervention duration (number of sessions) and outcome could be curvilinear instead of linear, meaning a possible diminishing marginal benefit after an optimal number of sessions^92^. Our findings were indeed consistent with previous evidence suggesting lower dose as a cost-effective design^93,94^. Short intervention with frequent breaks has been suggested to be useful for accommodating fatigue in CBT for adolescents with chronic diseases^88^. Lower intervention intensity has also been recommended for people with poorer general health^95^, such as those with chronic diseases in the current meta-analysis. Frequent reminders on the intervention could inadvertently result in notification fatigue and increase non-adherence that has been observed in digital interventions among patients with chronic diseases^65^. In the current study, we observed that non-adherence (different from attrition) information was insufficiently reported and thus we could not include this variable in the formal analyses. Taken together, these observations invite an open discussion on optimizing the prescription of IM-CBT in order to maximize its clinical benefits for patients with chronic diseases.

This quantitative synthesis considered a wide range of chronic diseases and examined a large number of psychiatric and physical outcomes within IM-CBT for people with chronic diseases, as well as the positive prospective associations between physical and mental health outcomes. Effective individual therapeutic elements for reducing psychiatric symptoms and physical distress were identified, and patient-related/treatment-related moderators affecting the clinical responses were examined. Our evidence points to a clear direction for developing a holistic support care service for these people.

This meta-analysis has some limitations. We pooled the results despite the technical and clinical variations that exist across the included studies. Effect sizes for physical distress were synthesized under four subcategories (i.e., physical symptoms, functional impairment, self-rated ill health, objective physiological dysfunction) although there could be disparity in specific outcomes under each subcategory. Our moderator analyses were conducted on composite constructs of psychiatric symptoms and physical distress instead of the specific constructs. These procedures were applied in order to maximize the number of comparisons. Still, the pooled effect sizes and the moderator analyses might be restricted by the existing number of studies on certain outcomes or features. Some outcomes at follow-up timepoints were missing for synthesis, and differences across subgroups could be left undetected due to a lack of statistical power in the analysis of small samples. Finally, the existing evidence base is biased towards high-income countries/regions, restricting generalizability of the findings to less developed parts of the world.

In conclusion, internet-based and mobile-based cognitive behavioral therapy (IM-CBT) could be implemented in clinical settings in order to produce meaningful benefits on reducing psychiatric symptoms and physical distress among patients with chronic diseases. It is likely that the positive effects of IM-CBT on physical and mental health reciprocally benefit each other in the long run. IM-CBT could be particularly beneficial for people within some chronic diseases, while specific therapeutic elements could be key drivers of clinical benefits. It is important for medical scientists and clinicians to consider the fundamental driving forces of positive therapeutic changes in patients, as quality matters more than quantity in IM-CBT. The present findings could also be applicable to psychological services amid large-scale disasters, such as the COVID-19 pandemic, natural hazards, and wars, when physical comorbidities are more likely, restrictions are put on mobility, or the physical environment is not conducive to face-to-face interventions.

## Method

### Search strategy and selection criteria

This systematic review and meta-analysis was conducted according to the Preferred Reporting Items for Systematic review and Meta-analysis (PRISMA) guidelines^96^, and was pre-registered on PROSPERO (CRD42022265738). Any deviations were outlined and explained in Supplementary Note 1. Searches were performed in CINAHL of Systematic Reviews, MEDLINE, PsycINFO, PubMed, and Web of Science from inception through June 1, 2022, using combined variations of the following keyword categories: *chronic diseases*, *cognitive behavioral therapy*, *psychiatric symptoms*, *study design*. The detailed search algorithm is documented in Supplementary Note 2.

E.T.F.Y., T.K.L., and P.B.S. selected the articles and extracted data; disagreements were resolved through discussion with T.J.T., L.K.Y.M., and W.K.H. Only English articles published in peer-reviewed journals were considered. The current study reviewed randomized clinical trials that compared psychiatric symptoms between IM-CBT and non-CBT control condition(s) among patients diagnosed with chronic diseases listed on ICD-11 for ≥3 months. Because it is not quite possible to include the great variety of chronic diseases in one single systematic review/meta-analysis, we generated a list of common chronic diseases by referring to leading causes of disability-adjusted life years in the Global Burden of Disease Study 2015 in The Lancet^1^. This study was set out to focus on the more conventional types of CBT, which focus more on modifying and controlling behaviors, thoughts and emotions, relative to the third-wave extensions, which alternatively focus more on acceptance and mindfulness approaches^97^. In practice, their boundaries could be less clear-cut, and therefore in cases where interventions included a mix of cognitive-behavioral and third-wave elements, our key criterion to decide whether the interventions were eligible was whether they were predominantly defined by the cognitive and/or behavioral elements as opposed to the third-wave elements. Studies were also excluded if the treatment group contained any in-person psychosocial interventions.

### Quality assessment

Included articles were assessed by E.T.F.Y., T.K.L., and P.B.S. using the revised Cochrane risk-of-bias tool for randomized trials (RoB 2)^98^, and were categorized into low risk, some concerns, or high risk (Supplementary Table 6).

### Outcome measures

Primary outcomes included improvements in psychiatric symptoms (i.e., depressive, anxiety, and PTSD symptoms, general psychological distress) from baseline to (1) post-intervention, (2) first follow-up, and (3) last follow-up. When a study included multiple instruments for the same psychiatric outcome, only one scale was chosen based on hypothesized frequency of use^99^. Secondary outcomes included improvements in physical distress (i.e., physical symptoms, functional impairment, self-rated ill health, objective physiological dysfunction) from baseline to different timepoints. If studies included multiple treatment/control arms, each eligible comparison was separately considered, with the sample size of the treatment/control arm divided correspondingly to avoid double counting^100^.

### Quantitative synthesis on effectiveness

To statistically account for any baseline differences, we calculated the Hedge’s *g* (0.2 = small, 0.5 = moderate, 0.8 = large) with 95% CI for each outcome based on the *change score* from baseline to post-intervention (or to follow-ups) between the intervention and control groups^101^. Correlations between scores within the same group was set at 0.7^102^. If insufficient baseline data was reported (2 studies, 4.55%), Hedge’s *g* was calculated based on cross-sectional comparison(s) between the intervention and control groups. Group means and standard deviations, if not readily available for quantitative syntheses, were converted from other statistics (Supplementary Note 3). In addition, the *Q* and *I*^2^ (25% = low, 50% = moderate, 75% = high) indices were calculated to indicate the presence and the degree of heterogeneity across results. Analyses with a random-effects approach were performed using Comprehensive Meta-Analysis version 3.0.

The prospective associations between changes in psychiatric symptoms and changes in physical distress were examined in two meta-regressions, one regressing the effect size of physical distress at follow-ups on that of psychiatric symptoms at post-intervention and one regressing the effect size of psychiatric symptoms at follow-ups on that of physical distress at post-intervention.

### Moderator effects

In the subgroup analyses, demographic and medical characteristics of the patients and the characteristics and methodology of the included interventions were investigated with *Q*-tests and meta-regressions: psychiatric symptoms/physical distress, demographics, medical profile, complementary treatments, intervention delivery platform, presence of guidance (i.e., therapeutic input in the form of therapists’ active provision of intervention, feedback, and/or support)^12^, intervention duration/frequency, therapeutic elements (i.e., behavioral modification, cognitive restructuring, problem-solving, psychoeducation, mindfulness; Supplementary Note 4)^82^, assessment schedule, control type, overall risk of bias, attrition rate, and the use of intention-to-treat analysis. To address dependency issues, multiple effect sizes from the same source were averaged in all meta-analytic procedures^103^.

### Certainty of the evidence

Risk of publication bias was assessed using funnel plots and the Egger test of asymmetry^104^. In cases of significant asymmetry, results were statistically adjusted with the trim-and-fill method^105^.

## Supplementary information

Supplementary Material

## Data Availability

W.K.H. has full access to all of the data in the study and takes responsibility for the integrity of the data and the accuracy of the data analysis. All study materials are available from the corresponding author upon reasonable request.
