# Supplementary material for: Internet-based and mobile-based cognitive behavioral therapy for chronic diseases: a systematic review and meta-analysis
Source: NPJ Digit Med. 2023 Apr 28;6:80. doi: 10.1038/s41746-023-00809-8 (PMC10141870; doi:10.1038/s41746-023-00809-8)
Supplement: Supplementary file 1 — Supplementary Material [file 41746_2023_809_MOESM1_ESM.pdf]

Internet-based and mobile-based cognitive behavioral therapy for chronic diseases:

A systematic review and meta-analysis

TABLE OF CONTENTS FOR THE SUPPLEMENTARY INFORMATION DOCUMENT

**SUPPLEMENTARY TABLE 1**	Detailed characteristics of included studies (Psychiatric symptoms & Moderators) (*n*=44 studies).
**SUPPLEMENTARY TABLE 2**	Detailed characteristics of included studies (Physical distress) (*n*=44 studies).
**SUPPLEMENTARY TABLE 3**	Detailed characteristics of included studies (Therapeutic elements) (*n*=44 studies).
**SUPPLEMENTARY TABLE 4**	Additional details on IM-CBT and control groups (*n*=44 studies).
**SUPPLEMENTARY TABLE 5**	Measurement scales.
**SUPPLEMENTARY TABLE 6**	Evaluation of risk of bias in individual studies (*n*=44 studies).
**SUPPLEMENTARY TABLE 7**	Publication bias statistics (*n*=44 studies).
**SUPPLEMENTARY TABLE 8**	Pooled effect sizes of IM-CBT on psychiatric symptoms and physical distress at post-intervention and follow-ups (*n*=58 studies).
**SUPPLEMENTARY TABLE 9**	Moderators of the effectiveness of IM-CBT on psychiatric symptoms and physical distress (*n*=58 studies).
**SUPPLEMENTARY FIGURE 1**	Forest plots of effect sizes for individual studies (*n*=44 studies).
**SUPPLEMENTARY FIGURE 2**	Funnel plots (*n*=44 studies).

**SUPPLEMENTARY FIGURE 3**	Regression results between effect sizes of improvements on psychiatric symptoms and physical distress (*n*=58 studies).
**SUPPLEMENTARY NOTE 1**	Amendments to registered protocol.
**SUPPLEMENTARY NOTE 2**	Detailed search algorithms & criteria for key terminologies.
**SUPPLEMENTARY NOTE 3**	Details on data handling procedures.
**SUPPLEMENTARY NOTE 4**	The therapeutic strategy framework for Internet- and Mobile-Based Cognitive Behavioral Therapy (IM-CBT).
**SUPPLEMENTARY REFERENCES**	Eligible articles included in the systematic review and meta-analysis (44 included studies); Eligible articles included in the systematic review and meta-analysis (14 studies in appendix only); Other references cited within this document.

SUPPLEMENTARY TABLE 1 Detailed characteristics of included studies (Psychiatric symptoms & Moderators) (n=44 studies).

Authors (Year)	Control 1 (*N*)	Control 2 (*N*)	Intervention 1 (*N*)	Intervention 2 (*N*)	*k*	Country	Female (%)	Age (mean)	Age (range)	Condition	Psychiatric symptoms	Timepoints	Intervention delivery platform (main)	Guidance	Intervention duration (session number)	Intervention weeks	Medication received for physical condition(s)	Surgery received for physical condition(s)	Supplement and/or other received for physical condition(s)	Medication received for psychiatric condition(s)	Psychotherapy received for psychiatric condition(s)	Attrition rate at post-intervention	Existence of physical or psychiatric comorbidity	Control group type	Intention to treat analysis
44 included studies																									
Atema et al, 2019	80	—	81	78	12	Netherlands	100.00	47.40	No report	Cancer	Depressive	Post-intervention	Web-based	Guided	Short (6)	6	Yes	Yes	No	No	No	<5%	Yes	Non-active	Yes
											Anxiety	Follow-up 1		Unguided											
											Depressive & Anxiety														
Barroso et al, 2020	14	—	13	—	2	US	37.00	51.20	No report	HIV	Depressive	Post-intervention	Mobile app	Unguided	Short (10)	10	Yes	No	No	Yes	No	5–20%	Yes	Active	Yes
											Anxiety														
Beatty et al, 2016	26	—	25	—	6	Australia	95.00	52.74	30–84	Cancer	PTSD	Post-intervention	Web-based	Unguided	Short (6)	6	Yes	Yes	No	No	No	5–20%	No	Active	Yes
											General psychological distress	Follow-up 1													
												Follow-up 2													
Buhrman et al, 2004	29	—	22	—	4	Sweden	62.75	44.35	No report	Chronic pain	Depressive	Post-intervention	Web-based	Guided	Short (6)	6	No	Yes	Yes	No	No	5–20%	No	Non-active	No
											Anxiety	Follow-up 1													
Buhrman et al, 2011	27	—	23	—	2	Sweden	68.52	43.19	No report	Chronic pain	Depressive	Post-intervention	Web-based	Guided	Short (8)	12	No	No	Yes	No	No	5–20%	No	Non-active	Yes
											Anxiety														
Buhrman et al, 2013	36	—	36	—	2	Sweden	72.22	40.05	No report	Chronic pain	Depressive	Post-intervention	Web-based	Guided	Short (8)	12	No	No	Yes	No	No	>20%	No	Active	Yes
											Anxiety														
Buhrman et al, 2015	24	—	28	—	2	Sweden	84.62	50.73	22–78	Chronic pain	Depressive	Post-intervention	Web-based	Guided	Short (8)	8	No	No	No	No	No	5–20%	Yes	Active	Yes
											Anxiety														
Chambers et al, 2018	84	—	79	—	1	Australia	68.10	57.00	No report	Cancer	General psychological distress	Post-intervention	Web-based	Guided	Short (6)	8	No	No	No	No	No	>20%	No	Active	Yes

Authors (Year)	Control 1 (*N*)	Control 2 (*N*)	Intervention 1 (*N*)	Intervention 2 (*N*)	*k*	Country	Female (%)	Age (mean)	Age (range)	Condition	Psychiatric symptoms	Timepoints	Intervention delivery platform (main)	Guidance	Intervention duration (session number)	Intervention weeks	Medication received for physical condition(s)	Surgery received for physical condition(s)	Supplement and/or other received for physical condition(s)	Medication received for psychiatric condition(s)	Psychotherapy received for psychiatric condition(s)	Attrition rate at post-intervention	Existence of physical or psychiatric comorbidity	Control group type	Intention to treat analysis
Chiauzzi et al, 2010	104	—	95	—	9	US	67.68	46.14	18–79	Chronic pain	Depressive<br>Anxiety<br>General psychological distress	Post-intervention<br>Follow-up 1<br>Follow-up 2	Web-based	Unguided	Short (8)	4	Yes	No	No	No	No	5–20%	No	Active	Yes
Clarke et al, 2019	241	—	232	—	3	Australia	64.32	57.70	No report	Diabetes	Depressive<br>Anxiety<br>General psychological distress	Post-intervention	Web-based	Unguided	Medium/long (12)	12	Yes	No	No	Yes	Yes	5–20%	Yes	Active	Yes
Cooper et al, 2011	12	—	9	—	2	UK	75.00	45.00	31–57	Multiple sclerosis	Depressive	Post-intervention<br>Follow-up 1	Web-based	Unguided	Short (8)	15	No	No	No	No	No	5–20%	Yes	Non-active	Yes
Dear et al, 2013	30	—	30	—	2	Australia	85.48	49.00	20–91	Chronic pain	Depressive<br>Anxiety	Post-intervention	Web-based	Guided	Short (5)	8	No	No	No	Yes	No	<5%	No	Non-active	No
Dear et al, 2015	74	—	274	123	4	Australia	79.62	50.03	19–86	Chronic pain	Depressive<br>Anxiety	Post-intervention	Web-based	Guided<br>Unguided	Short (5)	8	Yes	No	Yes	Yes	No	5–20%	No	Non-active	Yes
Doorley et al, 2021	11	—	8	—	2	US	65.00	69.95	60–87.1	Different chronic diseases	Depressive<br>Anxiety	Post-intervention	Video conference	Guided	Short (8)	8	Yes	No	No	No	No	5–20%	Yes	Active	No
Dowd et al, 2015	27	—	23	—	2	Ireland	90.32	44.53	19–76	Chronic pain	Depressive & Anxiety	Post-intervention<br>Follow-up 1	Web-based	Unguided	Medium/long (12)	6	Yes	No	Yes	No	No	>20%	No	Active	Yes
Ferguson et al, 2016	14	—	22	—	4	US	100.00	54.60	No report	Cancer	Depressive<br>Anxiety	Post-intervention<br>Follow-up 1	Video conference	Guided	Short (8)	8	Yes	No	No	No	No	>20%	No	Active	No

Authors (Year)	Control 1 (*N*)	Control 2 (*N*)	Intervention 1 (*N*)	Intervention 2 (*N*)	*k*	Country	Female (%)	Age (mean)	Age (range)	Condition	Psychiatric symptoms	Timepoints	Intervention delivery platform (main)	Guidance	Intervention duration (session number)	Intervention weeks	Medication received for physical condition(s)	Surgery received for physical condition(s)	Supplement and/or other received for physical condition(s)	Medication received for psychiatric condition(s)	Psychotherapy received for psychiatric condition(s)	Attrition rate at post-intervention	Existence of physical or psychiatric comorbidity	Control group type	Intention to treat analysis
Ferwerda et al, 2017	59	—	46	—	6	Netherlands	63.91	56.35	26–81	Arthritis	Depressive<br>Anxiety	Post-intervention<br>Follow-up 1<br>Follow-up 2<br>Follow-up 3<br>Follow-up 4	Web-based	Guided	Short (4)	26	No	No	No	No	No	5–20%	No	Non-active	Yes
Friesen et al, 2017	27	—	25	—	2	Canada	95.00	47.50	20–67	Chronic pain	Depressive<br>Anxiety	Post-intervention	Web-based	Guided	Short (5)	8	Yes	No	No	No	No	<5%	No	Non-active	Yes
Gasslander et al, 2022	95	—	92	—	8	Sweden	73.26	45.90	16–70	Chronic pain	Depressive<br>Anxiety<br>Depressive & Anxiety<br>PTSD	Post-intervention<br>Follow-up 1	Web-based	Guided	Short (10)	10	Yes	No	No	Yes	No	>20%	Yes	Non-active	Yes
Geirhos et al, 2022	13	—	11	—	4	Germany	73.33	16.13	12–21	Different chronic diseases	Depressive & Anxiety<br>PTSD	Post-intervention<br>Follow-up 1	Web-based	Guided	Short (7)	7	No	No	No	No	No	5–20%	Yes	Non-active	Yes
Glozier et al, 2013	282	—	280	—	2	Australia	61.39	55.44	No report	Cardio-vascular disease	Depressive<br>Anxiety	Post-intervention	Web-based	Unguided	Medium/long (12)	12	No	No	No	No	No	5–20%	Yes	Active	Yes
Ham et al, 2019	21	21	21	—	4	Korea	85.71	44.17	26–65	Cancer	Depressive<br>Anxiety	Post-intervention	Mobile app	Unguided	Medium/long (48)	10	Yes	Yes	No	No	No	>20%	No	Active<br>Non-active	No
Hummel et al, 2017	82	—	69	—	3	Netherlands	100.00	51.10	No report	Cancer	Depressive<br>Anxiety<br>Depressive & Anxiety	Post-intervention	Web-based	Guided	Medium/long (20)	20	Yes	Yes	No	No	No	5–20%	Yes	Non-active	Yes

Authors (Year)	Control 1 (*N*)	Control 2 (*N*)	Intervention 1 (*N*)	Intervention 2 (*N*)	*k*	Country	Female (%)	Age (mean)	Age (range)	Condition	Psychiatric symptoms	Timepoints	Intervention delivery platform (main)	Guidance	Intervention duration (session number)	Intervention weeks	Medication received for physical condition(s)	Surgery received for physical condition(s)	Supplement and/or other received for physical condition(s)	Medication received for psychiatric condition(s)	Psychotherapy received for psychiatric condition(s)	Attrition rate at post-intervention	Existence of physical or psychiatric comorbidity	Control group type	Intention to treat analysis
Johansson et al, 2019	72	—	72	—	1	Sweden	38.19	62.50	26–87	Cardio-vascular disease	Depressive	Post-intervention	Web-based	Guided	Short (7)	9	Yes	No	No	Yes	No	5–20%	Yes	Active	Yes
Lundgren et al, 2016	25	—	25	—	2	Sweden	41.00	62.90	23–80	Arthritis	Depressive<br>Anxiety	Post-intervention	Web-based	Guided	Short (7)	9	Yes	No	No	Yes	No	5–20%	Yes	Active	Yes
Migliorini et al, 2016	25	—	23	—	3	Australia	28.81	49.75	23–70	Different chronic diseases	Depressive<br>Anxiety<br>General psychological distress	Post-intervention	Web-based	Guided	Short (10)	11	No	No	No	No	No	5–20%	Yes	Non-active	Yes
Mourad et al, 2016	8	—	7	—	2	Sweden	40.00	61.45	22–76	Chronic pain	Depressive<br>Anxiety	Post-intervention	Web-based	Guided	Short (4)	4	No	No	No	No	No	5–20%	No	Non-active	Yes
Murphy et al, 2018	56	—	48	—	4	Australia	88.60	53.29	No report	Cancer	Depressive<br>Anxiety<br>Depressive & Anxiety<br>General psychological distress	Post-intervention	Web-based	Guided	Short (8)	16	Yes	Yes	No	Yes	Yes	5–20%	Yes	Non-active	Yes
Newby et al, 2017	46	—	31	—	3	Australia	71.11	46.66	No report	Diabetes	Depressive<br>Anxiety<br>General psychological distress	Post-intervention	Web-based	Guided	Short (6)	10	Yes	No	No	Yes	Yes	5–20%	Yes	Non-active	Yes
O'moore et al, 2018	23	—	42	—	4	Australia	79.71	61.90	50–81	Arthritis	Depressive	Post-intervention	Web-based	Unguided	Short (6)	10	Yes	No	No	Yes	No	5–20%	Yes	Non-active	Yes

Authors (Year)	Control 1 (*N*)	Control 2 (*N*)	Intervention 1 (*N*)	Intervention 2 (*N*)	*k*	Country	Female (%)	Age (mean)	Age (range)	Condition	Psychiatric symptoms	Timepoints	Intervention delivery platform (main)	Guidance	Intervention duration (session number)	Intervention weeks	Medication received for physical condition(s)	Surgery received for physical condition(s)	Supplement and/or other received for physical condition(s)	Medication received for psychiatric condition(s)	Psychotherapy received for psychiatric condition(s)	Attrition rate at post-intervention	Existence of physical or psychiatric comorbidity	Control group type	Intention to treat analysis
											General psychological distress	Follow-up 1													
Palermo et al, 2009	22	—	23	—	1	US	72.90	14.80	11–17	Chronic pain	Depressive	Post-intervention	Web-based	Guided	Short (8)	8	No	No	No	No	No	5–20%	No	Non-active	Yes
Palermo et al, 2016	135	—	134	—	4	US	75.09	14.66	11–17	Chronic pain	Depressive	Post-intervention	Web-based	Guided	Short (8)	9	No	No	No	No	No	<5%	No	Active	No
											Anxiety	Follow-up 1													
Peters et al, 2017	50	—	112	—	2	Netherlands	84.78	48.55	19–83	Chronic pain	Depressive	Post-intervention	Web-based	Guided	Short (8)	12	No	No	No	No	No	>20%	No	Non-active	Yes
											Anxiety														
Shigaki et al, 2013	49	—	44	—	2	US	92.45	49.81	No report	Arthritis	Depressive	Post-intervention	Web-based	Guided	Short (10)	10	Yes	No	No	No	No	5–20%	No	Non-active	No
												Follow-up 1													
Simblett et al, 2017	8	—	15	—	4	UK	35.71	62.90	No report	Cardio-vascular disease	Depressive	Post-intervention	Web-based	Guided	Short (8)	8	No	No	No	No	No	5–20%	Yes	Active	No
											Anxiety	Follow-up 1													
Stinson et al, 2010	24	—	22	—	1	Canada	67.39	14.61	No report	Arthritis	General psychological distress	Post-intervention	Web-based	Guided	Medium/long (12)	12	Yes	No	No	No	No	5–20%	No	Active	Yes
Taguchi et al, 2021	14	—	13	—	2	Japan	65.52	47.06	22–75	Chronic pain	Depressive	Post-intervention	Video conference	Guided	Medium/long (16)	16	No	No	No	No	No	5–20%	Yes	Non-active	No
											Anxiety														
Thesen et al, 2022	69	—	64	—	6	Norway	54.04	52.00	20–69	Chronic pain	Depressive	Post-intervention	Web-based	Guided	Short (6)	6	No	No	No	No	No	5–20%	Yes	Non-active	Yes
											Anxiety	Follow-up 1													
												Follow-up 2													
Trautmann and Kröner-Herwig, 2010	20	18	17	—	4	Germany	55.38	12.65	No report	Chronic pain	Depressive	Post-intervention	Web-based	Guided	Short (6)	6	No	No	No	No	No	5–20%	No	Active	Yes

Authors (Year)	Control 1 (*N*)	Control 2 (*N*)	Intervention 1 (*N*)	Intervention 2 (*N*)	*k*	Country	Female (%)	Age (mean)	Age (range)	Condition	Psychiatric symptoms	Timepoints	Intervention delivery platform (main)	Guidance	Intervention duration (session number)	Intervention weeks	Medication received for physical condition(s)	Surgery received for physical condition(s)	Supplement and/or other received for physical condition(s)	Medication received for psychiatric condition(s)	Psychotherapy received for psychiatric condition(s)	Attrition rate at post-intervention	Existence of physical or psychiatric comorbidity	Control group type	Intention to treat analysis
												Follow-up 1													
Trudeau et al, 2015	115	—	113	—	9	US	68.42	49.95	No report	Arthritis	Depressive	Post-intervention	Web-based	Unguided	Short (8)	4	No	No	No	No	No	<5%	No	Non-active	Yes
											Anxiety	Follow-up 1													
											General psychological distress	Follow-up 2													
Westas et al, 2022	35	—	65	—	3	Sweden	38.19	62.90	No report	Cardio-vascular disease	Depressive	Post-intervention	Web-based	Guided	Short (7)	9	No	No	No	Yes	No	5–20%	Yes	Active	No
												Follow-up 1													
												Follow-up 2													
Wiklund et al, 2022	27	—	20	—	4	Sweden	83.33	49.27	21–67	Chronic pain	Depressive	Post-intervention	Web-based	Guided	Short (5)	5	Yes	No	No	Yes	No	5–20%	Yes	Active	Yes
											Anxiety	Follow-up 1													
Williams et al, 2010	59	—	59	—	2	US	94.92	50.46	No report	Chronic pain	Depressive	Post-intervention	Web-based	Unguided	Medium/long (13)	13	Yes	No	No	No	No	5–20%	Yes	Non-active	Yes
											Anxiety														
Wilson et al, 2018	25	—	22	—	1	US	85.11	46.66	No report	Different chronic diseases	Depressive	Post-intervention	Web-based	Unguided	Medium/long (14)	14	No	No	No	No	No	5–20%	Yes	Active	No
14 studies in appendix only																									
Andreae et al, 2021	87	—	89	—	8	US	78.53	58.92	No report	Different chronic diseases	Depressive	Post-intervention	Telephone	Guided	Short (8)	12	No	No	No	No	No	5–20%	Yes	Active	Yes
											General psychological distress	Follow-up 1													
Carmody et al, 2013	51	—	50	—	3	US	3.10	67.53	No report	Chronic pain	Depressive	Post-intervention	Telephone	Guided	Medium/long (12)	20	No	No	No	No	No	>20%	Yes	Active	Yes
												Follow-up 1													

Authors (Year)	Control 1 (*N*)	Control 2 (*N*)	Intervention 1 (*N*)	Intervention 2 (*N*)	*k*	Country	Female (%)	Age (mean)	Age (range)	Condition	Psychiatric symptoms	Timepoints	Intervention delivery platform (main)	Guidance	Intervention duration (session number)	Intervention weeks	Medication received for physical condition(s)	Surgery received for physical condition(s)	Supplement and/or other received for physical condition(s)	Medication received for psychiatric condition(s)	Psychotherapy received for psychiatric condition(s)	Attrition rate at post-intervention	Existence of physical or psychiatric comorbidity	Control group type	Intention to treat analysis
												Follow-up 2													
Casault et al, 2015	18	—	17	—	6	Canada	92.10	56.90	33–75	Cancer	Depressive	Post-intervention	Self-help materials	Guided	Short (6)	6	Yes	No	No	No	No	5–20%	Yes	Non-active	Yes
											Anxiety	Follow-up 1													
												Follow-up 2													
Doyle et al, 2017	56	—	54	—	4	Australia	65.45	67.74	No report	COPD	Depressive	Post-intervention	Telephone	Guided	Short (8)	8	No	No	Yes	Yes	Yes	5-20%	Yes	Active	Yes
											Anxiety	Follow-up 1													
Eller, 1995	12	—	23	22	2	US	13.04	36.40	22–76	HIV	Depressive	Post-intervention	Self-help materials	Unguided	Medium/long (24)	13	Yes	No	No	No	No	5–20%	No	Non-active	No
Howard and Dupont, 2014	68	—	71	—	4	UK	57.66	72.19	No report	COPD	Depressive	Post-intervention	Self-help materials	Guided	Short (5)	6	No	No	No	Yes	No	>20%	Yes	Active	Yes
											Anxiety	Follow-up 1													
Kelleher et al, 2021	16	—	14	—	2	US	38.71	59.50	No report	Cancer	General psychological distress	Post-intervention	Telephone	Guided	Short (5)	6	No	No	No	No	No	<5%	Yes	Non-active	No
												Follow-up 1													
Kraaij et al, 2010	15	16	13	—	2	Netherlands	11.36	49.48	No report	HIV	Depressive	Post-intervention	Self-help materials	Unguided	Medium/long (16)	4	Yes	No	No	No	No	5–20%	No	Active<br>Non-active	No
McAndrew et al, 2018	31	—	32	—	4	US	5.50	56.59	No report	Different chronic diseases	Depressive	Post-intervention	Telephone	Guided	Short (10)	12	No	No	No	No	No	5–20%	No	Non-active	Yes
											PTSD	Follow-up 1													
McCurry et al, 2021	146	—	136	—	2	US	74.62	70.25	No report	Arthritis	Depressive	Post-intervention	Telephone	Guided	Short (6)	8	Yes	No	No	Yes	No	5–20%	Yes	Active	Yes
												Follow-up 1													
Mohr et al, 2000	16	—	16	—	1	US	71.90	42.35	No report	Multiple sclerosis	Depressive	Post-intervention	Telephone	Guided	Short (8)	8	No	No	No	Yes	Yes	>20%	Yes	Non-active	Yes

Authors (Year)	Control 1 (*N*)	Control 2 (*N*)	Intervention 1 (*N*)	Intervention 2 (*N*)	*k*	Country	Female (%)	Age (mean)	Age (range)	Condition	Psychiatric symptoms	Timepoints	Intervention delivery platform (main)	Guidance	Intervention duration (session number)	Intervention weeks	Medication received for physical condition(s)	Surgery received for physical condition(s)	Supplement and/or other received for physical condition(s)	Medication received for psychiatric condition(s)	Psychotherapy received for psychiatric condition(s)	Attrition rate at post-intervention	Existence of physical or psychiatric comorbidity	Control group type	Intention to treat analysis
Rawlings et al, 2022	27	—	30	—	4	UK	94.74	47.84	No report	Hyper-tension	Depression<br>Anxiety	Post-intervention<br>Follow-up 1	Self-help materials	Unguided	Short (4)	4	No	No	No	Yes	Yes	>20%	No	Non-active	No
Savard et al, 2014	81	—	80	—	2	Canada	100.00	54.43	No report	Cancer	Depression<br>Anxiety	Post-intervention	Self-help materials	Unguided	Short (6)	6	Yes	Yes	No	Yes	No	5–20%	Yes	Non-active	Yes
Stefanopoulou et al, 2015	27	—	30	—	4	UK	0	68.87	49–83	Cancer	Depressive<br>Anxiety	Post-intervention<br>Follow-up 1	Self-help materials	Guided	Short (4)	4	Yes	Yes	Yes	No	No	<5%	No	Non-active	Yes

Abbreviations. COPD=Chronic obstructive pulmonary disease.

Definitions. "Guidance" was defined as: "Guided" refers to therapists' therapeutic input, including active provision of intervention, feedback, and/or support, whereas "Unguided" refers to technical/adherence or other non-specified assistance only (Karyotaki, 2021) (for further details, see Supplementary Table 4); "Intervention duration" was defined as: <12 sessions=short, 12–16 sessions=medium, >16 sessions=long (van Beugen, 2014); "Attrition rate at post-intervention" was defined as: <5%=low bias, 5–20%=moderate bias, and >20%=high bias (Schulz and Grimes, 2002); "Non-active" control group included waitlist control (WLC) and treatment-as-usual (TAU) / standard care (SC), whereas "active" control group included information/education (*k*=10), discussion forum (*k*=5), relaxation (*k*=2), attention control (scheduled contact) (*k*=2), supportive therapy (*k*=1), computerized cognitive remediation therapy (*k*=1), and lifestyle management (*k*=1) (for further details, see Supplementary Table 4); "general psychological distress" included distress (e.g., Kessler 10-item Psychological Distress Scale [K-10]) and stress (e.g., "Depression Anxiety Stress Scale-21 (DASS-21) Stress Subscale").

Notes. *k*=number of effect sizes; *N*=total sample size. Special handling of intervention and control arms included: (1) Two intervention arms, divided control *N* in half (Atema et al, 2019; Eller, 1995); (2) Three intervention arms, merged guided intervention arms, control N divided into 2:1 (guided:unguided) ratio (Dear et al, 2015); (3) Two control arms, divided intervention *N* in half (Ham et al, 2019; Trautmann and Kröner-Herwig, 2010; Kraaij et al, 2010).

SUPPLEMENTARY TABLE 2 Detailed characteristics of included studies (Physical distress) (*n*=44 studies).

Authors (Year)	Control 1 (*N*)	Control 2 (*N*)	Intervention 1 (*N*)	Intervention 2 (*N*)	*k*	Condition	Physical distress	Timepoints
44 included studies								
Atema et al, 2019	80	—	82	80	28	Cancer	Perceived impact of HF/NS	Post-intervention
							Overall levels of menopausal symptoms	Follow-up 1
							Sleep quality	
							HF frequency	
							NS frequency	
							Physical functioning	
							Bodily pain	
Barroso et al, 2020	14	—	13	—	4	HIV	HRFS fatigue intensity	Post-intervention
							HRFS overall fatigue-related functioning	Follow-up 1
							Fatigue	
							HIV viral load	
							CD4 count	
Beatty et al, 2016	26	—	25	—	0	—	—	—
Buhrman et al, 2004	29	—	22	—	4	Chronic pain	Pain severity	Post-intervention
							Average pain intensity	Follow-up 1
Buhrman et al, 2011	27	—	23	—	1	Chronic pain	Pain severity	Post-intervention
Buhrman et al, 2013	36	—	36	—	1	Chronic pain	Pain severity	Post-intervention

Authors (Year)	Control 1 (*N*)	Control 2 (*N*)	Intervention 1 (*N*)	Intervention 2 (*N*)	*k*	Condition	Physical distress	Timepoints
Buhrman et al, 2015	24	—	28	—	2	Chronic pain	Pain disability	Post-intervention
							Pain severity	
Chambers et al, 2018	84	—	79	—	0	—	—	—
Chiauzzi et al, 2010	104	—	95	—	3	Chronic pain	Disability	Post-intervention
								Follow-up 1
								Follow-up 2
Clarke et al, 2019	241	—	232	—	0	—	—	—
Cooper et al, 2011	12	—	9	—	2	Multiple sclerosis	Physical impact	Post-intervention
								Follow-up 1
Dear et al, 2013	30	—	30	—	2	Chronic pain	Disability	Post-intervention
							Average pain	
Dear et al, 2015	74	—	274	123	4	Chronic pain	Disability	Post-intervention
							Average pain	
Doorley et al, 2021	11	—	8	—	4	Different chronic diseases	Physical function	Post-intervention
							Disability	
							Pain at rest	
							Pain with activity	
Dowd et al, 2015	27	—	23	—	4	Chronic pain	Average pain	Post-intervention
							Pain now	Follow-up 1

Authors (Year)	Control 1 (*N*)	Control 2 (*N*)	Intervention 1 (*N*)	Intervention 2 (*N*)	*k*	Condition	Physical distress	Timepoints
Ferguson et al, 2016	14	—	22	—	2	Cancer	Fatigue	Post-intervention
								Follow-up 1
Ferwerda et al, 2017	59	—	46	—	6	Arthritis	Pain	Post-intervention
							Fatigue	Follow-up 1
								Follow-up 2
Friesen et al, 2017	27	—	25	—	4	Chronic pain	Fibromyalgia severity and symptomology	Post-intervention
							Fatigue	
							Pain	
							Physical health	
Gasslander et al, 2022	95	—	92	—	6	Chronic pain	Pain disability	Post-intervention
							Insomnia severity	Follow-up 1
							Pain severity	
Geirhos et al, 2022	13	—	11	—	0	—	—	—
Glozier et al, 2013	282	—	280	—	1	Cardiovascular disease	Disability	Post-intervention
Ham et al, 2019	21	21	21	—	0	—	—	—
Hummel et al, 2017	82	—	69	—	4	Cancer	Overall sexual functioning	Post-intervention
							Menopausal symptoms	
							Physical functioning	
							Bodily pain	

Authors (Year)	Control 1 (N)	Control 2 (N)	Intervention 1 (N)	Intervention 2 (N)	*k*	Condition	Physical distress	Timepoints
Johansson et al, 2019	72	—	72	—	0	—	—	—
Lundgren et al, 2016	25	—	25	—	0	—	—	—
Migliorini et al, 2016	25	—	23	—	0	—	—	—
Mourad et al, 2016	8	—	7	—	1	Chronic pain	Body sensations	Post-intervention
Murphy et al, 2018	56	—	48	—	0	—	—	—
Newby et al, 2017	46	—	31	—	2	Diabetes	Haemoglobin A1c	Post-intervention
							Physical well-being	
O'moore et al, 2018	23	—	42	—	6	Arthritis	OA-specific pain	Post-intervention
							OA-specific stiffness	Follow-up 1
							OA-specific physical function	
Palermo et al, 2009	23	—	22	—	2	Chronic pain	Pain intensity	Post-intervention
							Pain over one month	
Palermo et al, 2016	135	—	134	—	4	Chronic pain	Pain intensity	Post-intervention
							Sleep quality	Follow-up 1
Peters et al, 2017	50	—	112	—	2	Chronic pain	Physical impairment	Post-intervention
							Pain intensity	
Shigaki et al, 2013	49	—	44	—	6	Arthritis	Arthritis symptoms	Post-intervention
							Overall health (physical)	Follow-up 1
Simblett et al, 2017	8	—	15	—	0	—	—	—

Authors (Year)	Control 1 (*N*)	Control 2 (*N*)	Intervention 1 (*N*)	Intervention 2 (*N*)	*k*	Condition	Physical distress	Timepoints
Stinson et al, 2010	24	—	22	—	3	Arthritis	Gross motor function	Post-intervention
							Fine motor function	
							General symptoms	
Taguchi et al, 2021	14	—	13	—	3	Chronic pain	Pain intensity (composite)	Post-intervention
							Pain severity	
							Pain disability	
Thesen et al, 2022	69	—	64	—	3	Chronic pain	Body sensations	Post-intervention
								Follow-up 1
								Follow-up 2
Trautmann and Kröner-Herwig, 2010	20	17	16	—	8	Chronic pain	Headache frequency	Post-intervention
							Headache intensity	Follow-up 1
Trudeau et al, 2015	115	—	113	—	9	Arthritis	Pain severity	Post-intervention
							Physical impact	Follow-up 1
							Arthritis symptoms	Follow-up 2
Westas et al, 2022	35	—	65	—	0	—	—	—
Wiklund et al, 2022	27	—	20	—	4	Chronic pain	Pain intensity	Post-intervention
							Pain disability	Follow-up 1
Williams et al, 2010	59	—	59	—	3	Chronic pain	Pain severity	Post-intervention
							Physical functioning	

Authors (Year)	Control 1 (*N*)	Control 2 (*N*)	Intervention 1 (*N*)	Intervention 2 (*N*)	*k*	Condition	Physical distress	Timepoints
							Fatigue	
Wilson et al, 2018	25	—	22	—	1	Different chronic diseases	General health	Post-intervention
14 studies in appendix only								
Andreae et al, 2021	87	—	89	—	0	—	—	—
Carmody et al, 2013	51	—	50	—	6	Chronic pain	Physical health	Post-intervention
							Pain intensity	Follow-up 1
								Follow-up 2
Casault et al, 2015	18	—	17	—	6	Cancer	Fatigue	Post-intervention
							Insomnia severity	Follow-up 1
								Follow-up 2
Doyle et al, 2017	56	—	54	—	0	—	—	—
Eller, 1995	24	—	23	22	6	HIV	CD4+ mm3 lymphocyte count	Post-intervention
							CD16+ mm3 lymphocyte count	
							Fatigue	
Howard and Dupont, 2014	68	—	71	—	4	COPD	Dyspnoea	Post-intervention
							Fatigue	Follow-up 1
Kelleher et al, 2021	16	—	14	—	2	Cancer	Pain severity	Post-intervention
								Follow-up 1
Kraaij et al, 2010	15	16	13	—	0	—	—	—

Authors (Year)	Control 1 (*N*)	Control 2 (*N*)	Intervention 1 (*N*)	Intervention 2 (*N*)	*k*	Condition	Physical distress	Timepoints
McAndrew et al, 2018	31	—	32	—	4	Different chronic diseases	Physical function	Post-intervention
							Physical symptoms	Follow-up 1
McCurry et al, 2021	146	—	136	—	6	Arthritis	Insomnia	Post-intervention
							Pain severity	Follow-up 1
							Fatigue	
Mohr et al, 2000	16	—	16	—	0	—	—	—
Rawlings et al, 2022	27	—	30	—	2	Hypertension	Dyspnoea	Post-intervention
								Follow-up 1
Savard et al, 2014	81	—	80	—	0	—	—	—
Stefanopoulou et al, 2015	34	—	32	—	7	Cancer	HFNS total frequency	Post-intervention
							HF frequency	Follow-up 1
							NS frequency	
							Sternal Skin Conductance	

Abbreviations. COPD=Chronic obstructive pulmonary disease; HF=Hot flushes; HFNS=Hot flushes and night sweats; HRFS=HIV-Related Fatigue Scale; NS=Night sweats; OA=Osteoarthritis.

Notes. *k*=number of effect sizes; *N*=total sample size. Special handling of intervention and control arms included: (1) Two intervention arms, divided control *N* in half (Atema et al, 2019; Eller, 1995); (2) Three intervention arms, merged guided intervention arms, control N divided into 2:1 (guided:unguided) ratio (Dear et al, 2015); (3) Two control arms, divided intervention *N* in half (Ham et al, 2019; Trautmann and Kröner-Herwig, 2010; Kraaij et al, 2010).

SUPPLEMENTARY TABLE 3 Detailed characteristics of included studies (Therapeutic elements) (*n*=44 studies).

Authors (Year)	Behavioral modification	Cognitive restructuring	Problem solving	Psychoeducation	Mindfulness	Total number of elements
14 included studies						
Atema et al, 2019	Yes	Yes	Yes	Yes	Yes	5
Barroso et al, 2020	Yes		Yes	Yes	Yes	4
Beatty et al, 2016	Yes		Yes	Yes	Yes	4
Buhrman et al, 2004	Yes	Yes	Yes	Yes		4
Buhrman et al, 2011	Yes	Yes	Yes	Yes	Yes	5
Buhrman et al, 2013	Yes	Yes	Yes	Yes	Yes	5
Buhrman et al, 2015	Yes		Yes	Yes	Yes	4
Chambers et al, 2018	Yes		Yes	Yes	Yes	4
Chiauzzi et al, 2010	Yes		Yes		Yes	3
Clarke et al, 2019		Yes	Yes	Yes	Yes	4
Cooper et al, 2011	Yes	Yes	Yes	Yes		4
Dear et al, 2013	Yes	Yes	Yes	Yes	Yes	5
Dear et al, 2015	Yes	Yes	Yes	Yes	Yes	5
Doorley et al, 2021	Yes		Yes	Yes	Yes	4
Dowd et al, 2015	Yes	Yes	Yes	Yes	Yes	5
Ferguson et al, 2016	Yes	Yes	Yes	Yes		4
Ferwerda et al, 2017	Yes	Yes	Yes			3
Friesen et al, 2017	Yes	Yes	Yes	Yes	Yes	5

Gasslander et al, 2022	Yes		Yes	Yes	Yes	4
Geirhos et al, 2022	Yes	Yes	Yes	Yes	Yes	5
Glozier et al, 2013	Yes	Yes	Yes	Yes	Yes	5
Ham et al, 2019	Yes	Yes	Yes	Yes		4
Hummel et al, 2017	Yes	Yes	Yes	Yes	Yes	5
Johansson et al, 2019	Yes		Yes	Yes		3
Lundgren et al, 2016	Yes		Yes	Yes		3
Migliorini et al, 2016	Yes	Yes	Yes		Yes	4
Mourad et al, 2016	Yes		Yes	Yes		3
Murphy et al, 2018	Yes	Yes	Yes	Yes	Yes	5
Newby et al, 2017	Yes	Yes	Yes	Yes	Yes	5
O’moore et al, 2018	Yes	Yes	Yes			3
Palermo et al, 2009	Yes	Yes				2
Palermo et al, 2016	Yes	Yes	Yes	Yes		4
Peters et al, 2017	Yes	Yes	Yes	Yes	Yes	5
Shigaki et al, 2013	Yes		Yes	Yes	Yes	4
Simblett et al, 2017	Yes	Yes	Yes			3
Stinson et al, 2010	Yes	Yes	Yes	Yes	Yes	5
Taguchi et al, 2021	Yes	Yes	Yes	Yes	Yes	5
Thesen et al, 2022	Yes		Yes	Yes		3
Trautmann and Kröner-Herwig, 2010	Yes	Yes	Yes	Yes	Yes	5
Trudeau et al, 2015	Yes	Yes	Yes	Yes	Yes	5

Westas et al, 2022	Yes		Yes	Yes	Yes	4
Wiklund et al, 2022	Yes		Yes	Yes		3
Williams et al, 2010	Yes	Yes	Yes	Yes		4
Wilson et al, 2018	Yes	Yes	Yes			3
14 studies in appendix only						
Andreae et al, 2021	Yes	Yes	Yes	Yes	Yes	5
Carmody et al, 2013	Yes	Yes	Yes		Yes	4
Casault et al, 2015	Yes	Yes	Yes			3
Doyle et al, 2017	Yes	Yes				2
Eller, 1995	Yes	Yes	Yes		Yes	4
Howard and Dupont, 2014	Yes	Yes	Yes	Yes		4
Kelleher et al, 2021	Yes	Yes	Yes		Yes	4
Kraaij et al, 2010	Yes		Yes		Yes	3
McAndrew et al, 2018		Yes	Yes	Yes	Yes	4
McCurry et al, 2021	Yes	Yes	Yes	Yes	Yes	5
Mohr et al, 2000	Yes	Yes	Yes	Yes	Yes	5
Rawlings et al, 2022	Yes		Yes	Yes	Yes	4
Savard et al, 2014	Yes	Yes	Yes	Yes		4
Stefanopoulou et al, 2015	Yes	Yes	Yes	Yes		4

Notes. Special handling of intervention and control arms included: (1) Two intervention arms, divided control *N* in half (Atema et al, 2019; Eller, 1995); (2) Three intervention arms, merged guided intervention arms, control N divided into 2:1 (guided:unguided) ratio (Dear et al, 2015); (3) Two control arms, divided intervention *N* in half (Ham et al, 2019; Trautmann and Kröner-Herwig, 2010; Kraaij et al, 2010).

SUPPLEMENTARY TABLE 4 Additional details on IM-CBT and control groups (*n*=44 studies).

**Authors (Year)**	**IM-CBT details**											**Control details**	
	**Delivery platform**			**Additional materials**	**Mode of communication**	**Adherence information (definition)**	**Therapist involvement**					**Active control specifics**	**Non-active control specifics**
	**Video-conference**	**Web-based**	**Mobile app**				**Provision of intervention**	**Provision of feedback on homework**	**Provision of support**	**Performance of logistic roles**	**No report**		
44 included studies													
Atema et al, 2019		✓			Email;<br>Online message;<br>Telephone	Completion of all modules +<br>Completion of all homework		✓<br>(*For guided intervention group only*)	✓<br>(*For guided intervention group only*)	✓			WLC
Barroso et al, 2020			✓		In-person (*Data collection*);<br>Telephone	Completion of all modules				✓		Information / Education	
Beatty et al, 2016		✓		Worksheets;<br>Quizzes;<br>Exercises;<br>Journal	Email;<br>Telephone	Amount of modules completed;<br>Amount of platform usage				✓		Information / Education	
Buhrman et al, 2004		✓		CD	Email;<br>Telephone	No report		✓	✓	✓			WLC
Buhrman et al, 2011		✓			Email;<br>Telephone	Completion of all modules +<br>Completion of all homework		✓	✓	✓			WLC
Buhrman et al, 2013		✓			Email	No report		✓	✓			Discussion forum	
Buhrman et al, 2015		✓			Email;<br>Telephone;<br>Automated text message	Completion of all modules		✓	✓	✓		Discussion forum	
Chambers et al, 2018		✓			Email	Patients' self-report on an adherence questionnaire		✓		✓		Information / Education	
Chiauzzi et al, 2010		✓		Self-help guide (soft-copy)	Email	Fulfillment of a minimal amount of module usage				✓		Information / Education	
Clarke et al, 2019		✓		SMS message	Text message;<br>Automated email	Completion of a minimal proportion of modules +<br>Completion of a minimal proportion of homework +<br>Instant provision of post-intervention and follow-up data				✓		Lifestyle management	

Authors (Year)	IM-CBT details											Control details	
	Delivery platform			Additional materials	Mode of communication	Adherence information (definition)	Therapist involvement					Active control specifics	Non-active control specifics
	Video-conference	Web-based	Mobile app				Provision of intervention	Provision of feedback on homework	Provision of support	Performance of logistic roles	No report		
Cooper et al, 2011		✓			No report	No report					✓		WLC
Dear et al, 2013		✓			Email;<br>Telephone;<br>Automated email	Completion of all modules + Provision of post-intervention and follow-up data			✓	✓			WLC
Dear et al, 2015		✓			Email;<br>Telephone;<br>Automated email	Amount of modules completed			✓<br>(*For guided intervention group only*)	✓			WLC
Doorley et al, 2021	✓			Accelerometer;<br>Fitbit;<br>Mobile app (*Data collection*)	Videoconference	Completion of a minimal proportion of homework	✓		✓			Information / Education	
Dowd et al, 2015		✓			Email	Amount of homework completed				✓		Information / Education	
Ferguson et al, 2016	✓			Workbook	Videoconference	No report	✓					Supportive therapy	
Ferwerda et al, 2017		✓			In-person (*Patient intake*)<br>Email	Completion of a minimal proportion of modules		✓	✓				TAU/SC
Friesen et al, 2017		✓			Online message;<br>Telephone;<br>Automated email	Completion of all modules;<br>Instant provision of post-intervention and follow-up data			✓	✓			WLC
Gasslander et al, 2022		✓			Online message;<br>Telephone	Completion of a minimal proportion of modules		✓	✓	✓			WLC
Geirhos et al, 2022		✓<br>(*Main*)	✓		Online message;<br>Telephone	Completion of a minimal proportion of modules + Completion of all homework		✓	✓	✓			WLC
Glozier et al, 2013		✓			Email;<br>Telephone;<br>Text;<br>Automated email	Completion of all modules				✓		Information / Education	

Authors (Year)	IM-CBT details											Control details	
	Delivery platform			Additional materials	Mode of communication	Adherence information (definition)	Therapist involvement					Active control specifics	Non-active control specifics
	Video-conference	Web-based	Mobile app				Provision of intervention	Provision of feedback on homework	Provision of support	Performance of logistic roles	No report		
Ham et al, 2019			✓		Telephone; Text	Routine access to the modules (no long periods of inactivity)				✓		Information / Education	WLC
Hummel et al, 2017		✓			Email; Telephone	Completion of all modules		✓					WLC
Johansson et al, 2019		✓			Email; Online message	Amount of modules completed; Amount of homework completed		✓	✓	✓		Discussion forum	
Lundgren et al, 2016		✓			Email	Completion of all modules		✓		✓		Discussion forum	
Migliorini et al, 2016		✓			Email; Telephone	Completion of all modules + Completion of all homework			✓				WLC
Mourad et al, 2016		✓			Email; Telephone; Text	Completion of all modules + Completion of all homework		✓	✓				TAU/SC
Murphy et al, 2018		✓			Email; Telephone; Automated email	Routine access to the modules (no long periods of inactivity)			✓				TAU/SC
Newby et al, 2017		✓			Email; Telephone; Automated email	Completion of all modules			✓	✓			TAU/SC
O'moore et al, 2018		✓			Email; Telephone	Completion of all modules				✓			TAU/SC
Palermo et al, 2009		✓			Online message	Completion of all modules + Completion of all homework		✓	✓				WLC
Palermo et al, 2016		✓			Online message	Completion of all modules + Completion of a minimal proportion of modules		✓	✓			Information / Education	
Peters et al, 2017		✓		Workbook	Email; Telephone	Completion of all modules + Completion of all homework			✓				WLC
Shigaki et al, 2013		✓			Telephone	No report			✓				WLC

Authors (Year)	IM-CBT details											Control details	
	Delivery platform			Additional materials	Mode of communication	Adherence information (definition)	Therapist involvement					Active control specifics	Non-active control specifics
	Video-conference	Web-based	Mobile app				Provision of intervention	Provision of feedback on homework	Provision of support	Performance of logistic roles	No report		
Simblett et al, 2017		✓			In-person (*Supervision*)<br>Email;<br>Telephone	Successful delivery of intended intervention			✓			Computerized cognitive remediation therapy	
Stinson et al, 2010		✓			Telephone	Patients' self-report on an adherence questionnaire		✓	✓			Attention control (scheduled contact)	
Taguchi et al, 2021	✓				Videoconference	No report	✓						TAU/SC
Thesen et al, 2022		✓			Telephone	Completion of all modules + Completion of all homework			✓				TAU/SC
Trautmann and Kröner-Herwig, 2010		✓		CD	Email;<br>Telephone	No report		✓		✓		(*Control 1*) Information / Education;<br>(*Control 2*) Relaxation	
Trudeau et al, 2015		✓			Email;<br>Telephone	Completion of all modules				✓			WLC
Westas et al, 2022		✓			No report	Amount of modules completed		✓				Discussion forum	
Wiklund et al, 2022		✓		SMS message;<br>PowerPoints	Online message;<br>Telephone	Completion of all modules + Completion of all homework		✓	✓	✓		Relaxation	
Williams et al, 2010		✓			Email;<br>Telephone	Amount of homework completed				✓			TAU/SC
Wilson et al, 2018		✓			Email;<br>Telephone	No report				✓		Attention control (scheduled contact)	
14 studies in appendix only													
Andreae et al, 2021				Telephone;<br>Booklet;<br>DVD;<br>Health calendar	Telephone	Completion of all modules + Provision of post-intervention and follow-up data	✓					Information / Education	

Authors (Year)	IM-CBT details											Control details	
	Delivery platform			Additional materials	Mode of communication	Adherence information (definition)	Therapist involvement					Active control specifics	Non-active control specifics
	Video-conference	Web-based	Mobile app				Provision of intervention	Provision of feedback on homework	Provision of support	Performance of logistic roles	No report		
Carmody et al, 2013				Telephone; Handouts	Telephone	Patients' feedback on perceived helpfulness	✓	✓	✓			Information / Education	
Casault et al, 2015				Booklet	Telephone	Completion of all modules + Completion of all homework			✓				TAU/SC
Doyle et al, 2017				Telephone	Telephone	No report	✓					Befriending	
Eller, 1995				Audiotape	In-person (*Instructions provision*); Telephone	Amount of modules used; Frequency of modules used				✓			TAU/SC
Howard and Dupont, 2014				Telephone; Booklet; CD	Telephone	Completion of a minimal proportion of homework	✓ (*Booster session only*)					Information / Education	
Kelleher et al, 2021				Telephone; Pamphlet; Audiotape	Telephone	Completion of a minimal proportion of modules + Provision of post-intervention and follow-up data	✓		✓				TAU/SC
Kraaij et al, 2010				Workbook; Work program; CD	No report	No report					✓	Computerized structured writing intervention	WLC
McAndrew et al, 2018				Telephone	Telephone	No report	✓						TAU/SC
McCurry et al, 2021				Telephone; Booklet	Telephone	Completion of all modules	✓					Information / Education	
Mohr et al, 2000				Telephone; Workbook	Telephone	No report	✓						TAU/SC
Rawlings et al, 2022				Booklet	Email; Telephone	Patients' feedback on perceived helpfulness					✓		WLC
Savard et al, 2014				Booklet; DVD	Telephone	Completion of all modules + Completion of a minimal proportion of homework							TAU/SC
Stefanopoulou et al, 2015				Booklet; CD	Telephone	Amount of modules completed;			✓				TAU/SC

Authors (Year)	IM-CBT details											Control details	
	Delivery platform			Additional materials	Mode of communication	Adherence information (definition)	Therapist involvement					Active control specifics	Non-active control specifics
	Video-conference	Web-based	Mobile app				Provision of intervention	Provision of feedback on homework	Provision of support	Performance of logistic roles	No report		
						Amount of homework completed							

Abbreviations. SC=Standard care; TAU=Treatment as usual; WLC=Wait-list control.

Notes. Therapist involvement only took into consideration the provision of intervention, feedback, and support that was pre-planned at part of the intervention protocol; support provided only upon request was not included. Special handling of intervention and control arms included: (1) Two intervention arms, divided control *N* in half (Atema et al, 2019; Eller, 1995); (2) Three intervention arms, merged guided intervention arms, control N divided into 2:1 (guided:unguided) ratio (Dear et al, 2015); (3) Two control arms, divided intervention *N* in half (Ham et al, 2019; Trautmann and Kröner-Herwig, 2010; Kraaij et al, 2010).

SUPPLEMENTARY TABLE 5 Measurement scales.

Measurement scales for psychiatric symptoms

Outcome	Scale
Depressive symptoms	Bath Adolescent Pain Questionnaire (BAPQ)-depression scale, Beck Depression Inventory (BDI), Beck Depression Inventory-II (BDI-II), Center for Epidemiologic Studies-Depression scale (CES-D), Child Depression Inventory (CDI), Depression Anxiety Stress Scales-21 (DASS-21), Hamilton Depression Rating Scale (HDRS), Hospital Anxiety and Depression Scale (HADS), Montgomery-Asberg Depression Rating Scale (MADRS), Patient Health Questionnaire-9 (PHQ-9), Patient-Reported Outcomes Measurement (PROMIS)-depression scale, The Revised Child Anxiety and Depression Scale (RCADS)-major depressive disorder scale
Anxiety symptoms	Bath Adolescent Pain Questionnaire (BAPQ)-pain-specific anxiety scale, Beck Anxiety Inventory (BAI), Cardiac Anxiety Questionnaire (CAQ), Depression Anxiety Stress Scales-21 (DASS-21), Hospital Anxiety and Depression Scale (HADS), Impact of Rheumatic Diseases on General Health and Lifestyle (IRGL)-anxiety scale, Patient-Reported Outcomes Measurement (PROMIS)-anxiety scale, State-Trait Anxiety Inventory-Trait (STAI-Trait), State-Trait Personality Inventory-State (STPI-State)-anxiety scale
Depressive and anxiety symptoms	Depression Anxiety Stress Scales-21 (DASS-21), Hospital Anxiety and Depression Scale (HADS), Patient Health Questionnaire Anxiety and Depression Scale (PHQ-ADS)
Posttraumatic stress disorder symptoms	Child and Adolescent Trauma Screen (CATS), Posttraumatic Stress Scale-Self Report (PSS-SR)

General psychological distress	Brief Symptom Inventory-18 Global Severity Index (BSI-18), Diabetes Distress Scale (DDS), Kessler 10-item Psychological Distress Scale (K-10), Perceived Severity of Stress Questionnaire (PSQ)

Notes. The following scales were present only in the 14 studies included in appendix only (not listed above): *Depressive symptoms* The Profile of Mood States (PMS)-depression-dejection scale; *PTSD symptoms* PTSD Checklist-Civilian Version (PCL-C); *General psychological distress* Perceived Stress Scale (PSS10).

Measurement scales for physical distress

**Outcome**	**Scale**
Physical symptoms	36-Item Short Form Survey (SF-36)-bodily pain scale, Arthritis Impact Measurement Scale (AIMS), Arthritis Impact Measurement Scales 2 (AIMS2), Body Sensations Questionnaire (BSQ), Brief Pain Inventory (BPI), Brief Pain Inventory (BPI)-severity, Checklist Individual Strength (CIS)-fatigue scale, Fatigue Symptom Inventory (FSI), Functional Assessment of Cancer Therapy-Endocrine Symptoms (FACT-ES), Functional Assessment of Chronic Illness Therapy-Fatigue (FACIT-F), HIV-Related Fatigue Scale (HRFS), Headache diary, Hospital Anxiety and Depression Scale-Anxiety (HADS-A), Hot Flush Rating Scale (HFRS)-hot flush frequency, Hot Flush Rating Scale (HFRS)-night sweats frequency, Impact of Rheumatic Diseases on General Health and Lifestyle (IRGL)-pain scale, Insomnia Severity Index (ISI), Juvenile Arthritis Quality of Life Questionnaire (JAQQ), Multidimensional Fatigue Inventory (MFI), Multidimensional Pain Inventory (MPI), Multiple Sclerosis Impact Scale (MSIS-29), Numerical Rating Scale (NRS), Patient Reported Outcomes Measurement Information System Fatigue-Short Form (PROMIS F-SF), Rapid Assessment of Disease Activity in Rheumatology (RADAR), The Revised Fibromyalgia Impact Questionnaire (FIQR), WHO Disability Assessment

	Schedule (WHODAS II), Western Ontario and McMaster Universities Osteoarthritis Index (WOMAC), Wisconsin Brief Pain Questionnaire (WBPQ)
Functional impairment	36-Item Short Form Survey (SF-36), Adolescent Sleep Wake Scale (ASWS), Female Sexual Function Index (FSFI), Fibromyalgia Impact Questionnaire (FIQ), Functional Assessment of Cancer Therapy-General (FACT-G), Groningen Sleep Quality Scale (GSQS), Hot Flush Rating Scale (HFRS)-problem rating, Juvenile Arthritis Quality of Life Questionnaire (JAQQ), Oswestry Disability Questionnaire (ODQ), Pain Disability Assessment Scale (PDAS), Pain Disability Index (PDI), Patient Reported Outcomes Measurement Information System (PROMIS), Roland Morris Disability Questionnaire (RMDQ), SF-36 physical functioning, Western Ontario and McMaster Universities Osteoarthritis Index (WOMAC)
Self-rated ill health	Arthritis Impact Measurement Scales 2 (AIMS2), Self-Rated Health Scale, Short Form 12-item Physical Health Subscale (SF-12 PCS)
Objective physiological dysfunction	HbA1c test, Patient medical record

Notes. The following measures were present only in the 14 studies included in appendix only (not listed above): *Physical symptoms* Brief Pain Inventory-short form (BPI-sf)-severity, Dyspnoea 12 (D12), Flinders Fatigue Scale (FFS), Hot Flush Rating Scale (HFRS), Pain Catastrophizing Scale (PCS), Self-monitoring diary, Sickness Impact Profile (SIP)-Rest and Sleep subscale, The Self-Reported Chronic Respiratory Questionnaire (CRQ-SR); *Functional impairment* Veterans RAND 36-Item Health Survey (VR-36); *Self-rated ill health* Short Form 12v2 Health Survey (SF-12v2); *Objective physiological dysfunction* Participant blood sample, Sternal Skin Conductance (SSC) monitor.

SUPPLEMENTARY TABLE 6 Evaluation of risk of bias in individual studies (*n*=44 studies).

Study	Randomization	Assignment	Adherence	Missing data	Outcome measurement	Selective reporting	Overall risk
44 included studies							
Atema et al, 2019	L	L	L	L	H	L	H
Barroso et al, 2020	S	S	L	L	S	S	S
Beatty et al, 2016	L	S	S	L	L	L	S
Buhrman et al, 2004	L	H	H	H	L	S	H
Buhrman et al, 2011	L	S	S	L	H	S	H
Buhrman et al, 2013	L	S	S	L	H	S	H
Buhrman et al, 2015	L	S	S	L	S	S	S
Chambers et al, 2018	L	L	S	L	L	L	S
Chiauzzi et al, 2010	L	S	S	L	L	S	S
Clarke et al, 2019	S	S	S	S	S	L	S
Cooper et al, 2011	S	S	S	L	S	S	S
Dear et al, 2013	L	S	H	L	L	L	H
Dear et al, 2015	L	S	S	L	S	S	S
Doorley et al, 2021	S	S	S	L	L	L	S
Dowd et al, 2015	L	L	L	L	S	S	S
Ferguson et al, 2016	L	S	S	S	S	S	S

Study	Randomization	Assignment	Adherence	Missing data	Outcome measurement	Selective reporting	Overall risk
Ferwerda et al, 2017	L	L	L	L	L	L	L
Friesen et al, 2017	L	S	S	L	L	S	S
Gasslander et al, 2022	L	L	H	S	L	L	H
Geirhos et al, 2022	S	L	H	L	L	L	H
Glozier et al, 2013	L	L	L	L	L	L	L
Ham et al, 2019	L	S	S	H	S	S	H
Hummel et al, 2017	L	S	L	L	S	S	S
Johansson et al, 2019	L	L	L	L	S	L	S
Lundgren et al, 2016	L	L	S	L	S	L	S
Migliorini et al, 2016	L	L	H	L	S	L	H
Mourad et al, 2016	L	S	L	L	S	S	S
Murphy et al, 2018	L	L	S	L	S	L	S
Newby et al, 2017	L	L	S	L	S	L	S
O'moore et al, 2018	L	S	S	L	L	S	S
Palermo et al, 2009	L	S	S	L	S	S	S
Palermo et al, 2016	L	L	L	L	L	L	L
Peters et al, 2017	L	S	H	L	S	S	H
Shigaki et al, 2013	S	L	S	L	S	L	S

Study	Randomization	Assignment	Adherence	Missing data	Outcome measurement	Selective reporting	Overall risk
Simblett et al, 2017	S	S	L	S	S	S	S
Stinson et al, 2010	L	L	H	L	S	L	H
Taguchi et al, 2021	S	S	S	L	S	L	S
Thesen et al, 2022	L	L	H	L	L	L	H
Trautmann and Kröner-Herwig, 2010	S	L	H	L	S	L	H
Trudeau et al, 2015	L	L	H	L	S	L	H
Westas et al, 2022	S	S	H	L	S	L	H
Wiklund et al, 2022	L	L	H	S	S	L	H
Williams et al, 2010	L	L	H	S	L	S	H
Wilson et al, 2018	S	S	S	L	S	S	S
14 studies in appendix only							
Andreae et al, 2021	S	L	H	L	S	L	H
Carmody et al, 2013	L	L	S	L	L	L	S
Casault et al, 2015	L	H	H	H	S	S	H
Doyle et al, 2017	L	L	S	L	L	L	S
Eller, 1995	L	S	S	L	S	S	S
Howard and Dupont, 2014	L	L	S	H	L	S	H
Kelleher et al, 2021	L	S	H	L	S	S	H

Study	Randomization	Assignment	Adherence	Missing data	Outcome measurement	Selective reporting	Overall risk
Kraaij et al, 2010	L	S	L	S	S	S	S
McAndrew et al, 2018	L	L	S	H	L	L	H
McCurry et al, 2021	S	L	L	L	L	L	S
Mohr et al, 2000	L	S	S	L	S	S	S
Rawlings et al, 2022	L	S	H	S	L	L	H
Savard et al, 2014	L	L	L	L	L	L	L
Stefanopoulou et al, 2015	L	S	H	S	S	S	H

Abbreviations. L=low risk of bias, S=some concerns, H=high risk of bias.

SUPPLEMENTARY TABLE 7 Publication bias statistics (*n*=44 studies).

Timepoint	Outcome domain	Outcome	*k*	Classic fail-safe *N*	Observed Hedge's *g* (95% CI)	Adjusted Hedge's *g* (95% CI)	Studies trimmed *N*	Egger's regression intercept
**Post-intervention**	**Psychiatric**	**Depressive symptoms**	43	1757	**0.448 (0.309 to 0.587)**	**0.448 (0.309 to 0.587)**	0	1.162 (–0.375 to 2.700)
		**Anxiety symptoms**	34	619	**0.322 (0.193 to 0.451)**	**0.322 (0.193 to 0.451)**	0	0.706 (–0.804 to 2.217)
		Depressive and anxiety symptoms	7	44	0.447 (–0.029 to 0.922)	0.447 (–0.029 to 0.922)	0	2.412 (–9.124 to 13.947)
		Posttraumatic stress disorder symptoms	3	19	1.083 (–0.266 to 2.432)	1.083 (–0.266 to 2.432)	0	8.593 (–104.754 to 121.940)
		**General psychological distress**	10	165	**0.623 (0.229 to 1.016)**	**0.623 (0.229 to 1.016)**	0	5.882 (1.260 to 10.504)
	**Physical**	**Physical symptoms**	31	152	**0.184 (0.106 to 0.263)**	**0.155 (0.063 to 0.246)**	3	0.555 (–0.533 to 1.644)
		**Functional impairment**	21	234	**0.284 (0.178 to 0.390)**	**0.237 (0.121 to 0.352)**	3	1.569 (0.316 to 2.822)
		Self-rated ill health	4	0	0.080 (–0.279 to 0.483)	–0.052 (–0.433 to 0.329)	1	–2.180 (–28.967 to 24.606)
		Objective physiological dysfunction	2	0	—	—	—	—
**First follow-up**	**Psychiatric**	**Depressive symptoms**	18	139	**0.319 (0.142 to 0.497)**	**0.319 (0.142 to 0.497)**	0	0.476 (–1.603 to 2.555)
		**Anxiety symptoms**	12	9	**0.171 (0.020 to 0.322)**	**0.171 (0.020 to 0.322)**	0	–0.988 (–3.061 to 1.084)
		**Depressive and anxiety symptoms**	5	4	**0.241 (0.020 to 0.461)**	0.192 (–0.077 to 0.462)	1	2.849 (–1.138 to 6.836)
		**Posttraumatic stress disorder symptoms**	3	11	**0.867 (0.453 to 1.282)**	**0.867 (0.453 to 1.282)**	0	1.012 (–19.042 to 21.067)
		**General psychological distress**	4	31	**0.581 (0.195 to 0.968)**	0.336 (–0.045 to 0.718)	2	4.446 (–5.354 to 14.247)
	**Physical**	Physical symptoms	15	0	0.047 (–0.147 to 0.241)	0.047 (–0.147 to 0.241)	0	–0.963 (–3.415 to 1.489)
		Functional impairment	9	8	0.182 (–0.039 to 0.403)	0.182 (–0.039 to 0.403)	0	0.776 (–3.057 to 4.608)
		Self-rated ill health	1	—	0.000 (–0.414 to 0.414)	—	—	—
		Objective physiological dysfunction	0	—	—	—	—	—
**Last follow-up**	**Psychiatric**	**Depressive symptoms**	5	24	**0.357 (0.207 to 0.507)**	**0.316 (0.154 to 0.479)**	1	1.288 (–4.758 to 7.335)
		**Anxiety symptoms**	4	13	**0.321 (0.162 to 0.481)**	**0.321 (0.162 to 0.481)**	0	0.667 (–4.840 to 6.175)
		Depressive and anxiety symptoms	0	—	—	—	—	—

	**Posttraumatic stress disorder symptoms**	1	—	**0.576 (0.024 to 1.128)**	—	—	—
	**General psychological distress**	3	26	**0.673 (0.180 to 1.165)**	**0.673 (0.180 to 1.165)**	0	6.394 (–3.378 to 16.167)
**Physical**	Physical symptoms	3	9	0.191 (–0.003 to 0.384)	0.191 (–0.003 to 0.384)	0	–0.988 (–13.869 to 11.893)
	Functional impairment	2	—	–0.016 (–0.205 to 0.174)	—	—	—
	Self-rated ill health	0	—	—	—	—	—
	Objective physiological dysfunction	0	—	—	—	—	—

Notes. Bold texts indicate significant results. Results of publication bias analyses for outcomes with few comparisons (k<10) should be interpreted with caution. For certain variables, publication bias analyses were not conducted because there were fewer than three comparison in the original analysis (i.e., insufficient for analyses).

SUPPLEMENTARY TABLE 8 Pooled effect sizes of IM-CBT on psychiatric symptoms and physical distress at post-intervention and follow-ups (*n*=58 studies).

Timepoint	Outcome domain	Outcome	*k*	Pooled *g* (95% CI)	*p*	I^2 (%)	*Q*
**Post-intervention**	**Psychiatric**	**Depressive symptoms**	**58**	**0.429 (0.316 to 0.541)**	**<0.001**	**75.005**	**228.046**
		**Anxiety symptoms**	**40**	**0.299 (0.184 to 0.414)**	**<0.001**	**68.512**	**123.855**
		Depressive and anxiety symptoms	7	0.447 (–0.029 to 0.922)	0.066	89.455	56.846
		Posttraumatic stress disorder (PTSD) symptoms	4	0.828 (–0.120 to 1.776)	0.087	90.220	30.675
		**General psychological distress**	**12**	**0.503 (0.156 to 0.849)**	**0.004**	**90.754**	**118.967**
	**Physical**	**Physical symptoms**	**41**	**0.239 (0.112 to 0.365)**	**<0.001**	**67.733**	**123.967**
		**Functional impairment**	**22**	**0.278 (0.176 to 0.380)**	**<0.001**	**35.559**	**32.588**
		Self-rated ill health	5	–0.404 (–0.397 to 0.316)	0.824	66.016	11.770
		Objective physiological dysfunction	5	–0.036 (–0.312 to 0.239)	0.796	0	2.556
**First follow-up**	**Psychiatric**	**Depressive symptoms**	**27**	**0.295 (0.163 to 0.427)**	**<0.001**	**59.401**	**64.040**
		**Anxiety symptoms**	**17**	**0.198 (0.054 to 0.343)**	**0.007**	**47.051**	**30.197**
		**Depressive and anxiety symptoms**	**5**	**0.241 (0.020 to 0.461)**	**0.032**	**5.266**	**4.222**
		Posttraumatic stress disorder (PTSD) symptoms	4	0.587 (–0.006 to 1.181)	0.053	68.365	9.483
		**General psychological distress**	**6**	**0.384 (0.041 to 0.727)**	**0.028**	**78.677**	**23.449**
	**Physical**	Physical symptoms	23	0.097 (–0.076 to 0.270)	0.273	70.777	75.283
		Functional impairment	10	0.175 (–0.026 to 0.377)	0.088	60.622	22.855
		Self-rated ill health	2	–0.128 (–0.412 to 0.155)	0.375	0	0.693

		Objective physiological dysfunction	0	—	—	—	—
**Last follow-up**	**Psychiatric**	**Depressive symptoms**	**7**	**0.293 (0.138 to 0.448)**	**<0.001**	**17.430**	**7.267**
		**Anxiety symptoms**	**5**	**0.338 (0.183 to 0.493)**	**<0.001**	**0**	**1.485**
		Depressive and anxiety symptoms	0	—	—	—	—
		**Posttraumatic stress disorder (PTSD) symptoms**	**1**	**0.576 (0.024 to 1.128)**	**0.041**	**0**	**0**
		**General psychological distress**	**3**	**0.673 (0.180 to 1.165)**	**0.007**	**83.308**	**11.982**
	**Physical**	**Physical symptoms**	**5**	**0.178 (0.011 to 0.346)**	**0.037**	**0**	**2.452**
		Functional impairment	2	–0.016 (–0.205 to 0.174)	0.872	0	0.124
		Self-rated ill health	1	0.000 (–0.387 to 0.387)	>0.999	0	0
		Objective physiological dysfunction	0	—	—	—	—

Definitions. "Physical symptoms" includes: Arthritis symptoms, General symptoms, Physical symptoms, Menopausal symptoms, Osteoarthritis (OA)-specific stiffness, Dyspnoea, Fatigue, Insomnia, HIV-Related Fatigue Scale (HRFS) Fatigue intensity, Average pain, Pain at rest, Bodily pain, Pain, Pain intensity, Pain now, Pain severity, Pain with activity, Osteoarthritis (OA)-specific pain, Headache intensity, Bodily sensations, Headache frequency, Hot flush (HF) frequency, HIV-Related Fatigue Scale (HRFS) total frequency, Night sweats (NS) frequency; "Functional impairment" includes: Disability, Fine motor function, Gross motor function, Functional well-being, Gross motor function, Osteoarthritis (OA)-specific physical function, Overall sexual functioning, Physical function, HIV-Related Fatigue Scale (HRFS) overall fatigue-related functioning, Pain disability, Physical impairment, Physical impact, Sleep quality; "Self-rated ill health" includes: General health, Physical health, Overall health (physical), Physical well-being; "Objective physiological dysfunction" includes: Haemoglobin A1c, HIV viral load, CD4 count, Sternal skin conductance.

Notes. *k*=Number of averaged effect sizes (to address the potential dependency issues, when multiple effect sizes were available from the same source, the moderator analyses were done based on the averaged effect sizes). Bold texts indicate significant results.

SUPPLEMENTARY TABLE 9 Moderators of the effectiveness of IM-CBT on psychiatric symptoms and physical distress (*n*=58 studies).

Moderator	Psychiatric symptoms				Physical distress			
	k	**Statistic type**	**Statistic value (95% CI)**	***p***	***k***	**Statistic type**	**Statistic value (95% CI)**	***p***
**Model 1**								
**Psychiatric symptoms**								
**Subgroup differences**	—	*Q*-value	4.023	0.403	—	—	—	—
Depressive symptoms	58	Hedge's *g*	0.433 (0.320 to 0.545)	<0.001	—	—	—	—
Anxiety symptoms	40	Hedge's *g*	0.312 (0.197 to 0.427)	<0.001	—	—	—	—
Depressive and anxiety symptoms	7	Hedge's *g*	0.489 (–0.023 to 1.001)	0.061	—	—	—	—
PTSD symptoms	4	Hedge's *g*	0.639 (0.035 to 1.243)	0.038	—	—	—	—
General psychological distress	12	Hedge's *g*	0.559 (0.211 to 0.908)	0.002	—	—	—	—
**Model 2**								
**Physical distress**								
**Subgroup differences**	—	—	—	—	—	*Q*-value	6.379	0.095
Physical symptoms	—	—	—	—	41	Hedge's *g*	0.204 (0.087 to 0.322)	0.001
Functional impairment	—	—	—	—	22	Hedge's *g*	0.275 (0.162 to 0.389)	<0.001
Self-rated ill health	—	—	—	—	5	Hedge's *g*	–0.006 (–0.302 to 0.290)	0.967
Objective physiological dysfunction	—	—	—	—	5	Hedge's *g*	–0.036 (–0.312 to 0.239)	0.796
**Model 3**								
**Gender**								

Female percentage (0%–100%)	64	Coefficient	0.003 (–0.002 to 0.007)	0.218	46	Coefficient	0.003 (–0.002 to 0.007)	0.228
**Model 4**								
**Chronic disease**								
**Subgroup differences**	—	*Q*-value	6.196	0.720	—	*Q*-value	43.517	<0.001
Chronic pain	22	Hedge's *g*	0.369 (0.198 to 0.540)	<0.001	22	Hedge's *g*	0.215 (0.097 to 0.333)	<0.001
Cancer	13	Hedge's *g*	0.318 (0.008 to 0.627)	0.044	7	Hedge's *g*	0.125 (–0.097 to 0.347)	0.270
Arthritis	7	Hedge's *g*	0.347 (0.105 to 0.589)	0.005	6	Hedge's *g*	0.268 (0.030 to 0.507)	0.028
Cardiovascular disease	4	Hedge's *g*	0.504 (0.245 to 0.764)	<0.001	1	Hedge's *g*	0.072 (–0.093 to 0.238)	0.391
Diabetes	2	Hedge's *g*	0.461 (–0.580 to 1.503)	0.385	1	Hedge's *g*	0.024 (–0.427 to 0.475)	0.916
Multiple sclerosis	2	Hedge's *g*	1.000 (0.395 to 1.606)	0.001	1	Hedge's *g*	0.278 (–0.587 to 1.142)	0.529
HIV	5	Hedge's *g*	0.353 (0.012 to 0.695)	0.042	3	Hedge's *g*	0.075 (–0.325 to 0.475)	0.712
COPD	2	Hedge's *g*	0.290 (–0.243 to 0.823)	0.286	1	Hedge's *g*	1.522 (1.097 to 1.946)	<0.001
Hypertension	1	Hedge's *g*	0.664 (0.136 to 1.192)	0.014	1	Hedge's *g*	–0.005 (–0.518 to 0.508)	0.984
Different chronic diseases	6	Hedge's *g*	0.418 (0.215 to 0.621)	<0.001	3	Hedge's *g*	–0.122 (–0.635 to 0.390)	0.640
**Model 5**								
**Existence of physical or psychiatric comorbidity**								
**Subgroup differences**	—	*Q*-value	0.195	0.658	—	*Q*-value	0.689	0.407
Yes	33	Hedge's *g*	0.370 (0.224 to 0.517)	<0.001	21	Hedge's *g*	0.249 (0.071 to 0.428)	0.006
No	31	Hedge's *g*	0.415 (0.282 to 0.547)	<0.001	25	Hedge's *g*	0.162 (0.059 to 0.265)	0.002

**Model 6**								
**Medication received for physical condition(s)**								
**Subgroup differences**	—	*Q*-value	0.121	0.728	—	*Q*-value	0.134	0.714
Yes	33	Hedge's *g*	0.406 (0.241 to 0.571)	<0.001	23	Hedge's *g*	0.228 (0.117 to 0.339)	<0.001
No	31	Hedge's *g*	0.370 (0.255 to 0.486)	<0.001	23	Hedge's *g*	0.190 (0.021 to 0.359)	0.027
**Model 7**								
**Surgery received for physical condition(s)**								
**Subgroup differences**	—	*Q*-value	0.072	0.789	—	*Q*-value	0.581	0.446
Yes	10	Hedge's *g*	0.435 (0.066 to 0.805)	0.021	5	Hedge's *g*	0.113 (–0.131 to 0.357)	0.362
No	54	Hedge's *g*	0.383 (0.282 to 0.484)	<0.001	41	Hedge's *g*	0.218 (0.107 to 0.328)	<0.001
**Model 8**								
**Supplement and/or other received for physical condition(s)**								
**Subgroup differences**	—	*Q*-value	0.013	0.910	—	*Q*-value	0.001	0.978
Yes	8	Hedge's *g*	0.369 (0.034 to 0.704)	0.031	7	Hedge's *g*	0.202 (–0.031 to 0.436)	0.089
No	56	Hedge's *g*	0.389 (0.283 to 0.495)	<0.001	39	Hedge's *g*	0.206 (0.093 to 0.319)	<0.001
**Model 9**								

**Medication received for psychiatric condition(s)**								
**Subgroup differences**	—	*Q*-value	4.079	0.043	—	*Q*-value	3.859	0.049
Yes	19	Hedge's *g*	0.565 (0.324 to 0.806)	<0.001	11	Hedge's *g*	0.409 (0.131 to 0.686)	0.004
No	45	Hedge's *g*	0.300 (0.210 to 0.390)	<0.001	35	Hedge's *g*	0.121 (0.046 to 0.196)	0.002
**Model 10**<br>**Psychotherapy (non-CBT) received for psychiatric condition(s)**								
**Subgroup differences**	—	*Q*-value	1.533	0.216	—	*Q*-value	1.239	0.266
Yes	6	Hedge's *g*	0.751 (0.131 to 1.372)	0.018	2	Hedge's *g*	0.011 (–0.327 to 0.350)	0.947
No	58	Hedge's *g*	0.355 (0.263 to 0.447)	<0.001	44	Hedge's *g*	0.213 (0.108 to 0.318)	<0.001
**Model 11**<br>**Main intervention delivery format**								
**Subgroup differences**	—	*Q*-value	6.718	0.152	—	*Q*-value	1.183	0.881
Videoconference	3	Hedge's *g*	–0.013 (–0.445 to 0.419)	0.953	3	Hedge's *g*	0.254 (–0.340 to 0.847)	0.402
Web-based	42	Hedge's *g*	0.448 (0.321 to 0.576)	<0.001	32	Hedge's *g*	0.180 (0.093 to 0.267)	<0.001
Mobile app	3	Hedge's *g*	0.403 (–0.272 to 1.077)	0.242	1	Hedge's *g*	0.154 (–0.546 to 0.855)	0.666
Telephone	7	Hedge's *g*	0.157 (–0.124 to 0.438)	0.273	4	Hedge's *g*	–0.015 (–0.515 to 0.484)	0.952
Self-help materials	9	Hedge's *g*	0.351 (0.165 to 0.538)	<0.001	6	Hedge's *g*	0.397 (–0.187 to 0.981)	0.182
**Model 12**								

**Guidance**								
**Subgroup differences**	—	*Q*-value	0.057	0.811	—	*Q*-value	0.928	0.335
Guided	43	Hedge's *g*	0.376 (0.249 to 0.504)	<0.001	34	Hedge's *g*	0.225 (0.093 to 0.358)	0.001
Unguided	21	Hedge's *g*	0.402 (0.237 to 0.567)	<0.001	12	Hedge's *g*	0.134 (0.003 to 0.265)	0.045
**Model 13**								
**Intervention length (no. of sessions)**								
**Subgroup differences**	—	*Q*-value	5.132	0.023	—	*Q*-value	4.352	0.037
Medium/long (≥12 sessions)	15	Hedge's *g*	0.206 (0.061 to 0.352)	0.005	10	Hedge's *g*	0.067 (–0.048 to 0.182)	0.251
Short (<12 sessions)	49	Hedge's *g*	0.425 (0.305 to 0.545)	<0.001	36	Hedge's *g*	0.246 (0.124 to 0.368)	<0.001
**Model 14**								
**Intervention duration (weeks)**								
Duration in weeks (4–26 weeks)	64	Coefficient	0.003 (–0.019 to 0.026)	0.771	46	Coefficient	–0.002 (–0.022 to 0.018)	0.830
**Model 15**								
**Intervention session number**								
Session number (4–48)	64	Coefficient	0.001 (–0.013 to 0.016)	0.852	46	Coefficient	–0.021 (–0.044 to 0.001)	0.063
**Model 16**								
**Intervention frequency**								
Sessions per week (0.15–4.80)	64	Coefficient	0.002 (–0.133 to 0.137)	0.976	46	Coefficient	–0.274 (–0.504 to –0.044)	0.020
**Model 17**								
**Total number of therapeutic elements**								

Number of components (2–5)	64	Coefficient	0.075 (–0.050 to 0.200)	0.241	46	Coefficient	0.037 (–0.093 to 0.167)	0.574
**Model 18**								
**Intervention has behavioral modification element**								
**Subgroup differences**	—	*Q*-value	18.738	<0.001	—	*Q*-value	2.224	0.136
Yes	62	Hedge's *g*	0.404 (0.303 to 0.506)	<0.001	45	Hedge's *g*	0.212 (0.110 to 0.315)	<0.001
No	2	Hedge's *g*	–0.031 (–0.200 to 0.138)	0.721	1	Hedge's *g*	–0.170 (–0.662 to 0.322)	0.499
**Model 19**								
**Intervention has cognitive restructuring element**								
**Subgroup differences**	—	*Q*-value	0.005	0.945	—	*Q*-value	2.343	0.126
Yes	48	Hedge's *g*	0.392 (0.270 to 0.514)	<0.001	37	Hedge's *g*	0.230 (0.112 to 0.348)	<0.001
No	16	Hedge's *g*	0.385 (0.231 to 0.539)	<0.001	9	Hedge's *g*	0.078 (–0.075 to 0.232)	0.318
**Model 20**								
**Intervention has problem-solving element**								
**Subgroup differences**	—	*Q*-value	4.888	0.027	—	*Q*-value	3.382	0.066
Yes	62	Hedge's *g*	0.401 (0.298 to 0.504)	<0.001	45	Hedge's *g*	0.194 (0.093 to 0.296)	<0.001
No	2	Hedge's *g*	0.029 (–0.283 to 0.342)	0.854	1	Hedge's *g*	0.765 (0.165 to 1.366)	0.012
**Model 21**								

**Intervention has psychoeducation element**								
**Subgroup differences**	—	*Q*-value	0.024	0.878	—	*Q*-value	0.767	0.381
Yes	48	Hedge's *g*	0.385 (0.271 to 0.498)	<0.001	36	Hedge's *g*	0.229 (0.118 to 0.340)	<0.001
No	16	Hedge's *g*	0.405 (0.176 to 0.633)	0.001	10	Hedge's *g*	0.107 (–0.143 to 0.357)	0.402
**Model 22** **Intervention has mindfulness element**								
**Subgroup differences**	—	*Q*-value	1.262	0.261	—	*Q*-value	0.130	0.719
Yes	42	Hedge's *g*	0.427 (0.297 to 0.557)	<0.001	31	Hedge's *g*	0.181 (0.092 to 0.271)	<0.001
No	22	Hedge's *g*	0.311 (0.155 to 0.466)	<0.001	15	Hedge's *g*	0.233 (–0.035 to 0.502)	0.088
**Model 23** **Measurement timepoint**								
**Subgroup differences**	—	*Q*-value	1.695	0.428	—	*Q*-value	2.705	0.259
Last follow-up	8	Hedge's *g*	0.375 (0.204 to 0.546)	<0.001	6	Hedge's *g*	0.097 (–0.046 to 0.241)	0.183
First follow-up	31	Hedge's *g*	0.277 (0.155 to 0.398)	<0.001	25	Hedge's *g*	0.116 (–0.038 to 0.270)	0.140
Post-intervention	64	Hedge's *g*	0.377 (0.274 to 0.479)	<0.001	46	Hedge's *g*	0.233 (0.123 to 0.343)	<0.001
**Model 24** **Follow-up duration after intervention (weeks)**								
Duration in weeks (4–48 weeks)	39	Coefficient	–0.003 (–0.012 to 0.005)	0.450	31	Coefficient	0.000 (–0.011 to 0.011)	0.999

**Model 25**								
**Control group type**								
**Subgroup differences**	—	*Q*-value	3.598	0.058	—	*Q*-value	0.013	0.909
Active	28	Hedge's *g*	0.285 (0.174 to 0.396)	<0.001	16	Hedge's *g*	0.192 (–0.037 to 0.422)	0.101
Non-active	36	Hedge's *g*	0.475 (0.313 to 0.636)	<0.001	30	Hedge's *g*	0.207 (0.110 to 0.304)	<0.001
**Model 26**								
**Overall risk of bias**								
**Subgroup differences**	—	*Q*-value	5.199	0.074	—	*Q*-value	0.097	0.953
High risk	26	Hedge's *g*	0.292 (0.154 to 0.430)	<0.001	21	Hedge's *g*	0.186 (0.007 to 0.364)	0.042
Some concerns	34	Hedge's *g*	0.496 (0.328 to 0.664)	<0.001	21	Hedge's *g*	0.212 (0.065 to 0.360)	0.005
Low risk	4	Hedge's *g*	0.264 (0.143 to 0.384)	<0.001	4	Hedge's *g*	0.177 (–0.013 to 0.367)	0.068
**Model 27**								
**Attrition rate at post-intervention**								
**Subgroup differences**	—	*Q*-value	2.297	0.317	—	*Q*-value	0.093	0.954
High (>20%)	12	Hedge's *g*	0.464 (0.233 to 0.695)	<0.001	8	Hedge's *g*	0.202 (–0.200 to 0.603)	0.325
Moderate (5–20%)	44	Hedge's *g*	0.401 (0.274 to 0.529)	<0.001	30	Hedge's *g*	0.193 (0.084 to 0.302)	0.001
Low (<5%)	8	Hedge's *g*	0.232 (0.008 to 0.456)	0.043	8	Hedge's *g*	0.228 (0.032 to 0.424)	0.023
**Model 28**								
**Intention-to-treat analysis**								
**Subgroup differences**	—	*Q*-value	0.053	0.818	—	*Q*-value	2.997	0.083

Yes	46	Hedge's *g*	0.395 (0.276 to 0.513)	<0.001	34	Hedge's *g*	0.253 (0.141 to 0.365)	<0.001
No	18	Hedge's *g*	0.369 (0.180 to 0.557)	<0.001	12	Hedge's *g*	0.028 (–0.201 to 0.257)	0.812

Abbreviations. COPD=Chronic obstructive pulmonary disease.

Notes. *k*=Number of averaged effect sizes (to address the potential dependency issues, when multiple effect sizes were available from the same source, the moderator analyses were done based on the averaged effect sizes). The moderator analysis for "Follow-up duration after intervention" was only analyzed upon follow-up data points.

Definitions. "Guidance" was defined as: "Guided" refers to therapists' therapeutic input, including active provision of intervention, feedback, and/or support, whereas "Unguided" refers to technical/adherence or other non-specified assistance only (Karyotaki, 2021). "Intervention duration" was defined as: <12 sessions=short, 12–16 sessions=medium, and >16 sessions=long (van Beugen, 2014). "Non-active" control group included waitlist control (WLC) and treatment-as-usual (TAU) / standard care (SC); "Active" control group included information/education (*k*=10), discussion forum (*k*=5), relaxation (*k*=2), attention control (scheduled contact) (*k*=2), supportive therapy (*k*=1), computerized cognitive remediation therapy (*k*=1), and lifestyle management (*k*=1). "Attrition rate at post-intervention" was defined as: <5%=low bias, 5–20%=moderate bias, and >20%=high bias (Schulz and Grimes, 2002).

SUPPLEMENTARY FIGURE 1 Forest plots of effect sizes for individual studies (*n*=44 studies).

Depressive symptoms (Post-intervention)

Study name	Condition	Outcome	Time point	Statistics for each study: Hedges's g	Standard error	Variance	Lower limit	Upper limit	Z-Value	p-Value
Atema et al, 2019a	Cancer	Depressive symptoms	Post-intervention	0.241	0.193	0.037	-0.137	0.618	1.250	0.211
Atema et al, 2019b	Cancer	Depressive symptoms	Post-intervention	0.066	0.193	0.037	-0.313	0.445	0.341	0.733
Barroso et al, 2020	HIV	Depressive symptoms	Post-intervention	0.212	0.356	0.127	-0.487	0.910	0.594	0.552
Buhrman et al, 2004	Chronic pain	Depressive symptoms	Post-intervention	-0.087	0.279	0.078	-0.633	0.459	-0.314	0.754
Buhrman et al, 2011	Chronic pain	Depressive symptoms	Post-intervention	0.395	0.282	0.080	-0.158	0.947	1.399	0.162
Buhrman et al, 2013	Chronic pain	Depressive symptoms	Post-intervention	0.443	0.236	0.056	-0.019	0.906	1.878	0.060
Buhrman et al, 2015	Chronic pain	Depressive symptoms	Post-intervention	0.820	0.286	0.082	0.260	1.380	2.872	0.004
Chiauzzi et al, 2010	Chronic pain	Depressive symptoms	Post-intervention	0.107	0.141	0.020	-0.171	0.384	0.753	0.451
Clarke et al, 2019	Diabetes	Depressive symptoms	Post-intervention	-0.000	0.092	0.008	-0.180	0.180	-0.000	1.000
Cooper et al, 2011	Multiple sclerosis	Depressive symptoms	Post-intervention	0.779	0.440	0.194	-0.084	1.641	1.770	0.077
Dear et al, 2013	Chronic pain	Depressive symptoms	Post-intervention	0.762	0.264	0.070	0.244	1.279	2.883	0.004
Dear et al, 2015a	Chronic pain	Depressive symptoms	Post-intervention	1.219	0.162	0.026	0.902	1.537	7.525	0.000
Dear et al, 2015b	Chronic pain	Depressive symptoms	Post-intervention	1.272	0.230	0.053	0.820	1.724	5.520	0.000
Doorley et al, 2021	Different chronic diseases	Depressive symptoms	Post-intervention	-0.267	0.446	0.199	-1.142	0.607	-0.600	0.549
Ferguson et al, 2016	Cancer	Depressive symptoms	Post-intervention	-0.532	0.340	0.116	-1.198	0.135	-1.564	0.118
Ferwerda et al, 2017	Arthritis	Depressive symptoms	Post-intervention	0.455	0.198	0.039	0.067	0.843	2.301	0.021
Friesen et al, 2017	Chronic pain	Depressive symptoms	Post-intervention	0.740	0.283	0.080	0.186	1.294	2.616	0.009
Gasslander et al, 2022	Chronic pain	Depressive symptoms	Post-intervention	0.516	0.148	0.022	0.225	0.806	3.482	0.000
Glozier et al, 2013	Cardiovascular disease	Depressive symptoms	Post-intervention	0.282	0.085	0.007	0.116	0.448	3.333	0.001
Ham et al, 2019a	Cancer	Depressive symptoms	Post-intervention	1.022	0.396	0.157	0.246	1.799	2.581	0.010
Ham et al, 2019b	Cancer	Depressive symptoms	Post-intervention	0.495	0.368	0.135	-0.226	1.216	1.345	0.179
Hummel et al, 2017	Cancer	Depressive symptoms	Post-intervention	0.060	0.163	0.026	-0.259	0.378	0.366	0.714
Johansson et al, 2019	Cardiovascular disease	Depressive symptoms	Post-intervention	0.685	0.171	0.029	0.351	1.020	4.017	0.000
Lundgren et al, 2016	Arthritis	Depressive symptoms	Post-intervention	0.354	0.281	0.079	-0.196	0.904	1.261	0.207
Migliorini et al, 2016	Different chronic diseases	Depressive symptoms	Post-intervention	0.227	0.285	0.081	-0.332	0.786	0.796	0.426
Mourad et al, 2016	Chronic pain	Depressive symptoms	Post-intervention	0.256	0.489	0.239	-0.703	1.215	0.523	0.601
Murphy et al, 2018	Cancer	Depressive symptoms	Post-intervention	1.355	0.217	0.047	0.930	1.779	6.252	0.000
Newby et al, 2017	Diabetes	Depressive symptoms	Post-intervention	1.395	0.256	0.066	0.893	1.897	5.448	0.000
O'moore et al, 2018	Arthritis	Depressive symptoms	Post-intervention	1.613	0.293	0.086	1.039	2.186	5.509	0.000
Palermo et al, 2009	Chronic pain	Depressive symptoms	Post-intervention	0.066	0.293	0.086	-0.509	0.640	0.224	0.823
Palermo et al, 2016	Chronic pain	Depressive symptoms	Post-intervention	0.248	0.122	0.015	0.009	0.488	2.035	0.042
Peters et al, 2017	Chronic pain	Depressive symptoms	Post-intervention	1.139	0.181	0.033	0.785	1.494	6.305	0.000
Shigaki et al, 2013	Arthritis	Depressive symptoms	Post-intervention	0.215	0.207	0.043	-0.189	0.620	1.043	0.297
Simblett et al, 2017	Cardiovascular disease	Depressive symptoms	Post-intervention	1.026	0.448	0.201	0.148	1.905	2.289	0.022
Taguchi et al, 2021	Chronic pain	Depressive symptoms	Post-intervention	0.431	0.378	0.143	-0.310	1.172	1.140	0.254
Thesen et al, 2022	Chronic pain	Depressive symptoms	Post-intervention	0.456	0.175	0.031	0.114	0.799	2.611	0.009
Trautmann and Kroner-Herwig, 2010a	Chronic pain	Depressive symptoms	Post-intervention	-0.177	0.407	0.166	-0.974	0.621	-0.434	0.664
Trautmann and Kroner-Herwig, 2010b	Chronic pain	Depressive symptoms	Post-intervention	-0.454	0.401	0.161	-1.239	0.331	-1.134	0.257
Trudeau et al, 2015	Arthritis	Depressive symptoms	Post-intervention	0.070	0.132	0.017	-0.189	0.329	0.531	0.596
Westas et al, 2022	Cardiovascular disease	Depressive symptoms	Post-intervention	0.549	0.212	0.045	0.134	0.964	2.594	0.009
Wiklund et al, 2022	Chronic pain	Depressive symptoms	Post-intervention	-0.024	0.290	0.084	-0.593	0.544	-0.084	0.933
Williams et al, 2010	Chronic pain	Depressive symptoms	Post-intervention	-0.102	0.183	0.034	-0.460	0.257	-0.555	0.579
Wilson et al, 2018	Different chronic diseases	Depressive symptoms	Post-intervention	0.723	0.297	0.088	0.141	1.305	2.434	0.015
				0.448	0.071	0.005	0.309	0.587	6.308	0.000

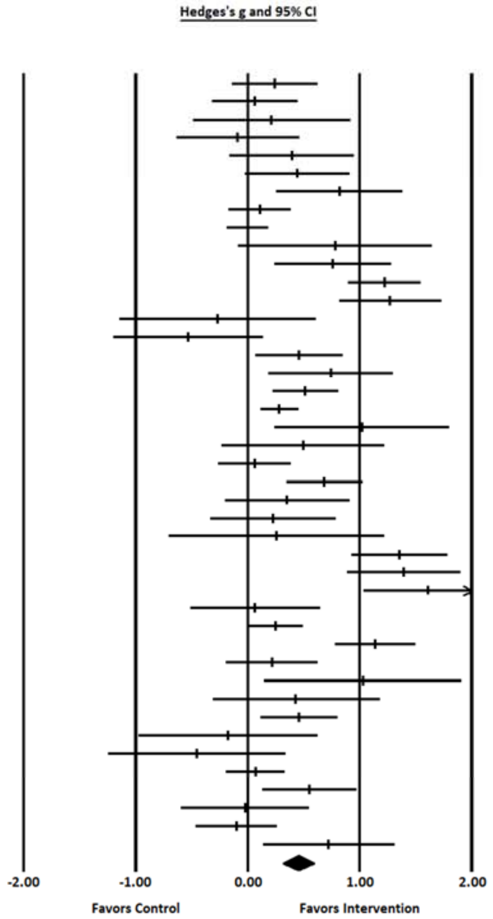

Anxiety symptoms (Post-intervention)

Study name	Condition	Outcome	Time point	Hedges's g	Standard error	Variance	Lower limit	Upper limit	Z-Value	p-Value
Atema et al, 2019a	Cancer	Anxiety symptoms	Post-intervention	0.222	0.193	0.037	-0.155	0.600	1.155	0.248
Atema et al, 2019b	Cancer	Anxiety symptoms	Post-intervention	0.120	0.193	0.037	-0.259	0.499	0.618	0.536
Barroso et al, 2020	HIV	Anxiety symptoms	Post-intervention	-0.612	0.364	0.132	-1.326	0.101	-1.682	0.093
Buhrman et al, 2004	Chronic pain	Anxiety symptoms	Post-intervention	-0.271	0.280	0.078	-0.819	0.277	-0.968	0.333
Buhrman et al, 2011	Chronic pain	Anxiety symptoms	Post-intervention	0.316	0.281	0.079	-0.235	0.867	1.125	0.261
Buhrman et al, 2013	Chronic pain	Anxiety symptoms	Post-intervention	0.491	0.237	0.056	0.027	0.955	2.073	0.038
Buhrman et al, 2015	Chronic pain	Anxiety symptoms	Post-intervention	0.789	0.285	0.081	0.231	1.347	2.772	0.006
Chiauzzi et al, 2010	Chronic pain	Anxiety symptoms	Post-intervention	0.250	0.142	0.020	-0.028	0.528	1.762	0.078
Clarke et al, 2019	Diabetes	Anxiety symptoms	Post-intervention	-0.055	0.092	0.008	-0.235	0.125	-0.600	0.549
Dear et al, 2013	Chronic pain	Anxiety symptoms	Post-intervention	0.820	0.266	0.071	0.299	1.340	3.086	0.002
Dear et al, 2015a	Chronic pain	Anxiety symptoms	Post-intervention	0.662	0.157	0.025	0.355	0.970	4.219	0.000
Dear et al, 2015b	Chronic pain	Anxiety symptoms	Post-intervention	0.786	0.223	0.050	0.349	1.223	3.524	0.000
Doorley et al, 2021	Different chronic diseases	Anxiety symptoms	Post-intervention	0.462	0.450	0.203	-0.420	1.345	1.027	0.304
Ferguson et al, 2016	Cancer	Anxiety symptoms	Post-intervention	-0.717	0.345	0.119	-1.393	-0.041	-2.079	0.038
Ferwerda et al, 2017	Arthritis	Anxiety symptoms	Post-intervention	0.513	0.198	0.039	0.124	0.902	2.585	0.010
Friesen et al, 2017	Chronic pain	Anxiety symptoms	Post-intervention	0.780	0.284	0.081	0.223	1.336	2.746	0.006
Gasslander et al, 2022	Chronic pain	Anxiety symptoms	Post-intervention	0.397	0.173	0.030	0.058	0.736	2.295	0.022
Glozier et al, 2013	Cardiovascular disease	Anxiety symptoms	Post-intervention	0.297	0.085	0.007	0.131	0.463	3.502	0.000
Ham et al, 2019a	Cancer	Anxiety symptoms	Post-intervention	0.975	0.394	0.155	0.202	1.747	2.473	0.013
Ham et al, 2019b	Cancer	Anxiety symptoms	Post-intervention	0.410	0.366	0.134	-0.308	1.128	1.119	0.263
Hummel et al, 2017	Cancer	Anxiety symptoms	Post-intervention	-0.010	0.163	0.026	-0.329	0.309	-0.062	0.951
Lundgren et al, 2016	Arthritis	Anxiety symptoms	Post-intervention	0.197	0.279	0.078	-0.350	0.744	0.707	0.479
Migliorini et al, 2016	Different chronic diseases	Anxiety symptoms	Post-intervention	0.666	0.292	0.085	0.093	1.239	2.279	0.023
Mourad et al, 2016	Chronic pain	Anxiety symptoms	Post-intervention	0.167	0.488	0.238	-0.789	1.124	0.342	0.732
Murphy et al, 2018	Cancer	Anxiety symptoms	Post-intervention	1.508	0.221	0.049	1.074	1.942	6.807	0.000
Newby et al, 2017	Diabetes	Anxiety symptoms	Post-intervention	0.051	0.230	0.053	-0.400	0.502	0.222	0.824
Palermo et al, 2016	Chronic pain	Anxiety symptoms	Post-intervention	0.311	0.122	0.015	0.071	0.551	2.543	0.011
Peters et al, 2017	Chronic pain	Anxiety symptoms	Post-intervention	0.811	0.175	0.031	0.468	1.154	4.630	0.000
Simblett et al, 2017	Cardiovascular disease	Anxiety symptoms	Post-intervention	0.112	0.422	0.178	-0.716	0.940	0.265	0.791
Taguchi et al, 2021	Chronic pain	Anxiety symptoms	Post-intervention	0.023	0.374	0.140	-0.709	0.756	0.063	0.950
Thesen et al, 2022	Chronic pain	Anxiety symptoms	Post-intervention	0.092	0.173	0.030	-0.246	0.430	0.533	0.594
Trudeau et al, 2015	Arthritis	Anxiety symptoms	Post-intervention	0.072	0.132	0.017	-0.187	0.331	0.546	0.585
Wiklund et al, 2022	Chronic pain	Anxiety symptoms	Post-intervention	-0.028	0.290	0.084	-0.597	0.541	-0.097	0.923
Williams et al, 2010	Chronic pain	Anxiety symptoms	Post-intervention	0.100	0.183	0.034	-0.259	0.459	0.547	0.584
				0.322	0.066	0.004	0.193	0.451	4.901	0.000

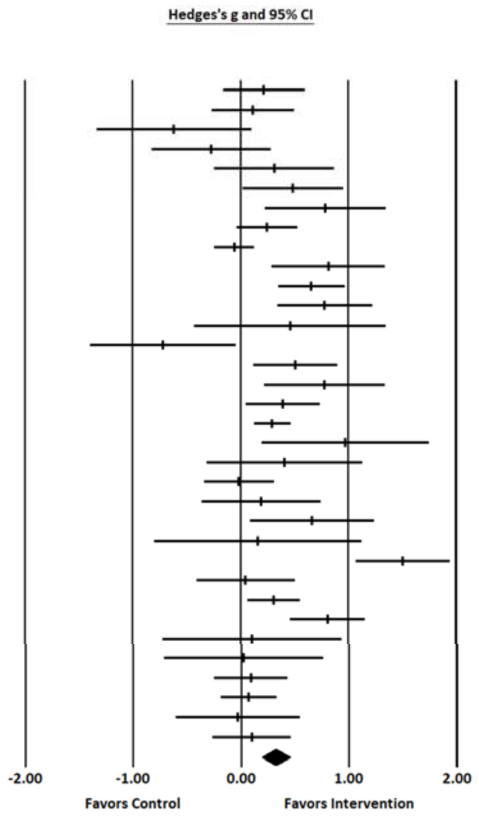

Depressive and anxiety symptoms (Post-intervention)

Study name	Condition	Outcome	Time point	Hedges's g	Standard error	Variance	Lower limit	Upper limit	Z-Value	p-Value
Atema et al, 2019a	Cancer	Depressive & Anxiety symptoms	Post-intervention	0.255	0.193	0.037	-0.123	0.633	1.322	0.186
Atema et al, 2019b	Cancer	Depressive & Anxiety symptoms	Post-intervention	0.106	0.193	0.037	-0.273	0.485	0.548	0.583
Dowd et al, 2015	Chronic pain	Depressive & Anxiety symptoms	Post-intervention	0.045	0.279	0.078	-0.503	0.592	0.159	0.873
Gasslander et al, 2022	Chronic pain	Depressive & Anxiety symptoms	Post-intervention	0.386	0.147	0.022	0.097	0.674	2.622	0.009
Geirhos et al, 2022	Different chronic diseases	Depressive & Anxiety symptoms	Post-intervention	0.273	0.398	0.158	-0.506	1.052	0.687	0.492
Hummel et al, 2017	Cancer	Depressive & Anxiety symptoms	Post-intervention	0.024	0.163	0.026	-0.295	0.342	0.145	0.885
Murphy et al, 2018	Cancer	Depressive & Anxiety symptoms	Post-intervention	2.057	0.242	0.058	1.583	2.531	8.506	0.000
				0.447	0.243	0.059	-0.029	0.922	1.839	0.066

Hedges's g and 95% CI

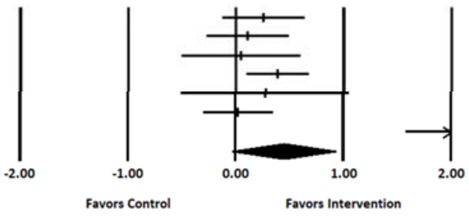

Posttraumatic stress disorder symptoms (Post-intervention)

Study name	Condition	Outcome	Time point	Hedges's g	Standard error	Variance	Lower limit	Upper limit	Z-Value	p-Value
Beatty et al, 2016	Cancer	PTSD symptoms	Post-intervention	2.313	0.358	0.129	1.610	3.016	6.452	0.000
Gasslander et al, 2022	Chronic pain	PTSD symptoms	Post-intervention	0.127	0.239	0.057	-0.341	0.595	0.532	0.595
Geirhos et al, 2022	Different chronic diseases	PTSD symptoms	Post-intervention	0.852	0.414	0.172	0.040	1.664	2.058	0.040
				1.083	0.688	0.474	-0.266	2.432	1.574	0.116

Hedges's g and 95% CI

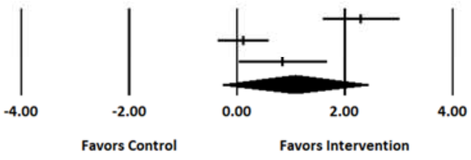

General psychological distress (Post-intervention)

Study name	Condition	Outcome	Time point	Hedges's g	Standard error	Variance	Lower limit	Upper limit	Z-Value	p-Value
Beatty et al, 2016	Cancer	General psychological distress	Post-intervention	0.598	0.282	0.080	0.045	1.151	2.120	0.034
Chambers et al, 2018	Cancer	General psychological distress	Post-intervention	0.263	0.157	0.025	-0.044	0.570	1.679	0.093
Chiauzzi et al, 2010	Chronic pain	General psychological distress	Post-intervention	0.350	0.142	0.020	0.071	0.629	2.455	0.014
Clarke et al, 2019	Diabetes	General psychological distress	Post-intervention	-0.080	0.092	0.008	-0.260	0.100	-0.872	0.383
Migliorini et al, 2016	Different chronic diseases	General psychological distress	Post-intervention	0.606	0.291	0.085	0.036	1.177	2.085	0.037
Murphy et al, 2018	Cancer	General psychological distress	Post-intervention	2.064	0.242	0.059	1.589	2.538	8.525	0.000
Newby et al, 2017	Diabetes	General psychological distress	Post-intervention	1.611	0.264	0.070	1.093	2.129	6.099	0.000
O'moore et al, 2018	Arthritis	General psychological distress	Post-intervention	0.935	0.269	0.072	0.407	1.462	3.473	0.001
Stinson et al, 2010	Arthritis	General psychological distress	Post-intervention	0.126	0.290	0.084	-0.443	0.696	0.435	0.663
Trudeau et al, 2015	Arthritis	General psychological distress	Post-intervention	-0.011	0.132	0.017	-0.270	0.248	-0.081	0.935
				0.623	0.201	0.040	0.229	1.016	3.103	0.002

Statistics for each study

Hedges's g and 95% CI

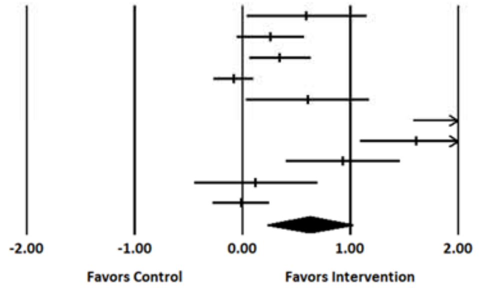

Depressive symptoms (First follow-up)

Study name	Condition	Outcome	Time point	Statistics for each study						
				Hedges's g	Standard error	Variance	Lower limit	Upper limit	Z-Value	p-Value
Atema et al, 2019a	Cancer	Depressive symptoms	First follow-up	0.179	0.193	0.037	-0.199	0.558	0.928	0.354
Atema et al, 2019b	Cancer	Depressive symptoms	First follow-up	0.180	0.196	0.038	-0.204	0.564	0.917	0.359
Buhrman et al, 2004	Chronic pain	Depressive symptoms	First follow-up	-0.062	0.289	0.083	-0.627	0.504	-0.214	0.831
Chiauzzi et al, 2010	Chronic pain	Depressive symptoms	First follow-up	0.265	0.142	0.020	-0.013	0.543	1.866	0.062
Cooper et al, 2011	Multiple sclerosis	Depressive symptoms	First follow-up	0.504	0.459	0.211	-0.397	1.404	1.096	0.273
Ferguson et al, 2016	Cancer	Depressive symptoms	First follow-up	-0.213	0.343	0.117	-0.885	0.458	-0.623	0.534
Ferwerda et al, 2017	Arthritis	Depressive symptoms	First follow-up	0.409	0.233	0.054	-0.047	0.865	1.758	0.079
Gasslander et al, 2022	Chronic pain	Depressive symptoms	First follow-up	0.424	0.269	0.072	-0.102	0.950	1.579	0.114
O'moore et al, 2018	Arthritis	Depressive symptoms	First follow-up	1.475	0.284	0.080	0.920	2.031	5.202	0.000
Palermo et al, 2016	Chronic pain	Depressive symptoms	First follow-up	0.327	0.122	0.015	0.087	0.567	2.670	0.008
Shigaki et al, 2013	Arthritis	Depressive symptoms	First follow-up	0.242	0.212	0.045	-0.174	0.657	1.138	0.255
Simblett et al, 2017	Cardiovascular disease	Depressive symptoms	First follow-up	1.901	0.525	0.276	0.871	2.930	3.618	0.000
Thesen et al, 2022	Chronic pain	Depressive symptoms	First follow-up	0.418	0.179	0.032	0.066	0.770	2.330	0.020
Trautmann and Kroner-Herwig, 2010a	Chronic pain	Depressive symptoms	First follow-up	0.181	0.493	0.243	-0.784	1.147	0.368	0.713
Trautmann and Kroner-Herwig, 2010b	Chronic pain	Depressive symptoms	First follow-up	-0.022	0.522	0.273	-1.046	1.001	-0.043	0.966
Trudeau et al, 2015	Arthritis	Depressive symptoms	First follow-up	0.160	0.132	0.017	-0.099	0.419	1.208	0.227
Westas et al, 2022	Cardiovascular disease	Depressive symptoms	First follow-up	0.623	0.220	0.048	0.192	1.054	2.832	0.005
Wiklund et al, 2022	Chronic pain	Depressive symptoms	First follow-up	-0.712	0.335	0.112	-1.369	-0.055	-2.125	0.034
				0.319	0.091	0.008	0.142	0.497	3.521	0.000

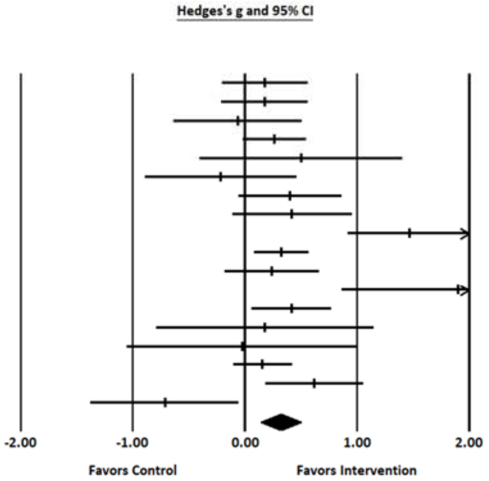

Anxiety symptoms (First follow-up)

Study name	Condition	Outcome	Time point	Hedges's g	Standard error	Variance	Lower limit	Upper limit	Z-Value	p-Value
Atema et al, 2019a	Cancer	Anxiety symptoms	First follow-up	0.068	0.194	0.038	-0.313	0.449	0.349	0.727
Atema et al, 2019b	Cancer	Anxiety symptoms	First follow-up	0.130	0.194	0.038	-0.251	0.510	0.667	0.505
Buhrman et al, 2004	Chronic pain	Anxiety symptoms	First follow-up	-0.276	0.290	0.084	-0.844	0.292	-0.953	0.341
Chiauzzi et al, 2010	Chronic pain	Anxiety symptoms	First follow-up	0.259	0.142	0.020	-0.019	0.537	1.823	0.068
Ferguson et al, 2016	Cancer	Anxiety symptoms	First follow-up	0.349	0.344	0.119	-0.326	1.024	1.014	0.310
Ferwerda et al, 2017	Arthritis	Anxiety symptoms	First follow-up	0.658	0.236	0.056	0.195	1.121	2.784	0.005
Gasslander et al, 2022	Chronic pain	Anxiety symptoms	First follow-up	-0.016	0.464	0.215	-0.925	0.893	-0.034	0.973
Palermo et al, 2016	Chronic pain	Anxiety symptoms	First follow-up	0.227	0.122	0.015	-0.012	0.466	1.859	0.063
Simblett et al, 2017	Cardiovascular disease	Anxiety symptoms	First follow-up	0.040	0.437	0.191	-0.816	0.896	0.091	0.928
Thesen et al, 2022	Chronic pain	Anxiety symptoms	First follow-up	0.445	0.180	0.032	0.093	0.797	2.478	0.013
Trudeau et al, 2015	Arthritis	Anxiety symptoms	First follow-up	0.110	0.132	0.017	-0.148	0.369	0.836	0.403
Wiklund et al, 2022	Chronic pain	Anxiety symptoms	First follow-up	-0.730	0.342	0.117	-1.401	-0.059	-2.133	0.033
				0.171	0.077	0.006	0.020	0.322	2.215	0.027

Hedges's g and 95% CI

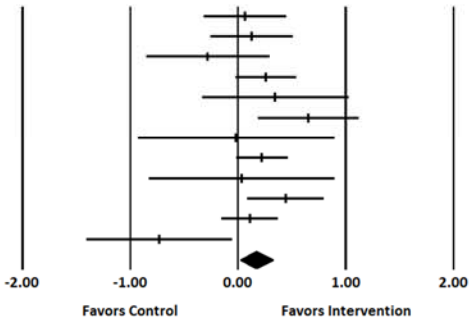

Depressive and anxiety symptoms (First follow-up)

Study name	Condition	Outcome	Time point	Hedges's g	Standard error	Variance	Lower limit	Upper limit	Z-Value	p-Value
Atema et al, 2019a	Cancer	Depressive & Anxiety symptoms	First follow-up	0.132	0.195	0.038	-0.249	0.514	0.681	0.496
Atema et al, 2019b	Cancer	Depressive & Anxiety symptoms	First follow-up	0.171	0.194	0.038	-0.210	0.552	0.879	0.379
Dowd et al, 2015	Chronic pain	Depressive & Anxiety symptoms	First follow-up	0.063	0.279	0.078	-0.485	0.610	0.224	0.822
Gasslander et al, 2022	Chronic pain	Depressive & Anxiety symptoms	First follow-up	0.438	0.269	0.072	-0.089	0.964	1.629	0.103
Geirhos et al, 2022	Different chronic diseases	Depressive & Anxiety symptoms	First follow-up	0.960	0.426	0.182	0.124	1.795	2.250	0.024
				0.241	0.112	0.013	0.020	0.461	2.142	0.032

Hedges's g and 95% CI

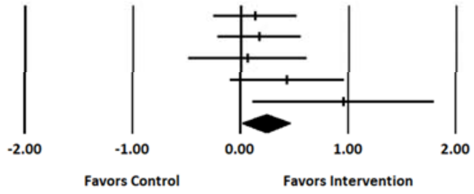

Posttraumatic stress disorder symptoms (First follow-up)

Study name	Condition	Outcome	Time point	Statistics for each study						
				Hedges's g	Standard error	Variance	Lower limit	Upper limit	Z-Value	p-Value
Beatty et al, 2016	Cancer	PTSD symptoms	First follow-up	0.793	0.289	0.084	0.226	1.361	2.741	0.006
Gasslander et al, 2022	Chronic pain	PTSD symptoms	First follow-up	0.781	0.442	0.196	-0.086	1.648	1.767	0.077
Geirhos et al, 2022	Different chronic diseases	PTSD symptoms	First follow-up	1.118	0.435	0.189	0.266	1.970	2.571	0.010
				0.867	0.212	0.045	0.453	1.282	4.100	0.000

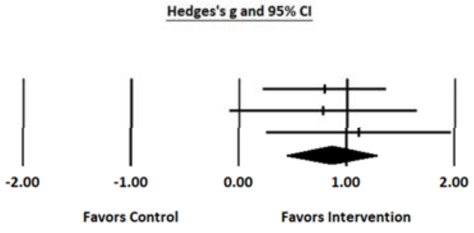

General psychological distress (First follow-up)

Study name	Condition	Outcome	Time point	Statistics for each study						
				Hedges's g	Standard error	Variance	Lower limit	Upper limit	Z-Value	p-Value
Beatty et al, 2016	Cancer	General psychological distress	First follow-up	0.684	0.287	0.082	0.122	1.245	2.384	0.017
Chiauzzi et al, 2010	Chronic pain	General psychological distress	First follow-up	0.516	0.144	0.021	0.235	0.798	3.593	0.000
O'moore et al, 2018	Arthritis	General psychological distress	First follow-up	1.175	0.273	0.074	0.640	1.710	4.308	0.000
Trudeau et al, 2015	Arthritis	General psychological distress	First follow-up	0.163	0.132	0.017	-0.096	0.422	1.233	0.217
				0.581	0.197	0.039	0.195	0.968	2.947	0.003

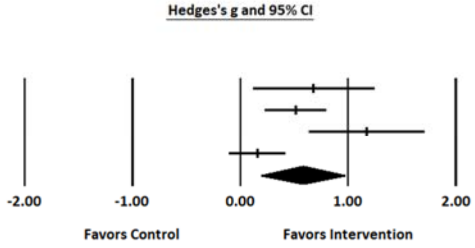

Depressive symptoms (Last follow-up)

Study name	Condition	Outcome	Time point	Hedges's g	Standard error	Variance	Lower limit	Upper limit	Z-Value	p-Value
				Statistics for each study						
Chiauzzi et al, 2010	Chronic pain	Depressive symptoms	Last follow-up	0.303	0.142	0.020	0.024	0.582	2.131	0.033
Ferwerda et al, 2017	Arthritis	Depressive symptoms	Last follow-up	0.439	0.249	0.062	-0.049	0.927	1.762	0.078
Thesen et al, 2022	Chronic pain	Depressive symptoms	Last follow-up	0.136	0.184	0.034	-0.225	0.497	0.739	0.460
Trudeau et al, 2015	Arthritis	Depressive symptoms	Last follow-up	0.393	0.133	0.018	0.131	0.654	2.946	0.003
Westas et al, 2022	Cardiovascular disease	Depressive symptoms	Last follow-up	0.670	0.231	0.053	0.217	1.123	2.900	0.004
				0.357	0.077	0.006	0.207	0.507	4.656	0.000

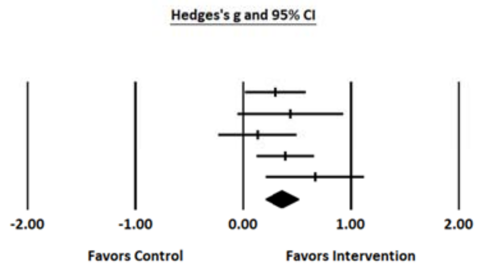

Anxiety symptoms (Last follow-up)

Study name	Condition	Outcome	Time point	Hedges's g	Standard error	Variance	Lower limit	Upper limit	Z-Value	p-Value
				Statistics for each study						
Chiauzzi et al, 2010	Chronic pain	Anxiety symptoms	Last follow-up	0.320	0.142	0.020	0.041	0.599	2.248	0.025
Ferwerda et al, 2017	Arthritis	Anxiety symptoms	Last follow-up	0.280	0.245	0.060	-0.200	0.760	1.144	0.253
Thesen et al, 2022	Chronic pain	Anxiety symptoms	Last follow-up	0.457	0.186	0.035	0.092	0.822	2.455	0.014
Trudeau et al, 2015	Arthritis	Anxiety symptoms	Last follow-up	0.266	0.133	0.018	0.006	0.526	2.006	0.045
				0.321	0.081	0.007	0.162	0.481	3.960	0.000

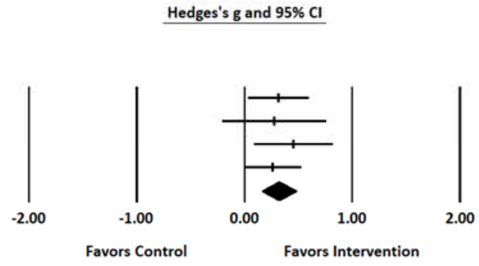

Posttraumatic stress disorder symptoms (Last follow-up)

Study name	Condition	Outcome	Time point	Hedges's g	Standard error	Variance	Lower limit	Upper limit	Z-Value	p-Value
Beatty et al, 2016	Cancer	PTSD symptoms	Last follow-up	0.576	0.282	0.079	0.024	1.128	2.044	0.041
				0.576	0.282	0.079	0.024	1.128	2.044	0.041

Statistics for each study

Hedges's g and 95% CI

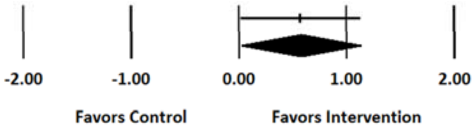

General psychological distress (Last follow-up)

Study name	Condition	Outcome	Time point	Hedges's g	Standard error	Variance	Lower limit	Upper limit	Z-Value	p-Value
Beatty et al, 2016	Cancer	General psychological distress	Last follow-up	1.495	0.313	0.098	0.881	2.108	4.776	0.000
Chiauzzi et al, 2010	Chronic pain	General psychological distress	Last follow-up	0.466	0.143	0.021	0.186	0.747	3.255	0.001
Trudeau et al, 2015	Arthritis	General psychological distress	Last follow-up	0.319	0.133	0.018	0.059	0.580	2.404	0.016
				0.673	0.251	0.063	0.180	1.165	2.676	0.007

Statistics for each study

Hedges's g and 95% CI

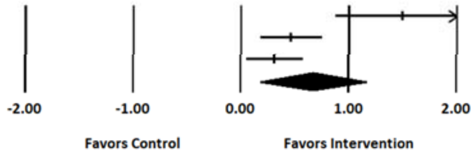

Physical symptoms (Post-intervention)

Study name	Condition	Outcome	Time point	Hedges's g	Standard error	Variance	Lower limit	Upper limit	Z-Value	p-Value
Atema et al, 2019a	Cancer	Combined	Post-intervention	0.233	0.195	0.038	-0.150	0.616	1.193	0.233
Atema et al, 2019b	Cancer	Combined	Post-intervention	0.147	0.194	0.037	-0.233	0.526	0.757	0.449
Barroso et al, 2020	HIV	Combined	Post-intervention	0.006	0.357	0.127	-0.693	0.706	0.018	0.986
Buhrman et al, 2004	Chronic pain	Combined	Post-intervention	-0.215	0.279	0.078	-0.763	0.332	-0.770	0.441
Buhrman et al, 2011	Chronic pain	Pain severity	Post-intervention	0.263	0.281	0.079	-0.287	0.813	0.938	0.348
Buhrman et al, 2013	Chronic pain	Pain severity	Post-intervention	0.254	0.234	0.055	-0.205	0.712	1.083	0.279
Buhrman et al, 2015	Chronic pain	Pain severity	Post-intervention	0.137	0.274	0.075	-0.400	0.675	0.501	0.616
Dear et al, 2013	Chronic pain	Average pain	Post-intervention	0.769	0.264	0.070	0.251	1.287	2.909	0.004
Dear et al, 2015a	Chronic pain	Average pain	Post-intervention	0.383	0.155	0.024	0.079	0.688	2.466	0.014
Dear et al, 2015b	Chronic pain	Average pain	Post-intervention	0.190	0.219	0.048	-0.239	0.618	0.867	0.386
Doorley et al, 2021	Different chronic diseases	Combined	Post-intervention	1.009	0.473	0.224	0.082	1.936	2.132	0.033
Dowd et al, 2015	Chronic pain	Combined	Post-intervention	0.137	0.291	0.085	-0.433	0.707	0.470	0.638
Ferguson et al, 2016	Cancer	Fatigue	Post-intervention	0.059	0.334	0.112	-0.597	0.714	0.176	0.860
Ferwerda et al, 2017	Arthritis	Combined	Post-intervention	0.101	0.197	0.039	-0.284	0.487	0.515	0.606
Friesen et al, 2017	Chronic pain	Combined	Post-intervention	0.550	0.279	0.078	0.003	1.098	1.971	0.049
Gasslander et al, 2022	Chronic pain	Combined	Post-intervention	0.293	0.156	0.024	-0.014	0.599	1.871	0.061
Hummel et al, 2017	Cancer	Combined	Post-intervention	-0.151	0.163	0.027	-0.471	0.168	-0.927	0.354
Mourad et al, 2016	Chronic pain	Body sensations	Post-intervention	-0.291	0.490	0.240	-1.251	0.670	-0.593	0.553
O'moore et al, 2018	Arthritis	Combined	Post-intervention	0.334	0.259	0.067	-0.173	0.841	1.291	0.197
Palermo et al, 2009	Chronic pain	Combined	Post-intervention	0.765	0.306	0.094	0.165	1.366	2.500	0.012
Palermo et al, 2016	Chronic pain	Pain intensity	Post-intervention	0.110	0.122	0.015	-0.129	0.348	0.902	0.367
Peters et al, 2017	Chronic pain	Pain intensity	Post-intervention	0.099	0.169	0.029	-0.233	0.431	0.587	0.557
Shigaki et al, 2013	Arthritis	Combined	Post-intervention	0.163	0.206	0.043	-0.241	0.568	0.792	0.429
Stinson et al, 2010	Arthritis	General symptoms	Post-intervention	-0.165	0.291	0.084	-0.734	0.405	-0.567	0.571
Taguchi et al, 2021	Chronic pain	Combined	Post-intervention	0.284	0.376	0.141	-0.453	1.020	0.755	0.450
Thesen et al, 2022	Chronic pain	Body sensations	Post-intervention	0.249	0.173	0.030	-0.091	0.588	1.436	0.151
Trautmann and Kroner-Herwig, 2010a	Chronic pain	Combined	Post-intervention	0.464	0.435	0.189	-0.389	1.316	1.065	0.287
Trautmann and Kroner-Herwig, 2010b	Chronic pain	Combined	Post-intervention	0.285	0.435	0.189	-0.567	1.137	0.656	0.512
Trudeau et al, 2015	Arthritis	Combined	Post-intervention	0.028	0.132	0.017	-0.230	0.287	0.215	0.830
Wiklund et al, 2022	Chronic pain	Pain intensity	Post-intervention	-0.228	0.291	0.085	-0.799	0.342	-0.784	0.433
Williams et al, 2010	Chronic pain	Combined	Post-intervention	0.383	0.186	0.034	0.019	0.747	2.065	0.039
				0.184	0.040	0.002	0.106	0.263	4.585	0.000

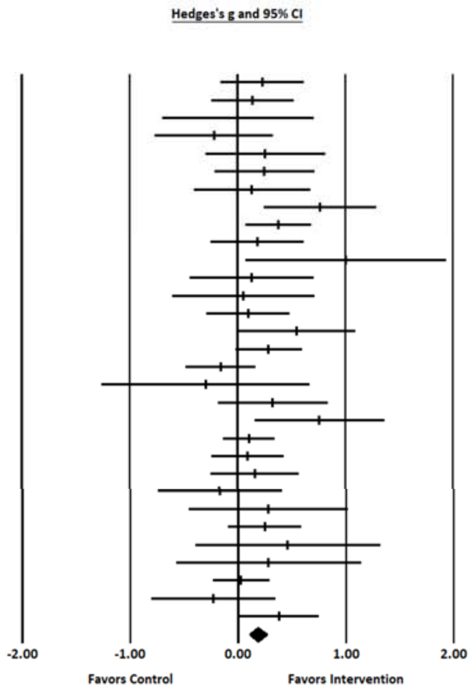

Notes. "Combined" (under the column "Outcome") indicates that multiple outcomes on physical symptoms were retrieved and averaged from the same comparison.

Functional impairment (Post-intervention)

Study name	Condition	Outcome	Time point	Hedges's g	Standard error	Variance	Lower limit	Upper limit	Z-Value	p-Value
Atema et al, 2019a	Cancer	Combined	Post-intervention	0.566	0.196	0.039	0.181	0.951	2.883	0.004
Atema et al, 2019b	Cancer	Combined	Post-intervention	0.433	0.196	0.038	0.050	0.816	2.214	0.027
Barroso et al, 2020	HIV	HRFS overall fatigue-related functioning	Post-intervention	0.084	0.355	0.126	-0.613	0.780	0.236	0.814
Buhrman et al, 2015	Chronic pain	Pain disability	Post-intervention	0.546	0.279	0.078	-0.001	1.094	1.957	0.050
Chiauzzi et al, 2010	Chronic pain	Disability (total score)	Post-intervention	0.051	0.141	0.020	-0.226	0.329	0.364	0.716
Cooper et al, 2011	Multiple sclerosis	Physical impact	Post-intervention	0.104	0.437	0.191	-0.754	0.961	0.237	0.813
Dear et al, 2013	Chronic pain	Disability	Post-intervention	0.764	0.264	0.070	0.246	1.282	2.893	0.004
Dear et al, 2015a	Chronic pain	Disability	Post-intervention	0.560	0.156	0.024	0.253	0.866	3.582	0.000
Dear et al, 2015b	Chronic pain	Disability	Post-intervention	0.648	0.221	0.049	0.214	1.082	2.926	0.003
Doorley et al, 2021	Different chronic diseases	Combined	Post-intervention	0.177	0.446	0.199	-0.698	1.051	0.396	0.692
Gasslander et al, 2022	Chronic pain	Pain disability	Post-intervention	0.188	0.147	0.022	-0.099	0.476	1.282	0.200
Glozier et al, 2013	Cardiovascular disease	Disability (total score)	Post-intervention	0.072	0.084	0.007	-0.093	0.238	0.859	0.391
Hummel et al, 2017	Cancer	Combined	Post-intervention	0.264	0.166	0.028	-0.062	0.590	1.588	0.112
O'moore et al, 2018	Arthritis	OA-specific physical function	Post-intervention	0.426	0.259	0.067	-0.081	0.934	1.647	0.100
Palermo et al, 2016	Chronic pain	Sleep quality	Post-intervention	0.193	0.122	0.015	-0.046	0.432	1.581	0.114
Peters et al, 2017	Chronic pain	Physical impairment	Post-intervention	0.216	0.170	0.029	-0.117	0.549	1.272	0.203
Stinson et al, 2010	Arthritis	Combined	Post-intervention	0.099	0.290	0.084	-0.470	0.668	0.341	0.733
Taguchi et al, 2021	Chronic pain	Pain disability	Post-intervention	1.156	0.405	0.164	0.361	1.950	2.852	0.004
Trudeau et al, 2015	Arthritis	Physical	Post-intervention	-0.008	0.132	0.017	-0.267	0.250	-0.063	0.950
Wiklund et al, 2022	Chronic pain	Pain disability	Post-intervention	0.137	0.290	0.084	-0.432	0.706	0.472	0.637
Williams et al, 2010	Chronic pain	Physical functioning	Post-intervention	0.318	0.184	0.034	-0.043	0.678	1.725	0.084
				0.284	0.054	0.003	0.178	0.390	5.250	0.000

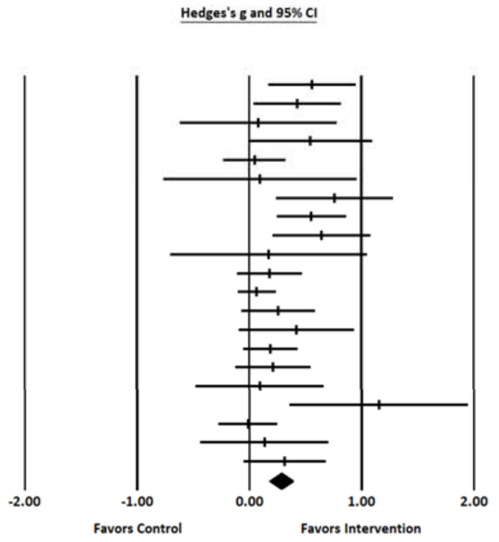

Notes. "Combined" (under the column "Outcome") indicates that multiple outcomes on functional impairment were retrieved and averaged from the same comparison.

Self-rated ill health (Post-intervention)

Study name	Condition	Outcome	Time point	Hedges's g	Standard error	Variance	Lower limit	Upper limit	Z-Value	p-Value
				Statistics for each study						
Friesen et al, 2017	Chronic pain	Physical health	Post-intervention	0.524	0.278	0.077	-0.021	1.069	1.884	0.060
Newby et al, 2017	Diabetes	Physical well-being	Post-intervention	0.120	0.230	0.053	-0.331	0.572	0.523	0.601
Shigaki et al, 2013	Arthritis	Overall health (physical)	Post-intervention	0.120	0.206	0.043	-0.284	0.524	0.582	0.561
Wilson et al, 2018	Different chronic diseases	General health	Post-intervention	-0.496	0.292	0.085	-1.068	0.077	-1.698	0.090
				0.080	0.183	0.033	-0.279	0.438	0.435	0.664

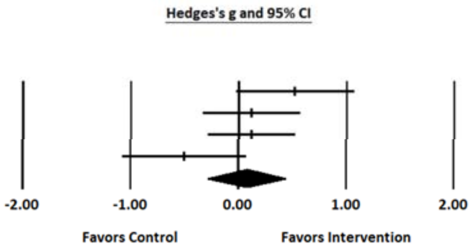

Objective physiological dysfunction (Post-intervention)

Study name	Condition	Outcome	Time point	Hedges's g	Standard error	Variance	Lower limit	Upper limit	Z-Value	p-Value
				Statistics for each study						
Barroso et al, 2020	HIV	Combined	Post-intervention	0.338	0.359	0.129	-0.366	1.042	0.941	0.347
Newby et al, 2017	Diabetes	Haemoglobin A1c	Post-intervention	-0.072	0.230	0.053	-0.523	0.379	-0.312	0.755
				0.047	0.194	0.038	-0.332	0.427	0.245	0.807

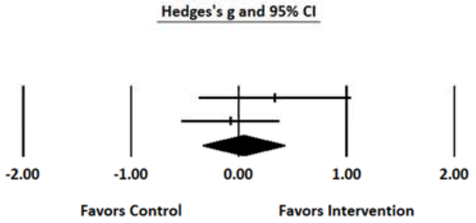

Notes. "Combined" (under the column "Outcome") indicates that multiple outcomes on objective physiological dysfunction were retrieved and averaged from the same comparison.

Physical symptoms (First follow-up)

Study name	Condition	Outcome	Time point	Hedges's g	Standard error	Variance	Lower limit	Upper limit	Z-Value	p-Value
				Statistics for each study						
Atema et al, 2019a	Cancer	Combined	First follow-up	0.234	0.195	0.038	-0.148	0.616	1.199	0.231
Atema et al, 2019b	Cancer	Combined	First follow-up	0.200	0.197	0.039	-0.185	0.586	1.019	0.308
Buhrman et al, 2004	Chronic pain	Combined	First follow-up	-0.763	0.299	0.089	-1.350	-0.177	-2.552	0.011
Dowd et al, 2015	Chronic pain	Combined	First follow-up	-0.198	0.280	0.078	-0.747	0.350	-0.708	0.479
Ferguson et al, 2016	Cancer	Fatigue	First follow-up	-0.603	0.349	0.122	-1.287	0.082	-1.725	0.084
Ferwerda et al, 2017	Arthritis	Combined	First follow-up	0.408	0.234	0.055	-0.050	0.866	1.745	0.081
Gasslander et al, 2022	Chronic pain	Combined	First follow-up	-0.472	0.303	0.092	-1.066	0.121	-1.559	0.119
O'moore et al, 2018	Arthritis	Combined	First follow-up	1.041	0.269	0.072	0.514	1.567	3.875	0.000
Palermo et al, 2016	Chronic pain	Pain intensity	First follow-up	0.100	0.122	0.015	-0.138	0.339	0.824	0.410
Shigaki et al, 2013	Arthritis	Combined	First follow-up	0.003	0.211	0.045	-0.411	0.417	0.014	0.989
Thesen et al, 2022	Chronic pain	Body sensations	First follow-up	0.290	0.178	0.032	-0.059	0.640	1.627	0.104
Trautmann and Kroner-Herwig, 2010a	Chronic pain	Combined	First follow-up	0.216	0.459	0.211	-0.684	1.117	0.471	0.638
Trautmann and Kroner-Herwig, 2010b	Chronic pain	Combined	First follow-up	0.307	0.510	0.260	-0.693	1.307	0.601	0.548
Trudeau et al, 2015	Arthritis	Combined	First follow-up	0.044	0.132	0.017	-0.214	0.303	0.336	0.737
Wiklund et al, 2022	Chronic pain	Pain intensity	First follow-up	-0.558	0.331	0.110	-1.207	0.091	-1.684	0.092
				0.047	0.099	0.010	-0.147	0.241	0.474	0.636

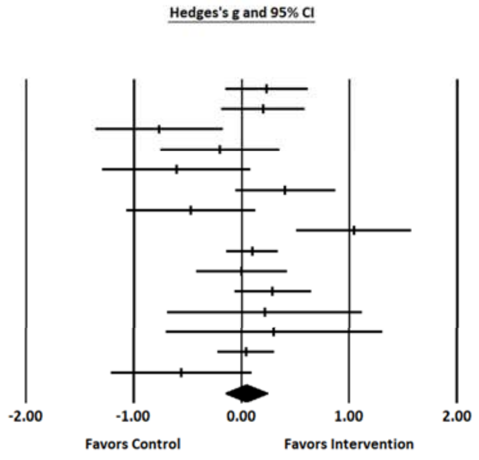

Notes. “Combined” (under the column “Outcome”) indicates that multiple outcomes on physical symptoms were retrieved and averaged from the same comparison.

Functional impairment (First follow-up)

Study name	Condition	Outcome	Time point	Hedges's g	Standard error	Variance	Lower limit	Upper limit	Z-Value	p-Value
				Statistics for each study						
Atema et al, 2019a	Cancer	Combined	First follow-up	0.456	0.197	0.039	0.071	0.842	2.319	0.020
Atema et al, 2019b	Cancer	Combined	First follow-up	0.344	0.196	0.038	-0.040	0.727	1.757	0.079
Chiauzzi et al, 2010	Chronic pain	Disability (total score)	First follow-up	-0.020	0.141	0.020	-0.297	0.257	-0.143	0.887
Cooper et al, 2011	Multiple sclerosis	Physical impact	First follow-up	0.451	0.445	0.198	-0.421	1.324	1.015	0.310
Gasslander et al, 2022	Chronic pain	Pain disability	First follow-up	-0.210	0.266	0.071	-0.732	0.312	-0.789	0.430
O'moore et al, 2018	Arthritis	OA-specific physical function	First follow-up	0.982	0.267	0.071	0.458	1.505	3.677	0.000
Palermo et al, 2016	Chronic pain	Sleep quality	First follow-up	0.227	0.122	0.015	-0.012	0.466	1.863	0.062
Trudeau et al, 2015	Arthritis	Physical	First follow-up	-0.050	0.132	0.017	-0.309	0.209	-0.377	0.706
Wiklund et al, 2022	Chronic pain	Pain disability	First follow-up	-0.461	0.336	0.113	-1.119	0.198	-1.371	0.170
				0.182	0.113	0.013	-0.039	0.403	1.615	0.106

Hedges's g and 95% CI

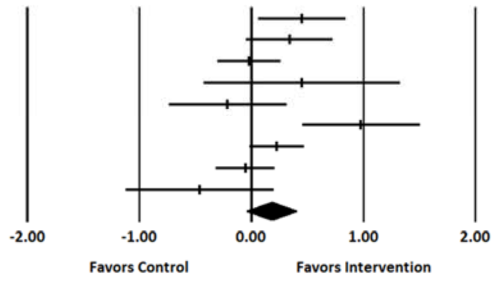

Notes. "Combined" (under the column "Outcome") indicates that multiple outcomes on functional impairment were retrieved and averaged from the same comparison.

Self-rated ill health (First follow-up)

Study name	Condition	Outcome	Time point	Hedges's g	Standard error	Variance	Lower limit	Upper limit	Z-Value	p-Value
				Statistics for each study						
Shigaki et al, 2013	Arthritis	Overall health (physical)	First follow-up	0.000	0.211	0.045	-0.414	0.414	0.000	1.000
				0.000	0.211	0.045	-0.414	0.414	0.000	1.000

Hedges's g and 95% CI

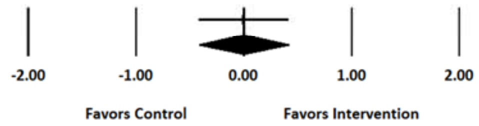

Physical symptoms (Last follow-up)

Study name	Condition	Outcome	Time point	Hedges's g	Standard error	Variance	Lower limit	Upper limit	Z-Value	p-Value
				Statistics for each study						
Ferwerda et al, 2017	Arthritis	Combined	Last follow-up	0.055	0.249	0.062	-0.433	0.543	0.221	0.825
Thesen et al, 2022	Chronic pain	Body sensations	Last follow-up	0.238	0.185	0.034	-0.124	0.599	1.288	0.198
Trudeau et al, 2015	Arthritis	Combined	Last follow-up	0.205	0.132	0.018	-0.054	0.465	1.549	0.121
				0.191	0.099	0.010	-0.003	0.384	1.933	0.053

Hedges's g and 95% CI

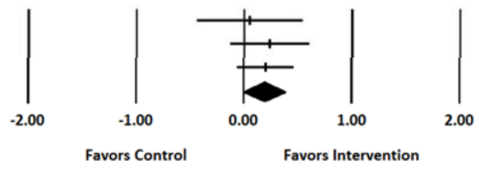

Notes. “Combined” (under the column “Outcome”) indicates that multiple outcomes on physical symptoms were retrieved and averaged from the same comparison.

Functional impairment (Last follow-up)

Study name	Condition	Outcome	Time point	Hedges's g	Standard error	Variance	Lower limit	Upper limit	Z-Value	p-Value
				Statistics for each study						
Chiauzzi et al, 2010	Chronic pain	Disability (total score)	Last follow-up	-0.052	0.141	0.020	-0.329	0.225	-0.367	0.714
Trudeau et al, 2015	Arthritis	Physical	Last follow-up	0.016	0.132	0.017	-0.243	0.275	0.123	0.902
				-0.016	0.096	0.009	-0.205	0.174	-0.161	0.872

Hedges's g and 95% CI

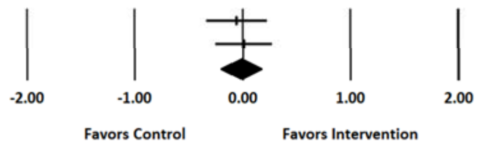

SUPPLEMENTARY FIGURE 2 Funnel plots (*n*=44 studies).

Depressive symptoms (Post-intervention)

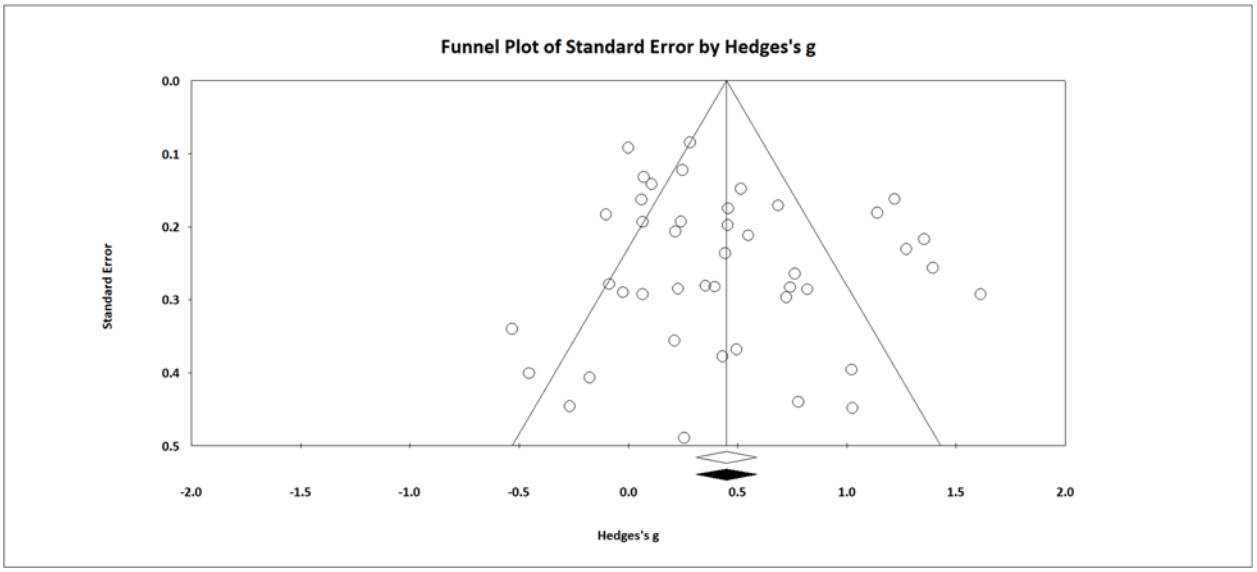

Anxiety symptoms (Post-intervention)

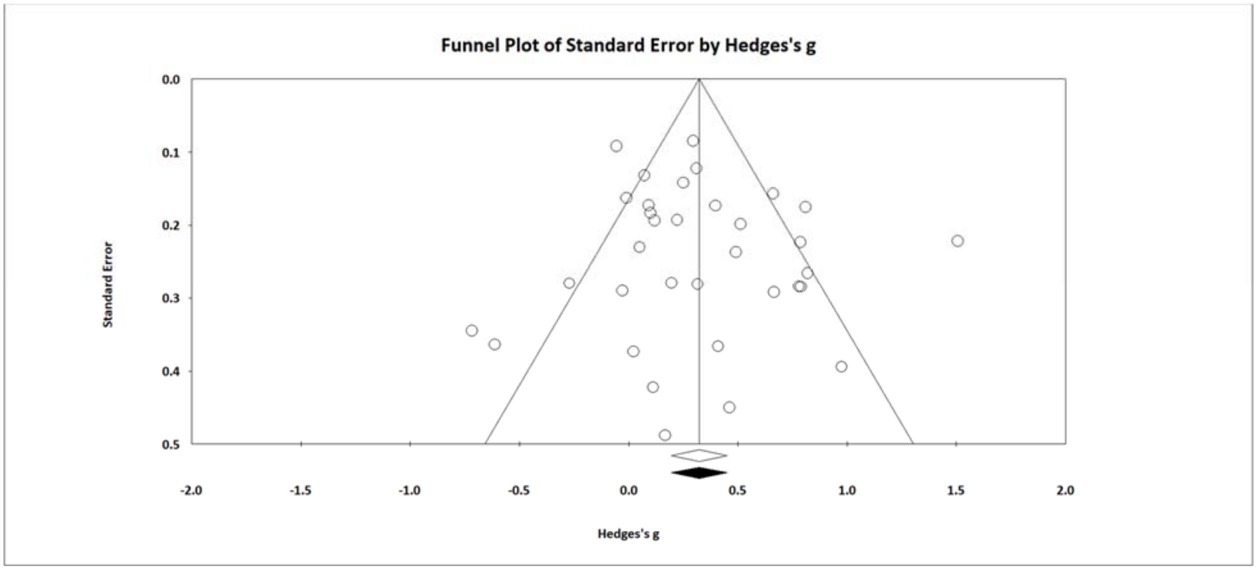

Depressive and anxiety symptoms (Post-intervention)

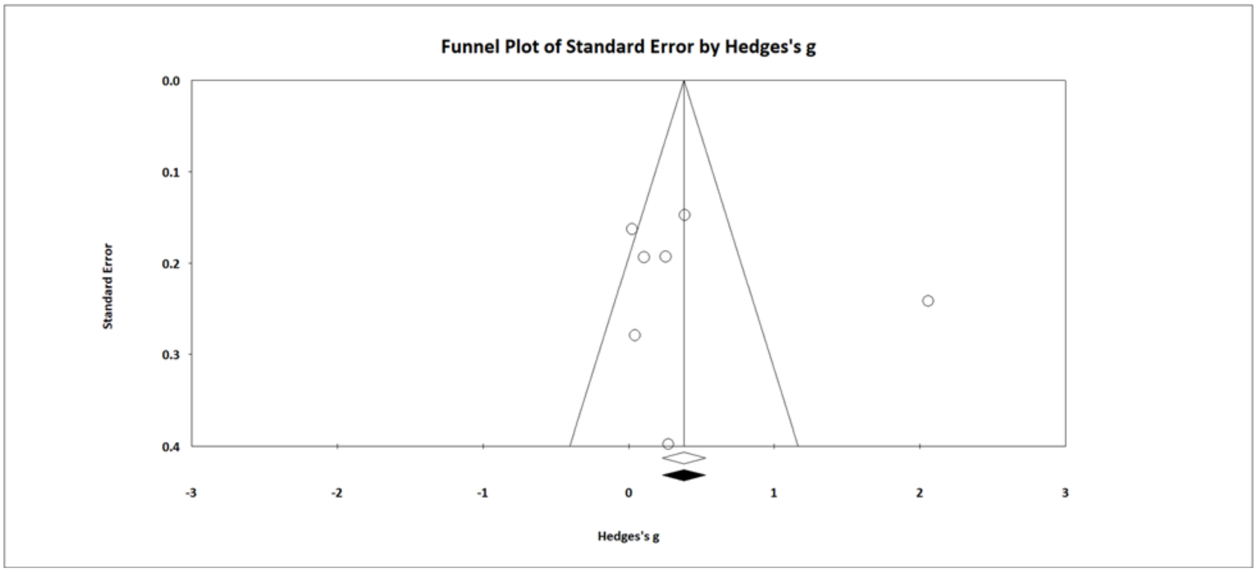

Posttraumatic stress disorder symptoms (Post-intervention)

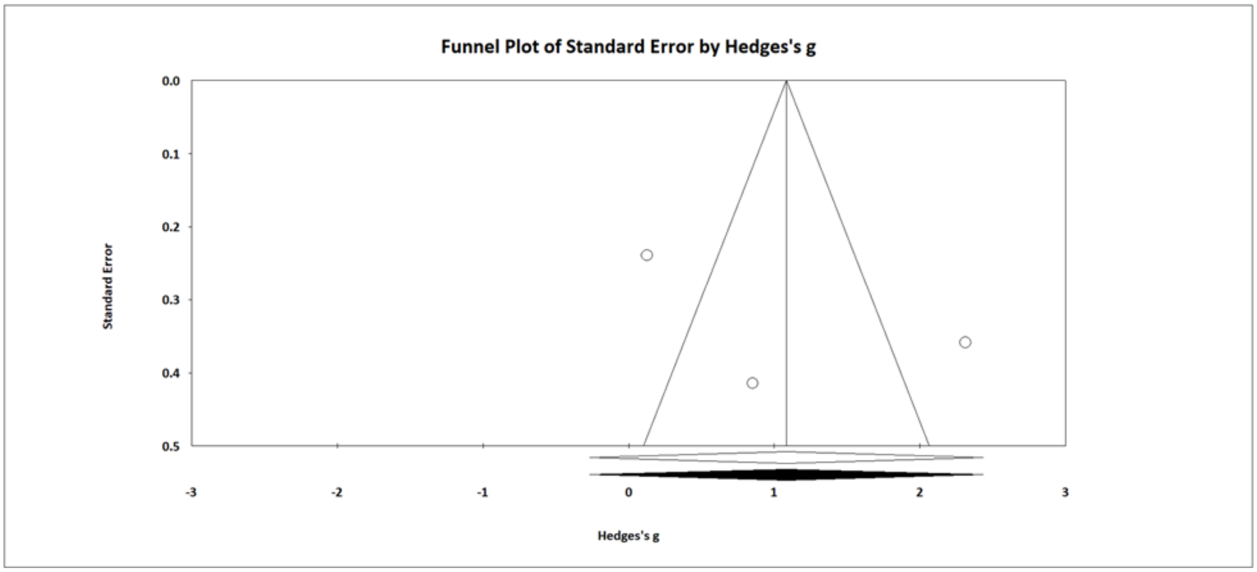

General psychological distress (Post-intervention)

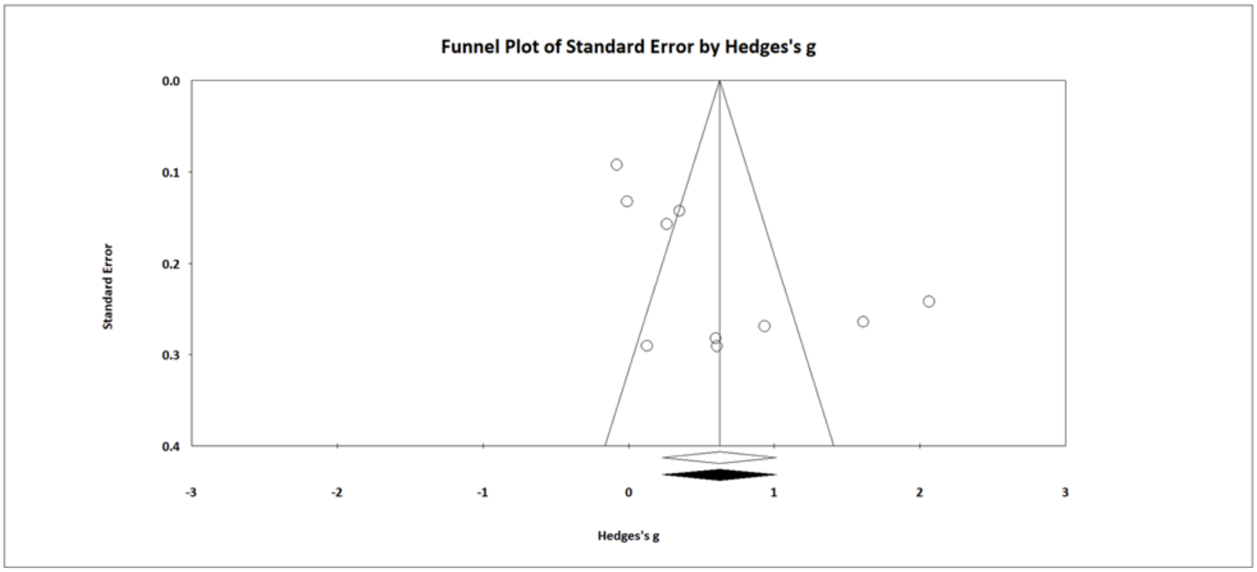

Depressive symptoms (First follow-up)

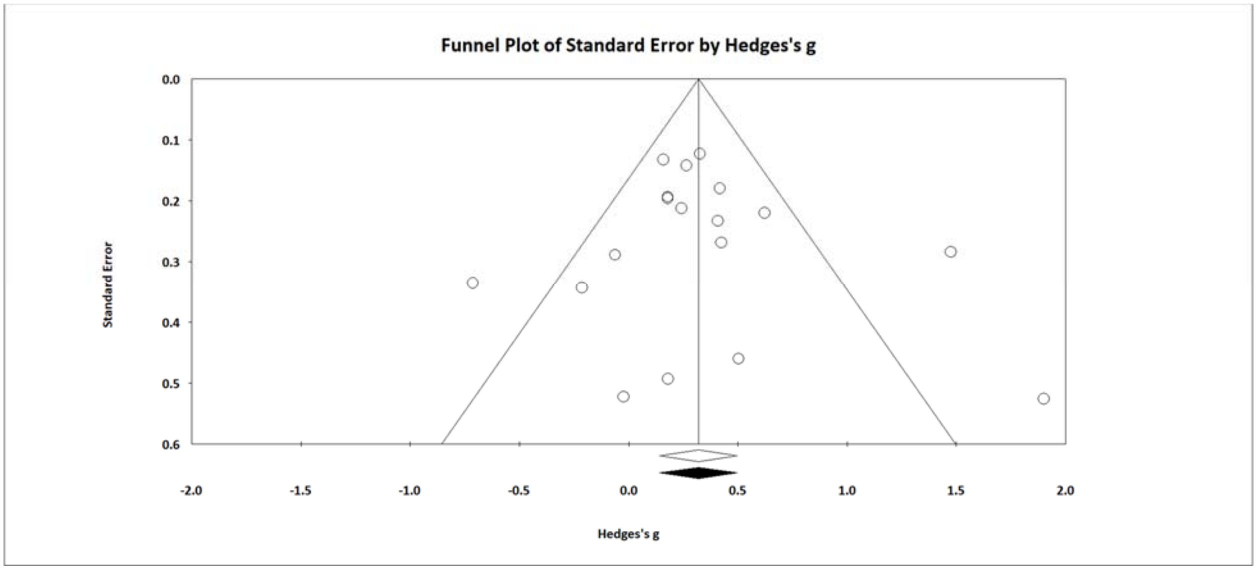

Anxiety symptoms (First follow-up)

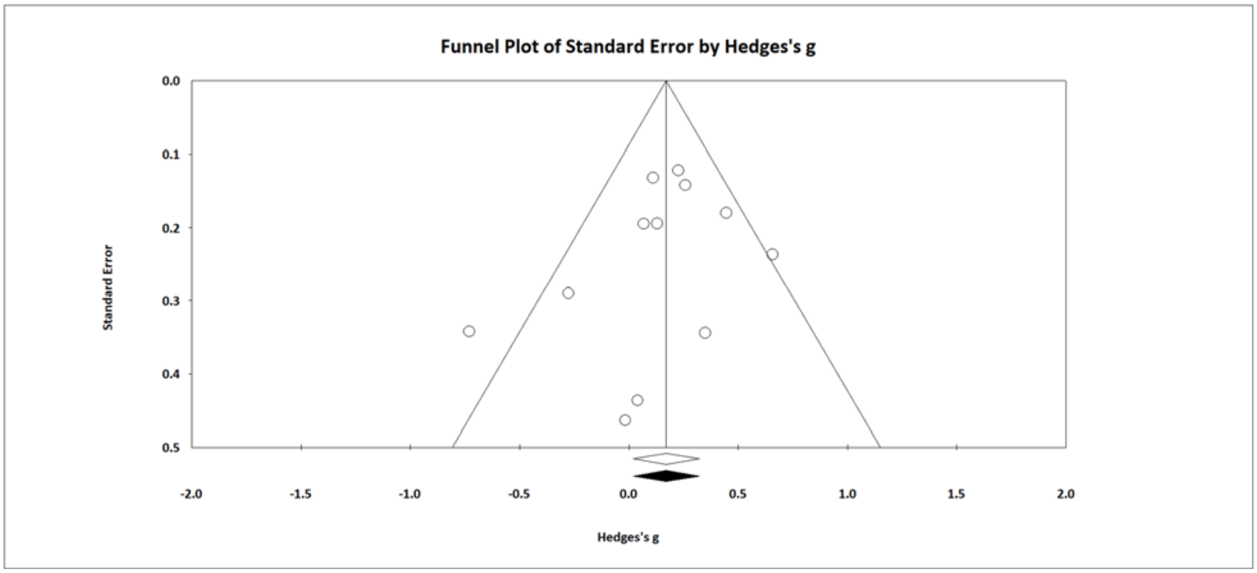

Depressive and anxiety symptoms (First follow-up)

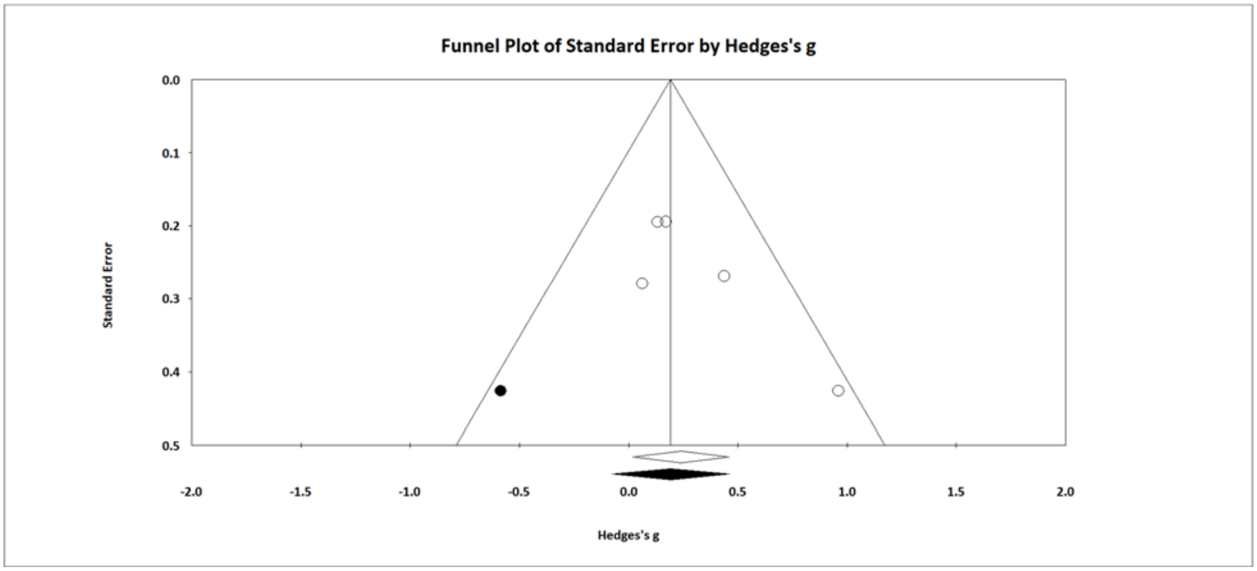

Posttraumatic stress disorder symptoms (First follow-up)

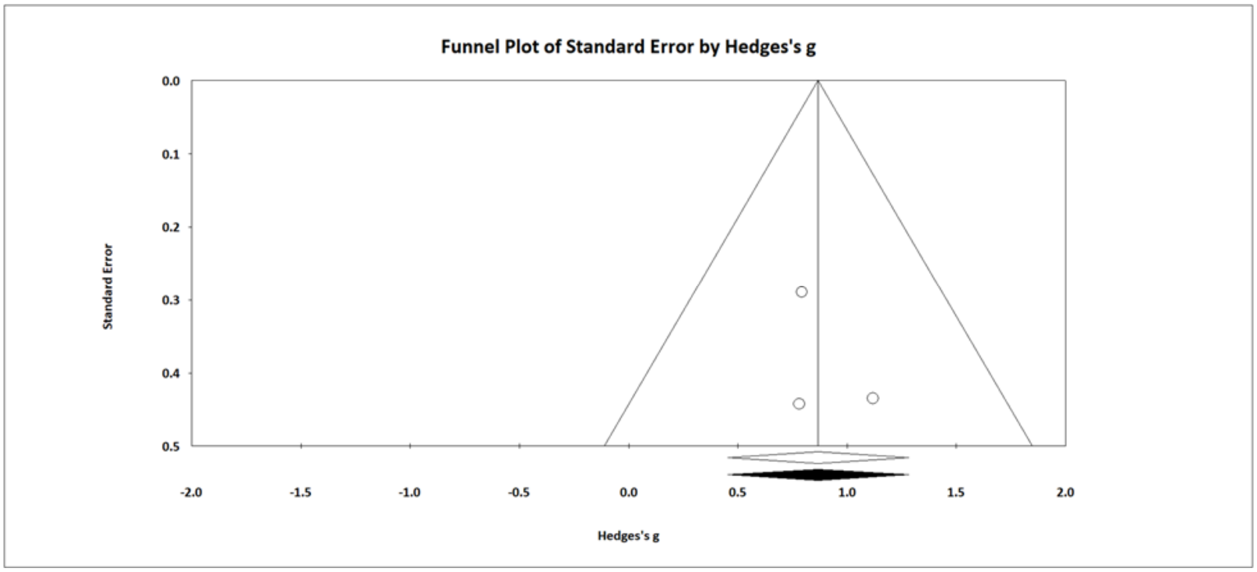

General psychological distress (First follow-up)

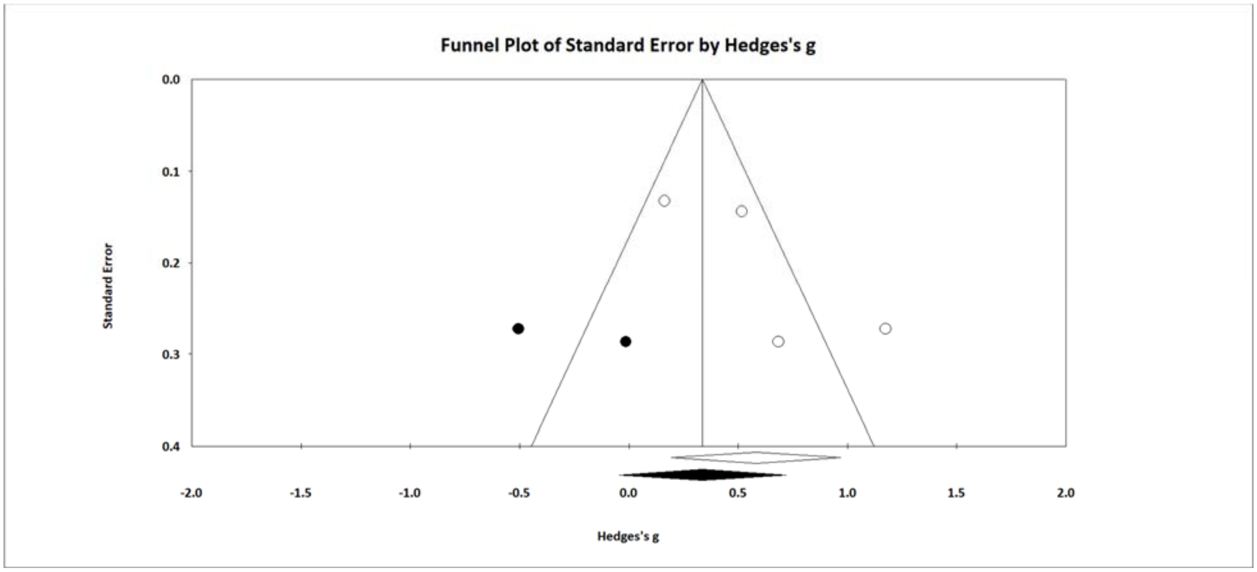

Depressive symptoms (Last follow-up)

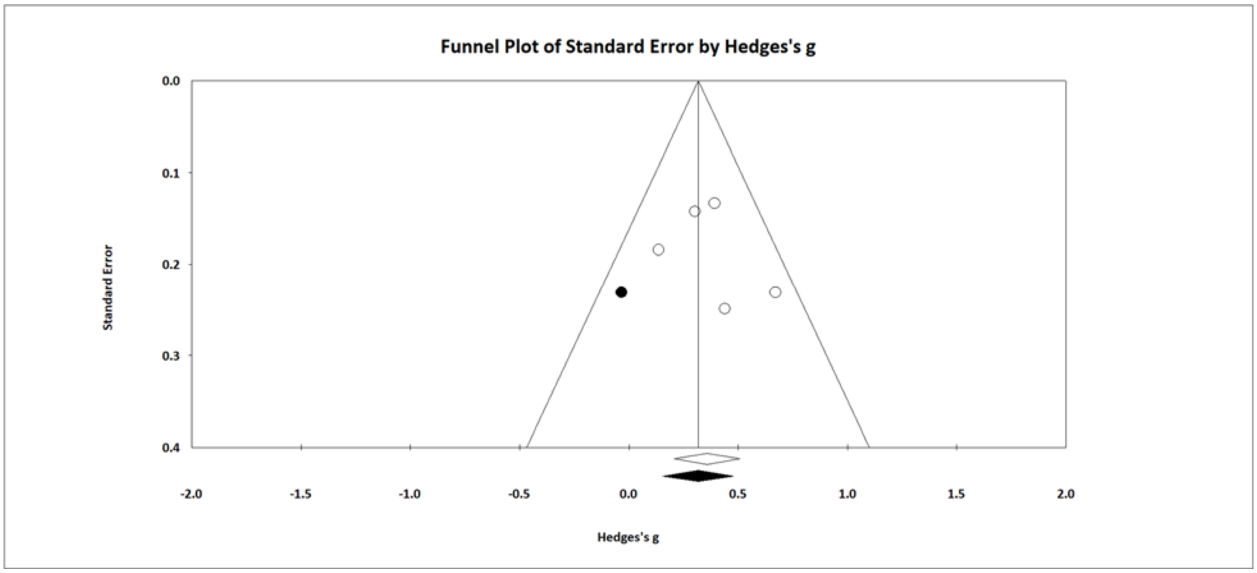

Anxiety symptoms (Last follow-up)

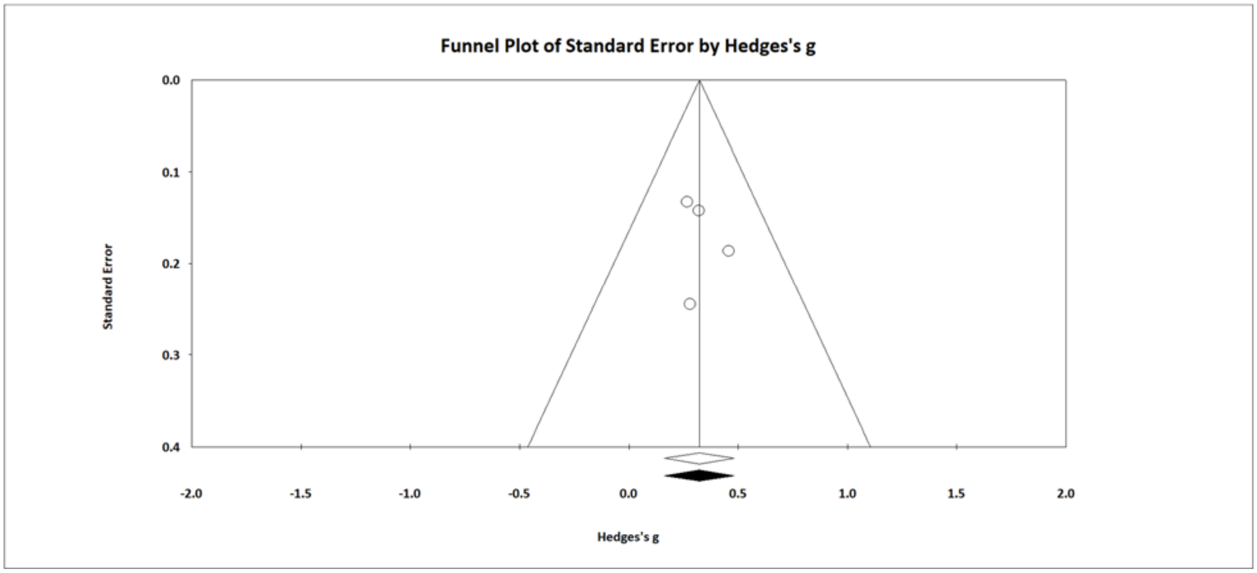

General psychological distress (Last follow-up)

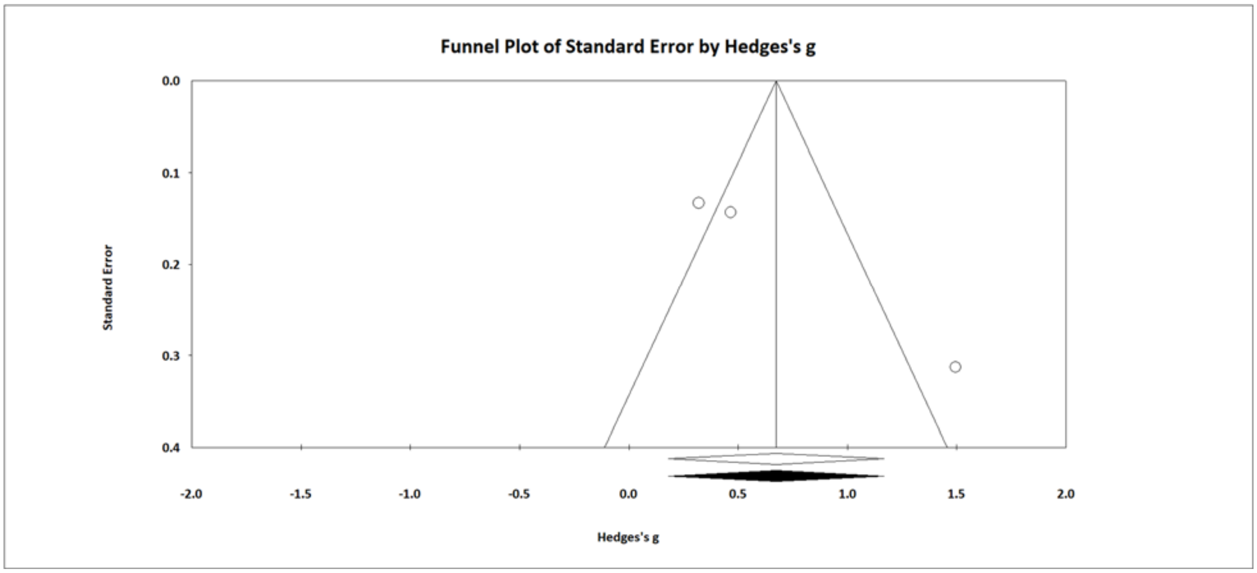

Physical symptoms (Post-intervention)

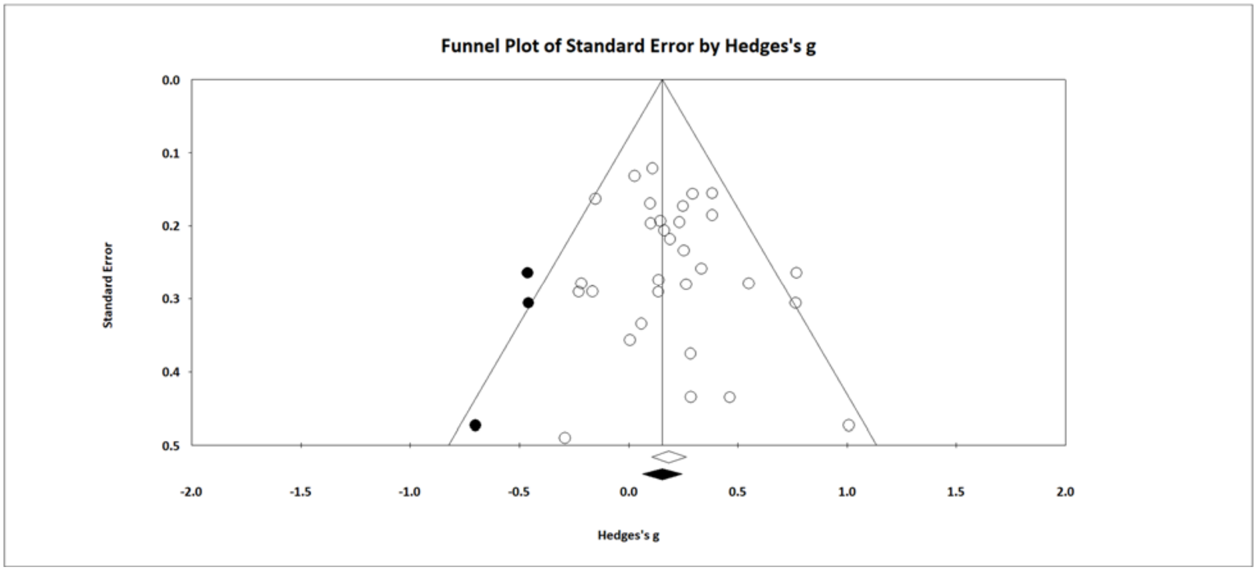

Functional impairment (Post-intervention)

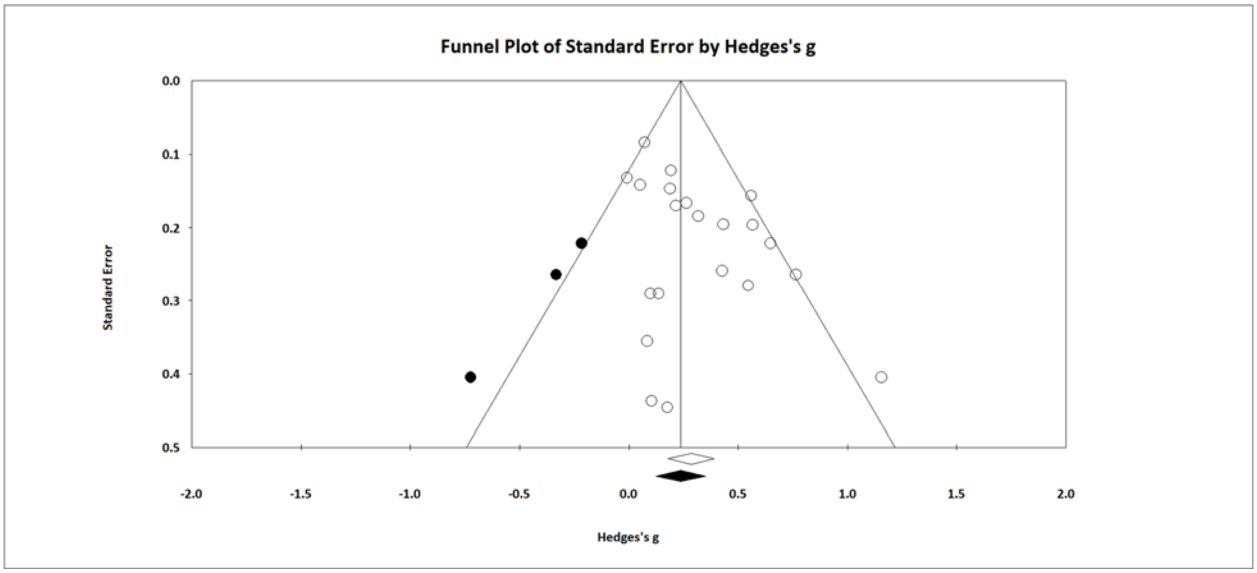

Self-rated ill health (Post-intervention)

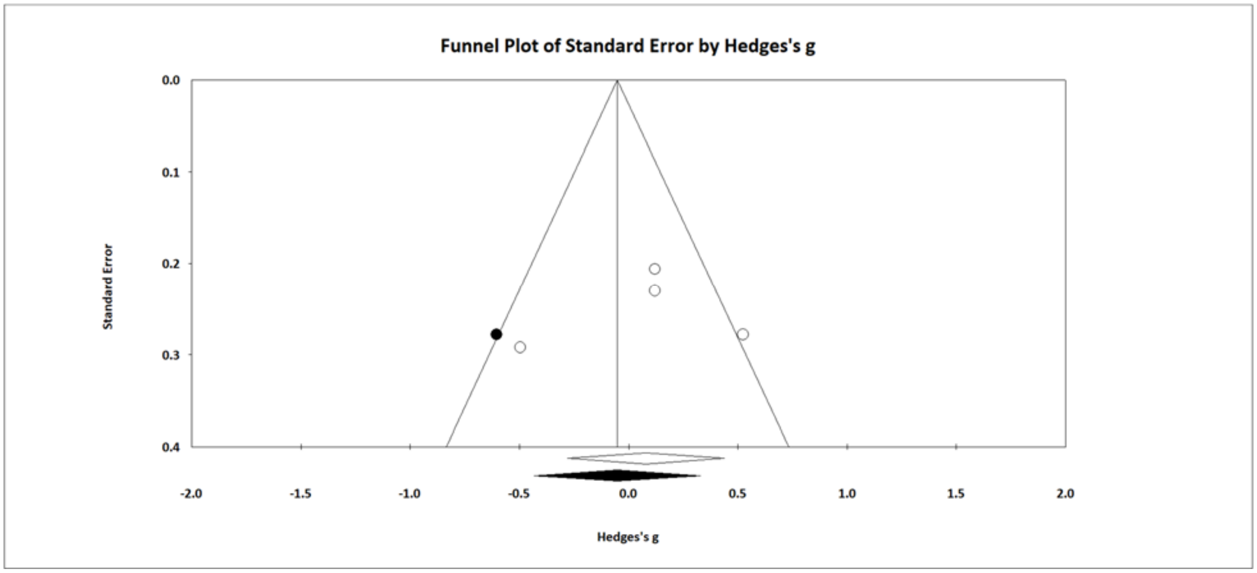

Physical symptoms (First follow-up)

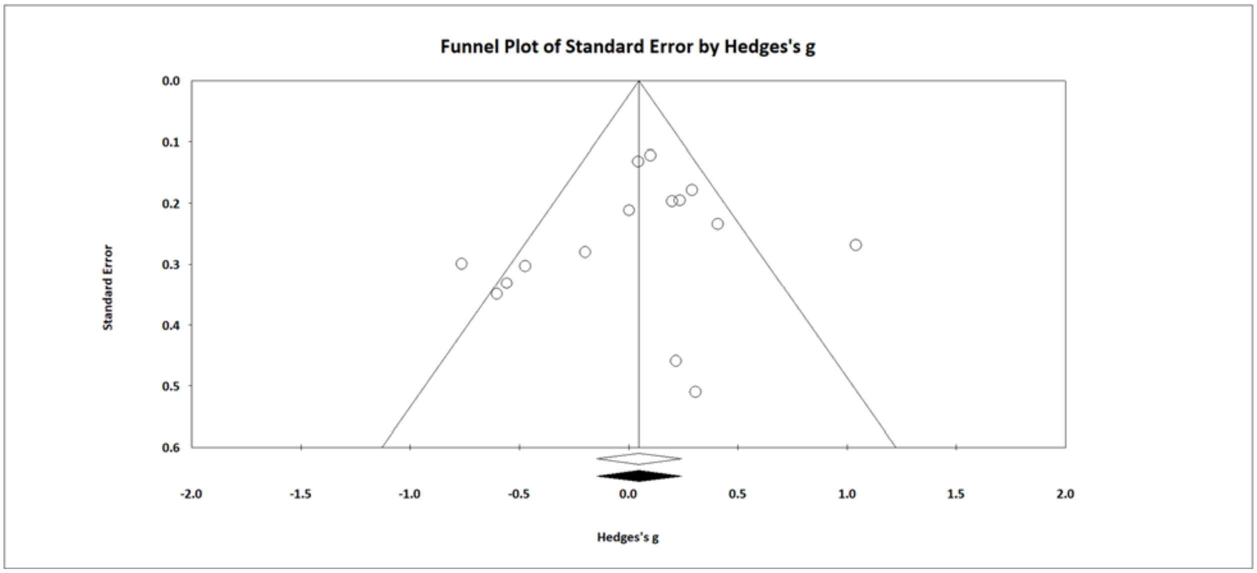

Functional impairment (First follow-up)

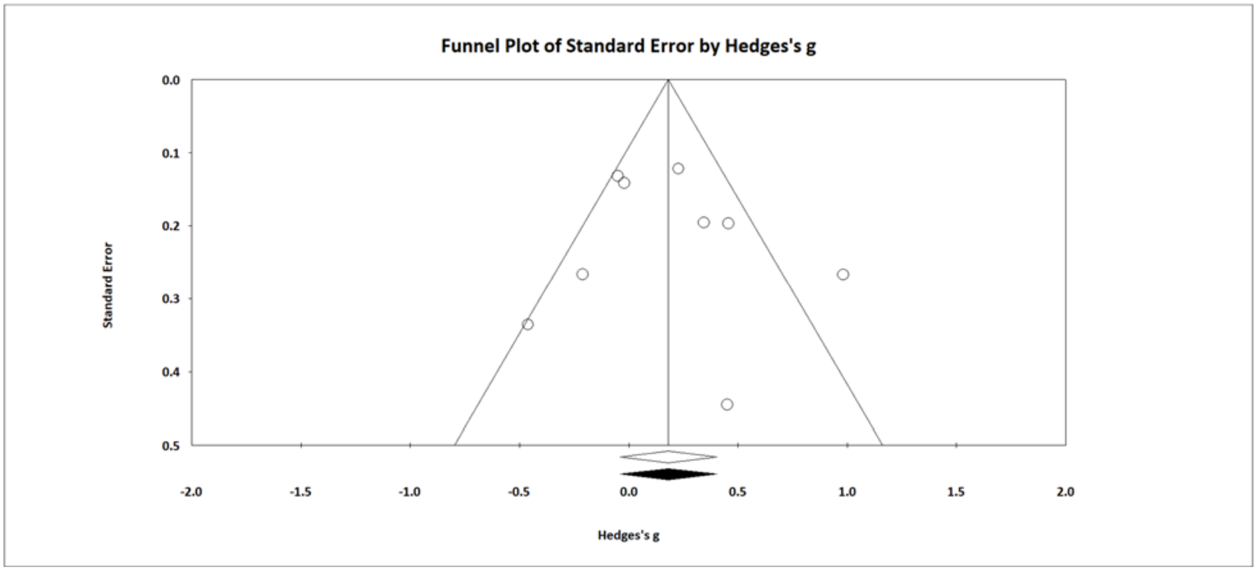

Physical symptoms (Last follow-up)

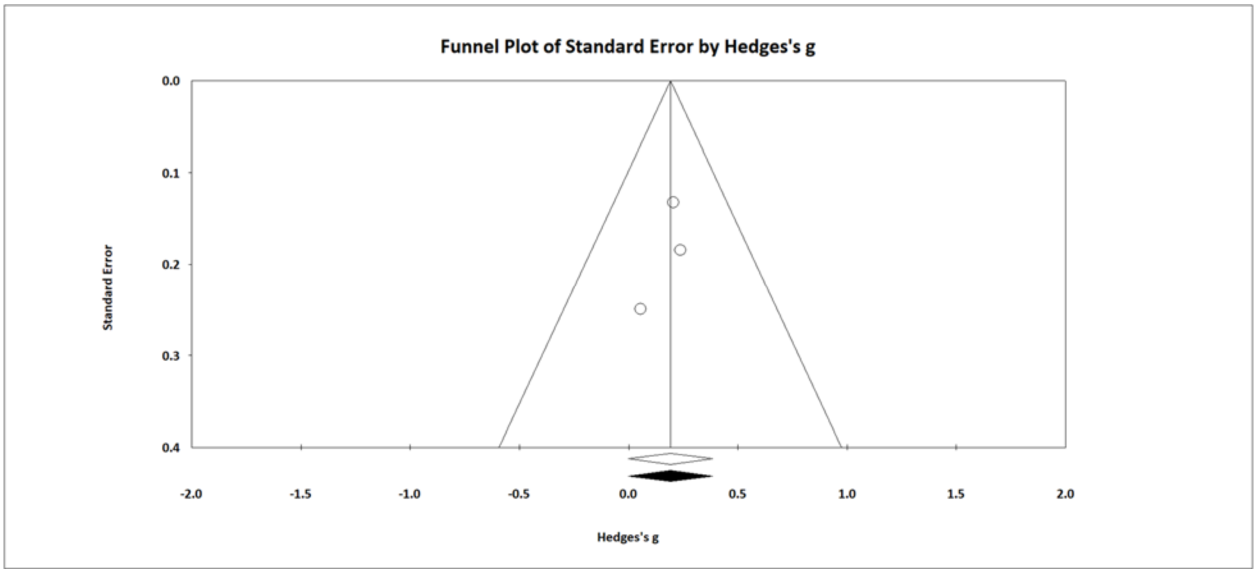

SUPPLEMENTARY FIGURE 3 Regression results between effect sizes of improvements on psychiatric symptoms and physical distress (*n*=58 studies).

a b

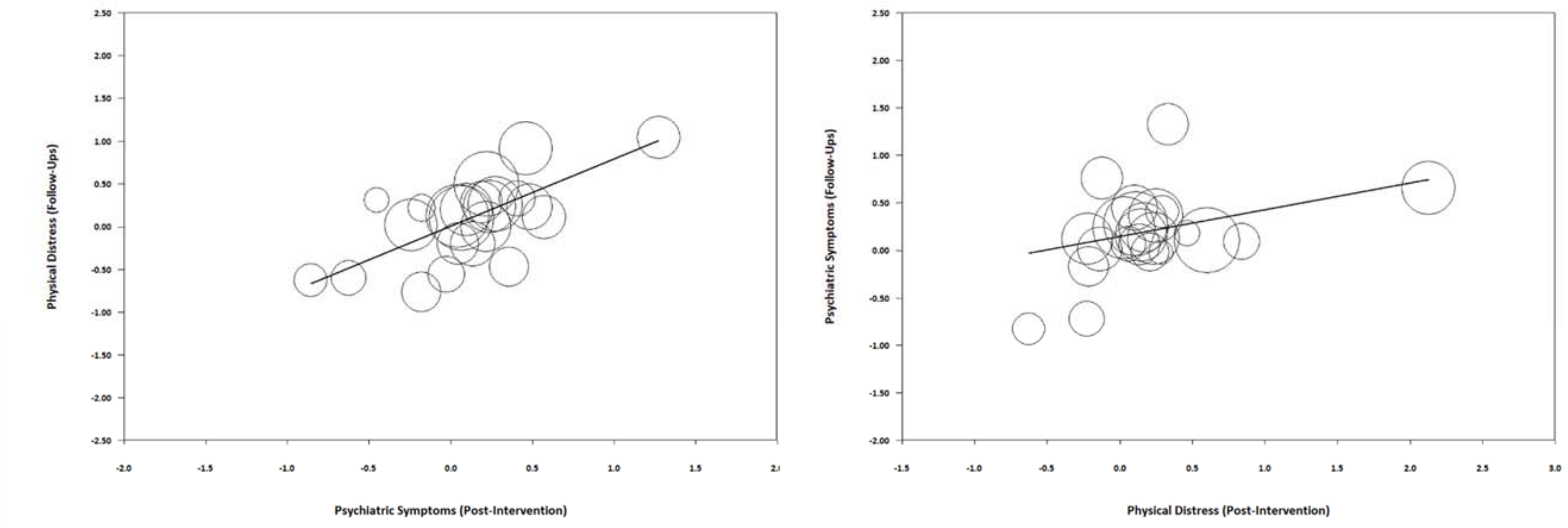

Notes. Left panel a: The regression of averaged effect sizes of physical distress (at follow-ups) on averaged effect sizes of psychiatric symptoms (at post-intervention). Right panel b: The regression of averaged effect sizes of psychiatric symptoms (at follow-ups) on averaged effect sizes of physical distress (at post-intervention).

SUPPLEMENTARY NOTE 1 Amendments to registered protocol.

The registered protocol for this systematic review and meta-analysis is available at PROSPERO (CRD42022265738). Deviations are summarized and explained below:

	Original registration	Deviation	Explanation
Main outcomes	Mental health disorders will be measured by Mini Neuropsychiatric Interview (MINI), anxiety symptoms will be measured by DASS-21, depressive symptoms will be measured by CESD-R, post-traumatic stress symptoms will be measured by PCL-C, dissociative symptoms will be measured by DES-II or equivalent.	Mental health disorders will be measured by Mini Neuropsychiatric Interview (MINI) ***or equivalent***, anxiety symptoms will be measured by DASS-21 ***or equivalent***, depressive symptoms will be measured by CESD-R ***or equivalent***, post-traumatic stress symptoms will be measured by PCL-C ***or equivalent***, dissociative symptoms will be measured by DES-II or equivalent.	During the data extraction process, we were aware of the heterogeneity in scales adopted to measure psychiatric symptoms. Scales adopted were not always listed in our original protocol but could be well-validated scales (e.g., HADS), in which case we still included these studies. A full list of quantitative measures for all outcomes is available in Supplementary Table 5.
Additional outcomes	N/A	***Physical health outcomes will be examined as secondary outcomes,***	We decided to broaden our scope to examine secondary benefits of internet-

		including physical symptoms, functional impairment, self-rated ill health, and objective physiological dysfunction.	based and mobile-based CBT (IM-CBT) on physical health outcomes as well, in order to increase the comprehensibility and rigor of this systematic review and meta-analysis.
Risk of bias (quality) assessment	Two review authors will independently assess the risk of bias in included studies using Newcastle Ottawa Scale. The scale considers three domains including selection, comparability, and outcome.	Two review authors will independently assess the risk of bias in included studies using ***Cochrane risk-of-bias tool for randomized trials (RoB 2)***. The scale considers three domains including selection, comparability, and outcome.	The RoB 2 is an established quality assessment tool for randomized controlled trials (whereas the Newcastle Ottawa Scale for nonrandomized studies).

SUPPLEMENTARY NOTE 2 Detailed search algorithms & criteria for key terminologies.

Four sets of key search terms were devised to combine the following concepts: *chronic diseases*, *cognitive-behavioral therapy*, *psychiatric symptoms*, and *study design*. There was no difference between keyword chains across different databases, except that sets of keywords were entered into different fields (e.g., title or author) depending on the database. The example for PubMed is presented below, in which the keywords for chronic disease and cognitive-behavioral therapy were searched for in the title field, while the keywords for psychiatric symptoms and study design were searched for in all fields:

**Chronic diseases** [Multimorbid* OR Chronic medical condition* OR Chronic illness* OR major chronic disease* OR Chronic physical condition* OR Chronic physical disease* OR Sclerosis* OR Multiple Sclerosis* OR Amyotrophic lateral sclerosis* OR ALS OR Lou Gehrig's Disease* OR Arthritis* OR Osteoarthritis* OR Rheumatoid Arthritis* OR Gout OR Rheumatic Fever OR Carpal Tunnel Syndrome* OR Bursitis* OR Tendinitis* OR Asthma* OR Cancer* OR Carcinoma* OR Malignant tumor* OR Chronic Obstructive Pulmonary Disease* OR COPD OR Chronic bronchitis* OR Emphysema* OR Cystic Fibrosis* OR Diabetes* OR Heart Disease* OR Cardiovascular Disease* OR CVD OR Atherosclerotic* OR Heart failure* OR Valvular heart disease* OR Cardiomyopath* OR Arrhythmias* OR Stroke* OR Hypertension* OR Obesity* OR Obese* OR Oral Health* Or Oral Disease* OR Dental caries* OR Tooth deca* OR Periodontal disease* OR Gum disease* OR Osteoporosis* OR Pain* Or Chronic Pain* Or Complex Regional Pain Syndrome* OR CRPS* OR Reflex Sympathetic Dystrophy Syndrome* OR RSD OR HIV OR HIV infection OR Human immunodeficiency virus* OR AIDS OR Acquired immune deficiency syndrome* OR Hepatitis* OR Hepatitis B OR Hepatitis C OR kidney disease* OR kidney failure*],
**Cognitive-behavioral therapy** [Psychotherapy OR Psychotherap* OR CBT OR Cognitive behavioral therapy OR Behavioral therap* OR Behavioral activation* OR Behavioral strateg* OR Behavioral intervention* OR

Behavi* OR Exposure therap* OR Cognitive therap* OR Cognitive intervention* OR Cognitive reappraisal OR Cognitive restructur* OR Cognitive strateg* OR Cogni* OR Psychoeducation],
**Psychiatric symptoms** [Treatment effectiveness OR Efficac* OR Progress* OR Remission OR Recovery OR Clinically Significant Change OR Good End State OR High End State OR dysthym* OR substance OR drug* Or alcohol* abuse OR dependence OR schizophrenia* personality* disorder* OR anxiety OR anxiety disorder* OR anxiety symptom* OR anxious feeling* OR anxious mood OR depression OR depressive disorder* OR depressive symptom* OR depressed feeling* OR depressed mood* OR post-traumatic stress* OR post-traumatic stress disorder* OR post-traumatic stress symptom* OR post-traumatic stress response* OR traumatic stress OR traumatic stress disorder* OR traumatic symptom* OR traumatic response* OR psychological distress OR psychological symptom* OR psychological dysfunction* OR emotional distress OR psychiatric symptom* OR psychiatric condition* OR mental health*], and
**Study design** [RCT OR randomized controlled* OR randomized clinical* OR randomized placebo-controlled* OR randomized trial*].

We also manually searched the reference lists of relevant previous meta-analyses and of primary articles to locate other potentially eligible studies.

The criteria for chronic diseases, psychiatric symptoms, and physical distress are as follows:

**Chronic diseases** Data on chronic physical conditions were categorized into the following according to the classification system of ICD-11 (World Health Organization, 2022). (1) Certain infectious or parasitic diseases included human immunodeficiency virus (HIV) and hepatitis under ICD-11 Chapter 1. (2) Neoplasms included cancer under ICD-11 Chapter 2. (3) Endocrine, nutritional or metabolic diseases included diabetes mellitus and 5B81 obesity under ICD-11 Chapter 5. (4) Diseases of the nervous system included sclerosis and 8C10.0 Carpal Tunnel Syndrome under ICD-11 Chapter 8. (5) Diseases of the circulatory system included heart disease and hypertension under ICD-11 Chapter 11. (6) Diseases of the respiratory system included CA23 asthma, CA22

chronic obstructive pulmonary disorder (COPD), CA25 cystic fibrosis, CA20.1 chronic bronchitis, and CA21 emphysema under ICD-11 Chapter 12. (7) Diseases of the digestive system included oral and gum diseases, DA08.0 dental caries, and DA0C periodontal disease under ICD-11 Chapter 13. (8) Diseases of the musculoskeletal system or connective tissue included arthritis, tendinitis, FA25 gout, FB83.1 osteoporosis, and FB50 bursitis under ICD-11 Chapter 15. (9) Diseases of the genitourinary system included GB61 chronic kidney disease under ICD-11 Chapter 16. (10) Symptoms, signs or clinical findings, not elsewhere classified included chronic pain under ICD-11 Chapter 21.
**Psychiatric symptoms** Data on mental disorders were categorized into the following according to the classification system of ICD-11 (World Health Organization, 2022). (1) Mood disorders consisted of depressive disorders under ICD-11 Chapter 6. (2) Anxiety or fear-related disorders consisted of anxiety disorders under ICD-11 Chapter 6. (3) Disorders specifically associated with stress hereafter referred to as stress-related disorders, included 6B40 post-traumatic stress disorder under ICD-11 Chapter 6. (4) Disorders of personality, hereafter referred to as personality disorders, included 6D10 personality disorder under ICD-11 Chapter 6. (5) Psychotic disorders included 6A20 schizophrenia under ICD-11 Chapter 6. Note that there were no eligible studies targeting schizophrenia or personality disorders. Note also that PTSD was removed (have to put back PTSD).<br>Apart from the ICD-11 measures, we also identified other general psychological distress measures. It was defined based on the Kessler 10 Psychological Distress Scale (K10), Depression, Anxiety, and Stress Scale (DASS-21), Perceived Stress Scale (PSS10), Perceived Severity of Stress Questionnaire (PSQ), Diabetes Distress Scale, and the Brief Symptom Inventory-18 (BSI-18). More information is available in the section below on “Measurement scales”.
**Physical distress** Based on the findings, we categorized physical distress into 4 categories: physical symptoms (e.g. pain intensity), functional impairment (e.g. gross motor function), self-rated ill health (e.g. general health), and objective physiological dysfunction (e.g. CD4 count). Physical distress related outcomes were secondary measures and thus were not included in the search strategy. More information is available in the section below on “Measurement scales”.

SUPPLEMENTARY NOTE 3 Details on data handling procedures.

Preparation of effect sizes

Hedge's *g* was the preferred effect size metric in this meta-analysis because it was shown to be as accurate as Cohen's *d* for sample sizes >20 and superior to Cohen's *d* for sample sizes <20 (Grissom and Kim, 2005). This is applicable to the current meta-analysis.

To calculate Hedge's *g*, mean and standard deviation (SD) values of targeted mental and physical health outcomes in both the intervention and the control groups at post-treatment and follow-up time points were entered into the Comprehensive Meta-Analysis (CMA) 3.0 software. When the heterogeneity index I^2 was larger than 75%, the adoption of random effects models was justified (Higgins, 2003).

Some articles included more than one treatment arms. When including these studies, we extracted data from each treatment arm into a separate comparison, against the control condition respectively. As suggested by the Cochrane methodology (Higgins et al, 2022), we divided the sample size of the control group in half (if there were two treatment arms) or in thirds (if there were three treatment arms).

Data conversions and handling of missing descriptive statistics for outcomes

For the studies that did not directly provide the SD scores for mental and/or physical health outcomes, but instead provided alternative measurements such as standard error (SE), 95% confidence interval (CI), and/or interquartile range (IQR), specific transformation calculations were performed with reference to the Cochrane handbook.

Statistics available	Handling methods to generate Hedge's g
SE	Standard deviation was obtained by multiplying the standard error of a mean by the square root of the sample size: $SD = SE \times \sqrt{N}$. It is important to note that standard errors were of means calculated from within an intervention group and not standard errors of the difference in means computed between intervention groups.
95% CI	In the case of 95% confidence intervals, standard deviation was obtained by the following formula: $SD = \sqrt{N} \times (upper\ limit - lower\ limit)/2(t\ value)$. T value for 95% confidence interval can be calculated by using formula =tinv(1–0.95,*N*–1) in a cell in a Microsoft Excel spreadsheet. Some articles provided confidence intervals that were not symmetrical about the mean, thereby signaling that the confidence interval may have been calculated on transformed values. In these cases, authors of the articles in question were requested to provide the relevant mean and standard deviation values via email.
IQR	In the case of IQR, the mean and standard deviation was estimated by the formula invented by Luo et al (2018), Wan et al (2014) and their colleagues on the website "Estimating the sample mean and standard deviation from the sample size, median, range and/or interquartile range" (https://www.math.hkbu.edu.hk/~tongt/papers/median2mean.html). After inputting sample size, median, and IQR data, skewness was detected. It was only possible to estimate the mean and standard deviation if the study had a normal distribution. For those with skewed distributions, authors of the articles were requested to provide the mean and standard deviation values via email.

For the articles that provided insufficient information for effect size calculations, authors of the articles were also requested to provide the mean and standard deviation values via email.

Calculating baseline demographic mean and standard deviation values

In order to maintain a standardized and consistent quantitative presentation, all baseline demographic measurements were converted into mean and SD values, given they were not originally presented that way in the eligible studies. When combining mean and SD values of certain variables (e.g., age) from two or more groups, the website "StatTools: Combine Means and SDs Into One Group Program" was used: http://www.obg.cuhk.edu.hk/ResearchSupport/StatTools/CombineMeansSDs_Pgm.php.

In certain cases, range, IQR, and/or median values were provided instead of mean and SD values. To extract the mean and SD values from such available data, this website "Estimating the sample mean and standard deviation from the sample size, median, range and/or interquartile range" was used for transformation: https://www.math.hkbu.edu.hk/~tongt/papers/median2mean.html.

SUPPLEMENTARY NOTE 4 The therapeutic strategy framework for Internet- and Mobile-Based Cognitive Behavioral Therapy (IM-CBT).

In an attempt to classify CBT therapeutic components into categories, five broad categories were defined (Beck, 2020).

One such category is **behavioral modification**, defined as any action-oriented practice targeted at changing behaviors to become more functional and healthier. Strategies in this category, such as behavioral activation and graded exposure, were identified as core components of CBT (Beck, 2020; Craske, 2017; Dryden and Branch, 2011; Josefowitz and Myran, 2021; Wenzel et al, 2016; Wenzel, 2017). Overall, 43 eligible studies incorporated components within the behavioral modification category (Supplementary Table 3), including relaxation skills training, social skills and assertiveness training, behavioral activation, behavioral contracting, positive and negative reinforcement, exposure, living style adjustment, pacing, group discussion, activity scheduling, diary keeping, sleep hygiene/management, behavioral experimentation, physical exercise, physical reconditioning, expressive writing, and community participation.

Another broad category is **cognitive restructuring**, simply defined as a process of assessing and responding to maladaptive thinking. Cognitive restructuring was identified as a key aspect of CBT (Josefowitz and Myran, 2021; Wenzel, 2017; Wenzel et al, 2016) and 30 eligible studies had components within the cognitive restructuring category (Supplementary Table 3).

Another big category is **problem-solving**, defined as the process of identifying problems, generating and evaluating possible solutions, and finally implementing and verifying a chosen solution (Josefowitz and Myran, 2021). Strategies in this category such as identifying values,

setting goals, and relapse prevention, were considered to be salient components of CBT (Beck, 2020; Dryden, 2011; Josefowitz and Myran, 2021; Wenzel, 2017; Wenzel et al, 2016). Overall, 43 eligible studies contained components within the problem-solving category (Supplementary Table 3), including coping skills, relapse prevention, various forms of self-management (e.g., emotional and stress), goal setting, preparing for setbacks and/or high-risk situations, sleep hygiene and management, cue control strategies, locating social support, and identification of core beliefs, stress, and symptoms.

A fourth broad category is **psychoeducation**, defined as involving the transfer of information about CBT, physical health, mental health, the treatment process, and so on, from the therapist to the client, in order to inspire hope in the client (Beck, 2020). Albeit not as broad as the previously mentioned categories, psychoeducation was a heavily reoccurring category in both the eligible studies and existing literature (Beck, 2020; Dryden, 2011). It contained general health advice and introduction to CBT components. 37 eligible studies included components within the psychoeducation category (Supplementary Table 3).

The last category, **mindfulness**, defined as awareness and acknowledgment of one's feelings, thoughts, and bodily sensations in the present moment, without interpretation or judgment (Beck, 2020), is a smaller, yet also very significant third-wave CBT strategy, which has become extremely popular within the CBT movement (Wenzel, 2017) and has been highlighted as an important therapeutic tool (Beck, 2020; Craske, 2017; Wenzel, 2017). Overall, 28 eligible studies included mindfulness as a therapeutic strategy (Supplementary Table 3).

**Category**	**Examples in included articles**
Behavioral modification	Behavioral contracting, pacing, physical exercise, …

Cognitive restructuring	Challenging irrational thoughts, …
Problem-solving	Goal setting, coping skills, emotion management, …
Psychoeducation	General health advice, introduction to CBT, education about mental/physical illnesses, …
Mindfulness	Mindfulness meditation, mindfulness relaxation, self-acceptance/efficacy, biofeedback, self-monitoring, imagery, body scan, …

SUPPLEMENTARY REFERENCES

Eligible articles included in the systematic review and meta-analysis (organized according to alphabetical order) (*44 included studies*)

1. Atema, V. et al. Efficacy of internet-based cognitive behavioral therapy for treatment-induced menopausal symptoms in breast cancer survivors: results of a randomized controlled trial. *J. Clin. Oncol*. **37**, 809–822 (2019).
2. Barroso, J., Madisetti, M. & Mueller, M. A feasibility study to develop and test a cognitive behavioral stress management mobile health application for HIV-related fatigue. *J. Pain Symptom Manag*. **59**, 242–253 (2020).
3. Beatty, L., Koczwara, B. & Wade, T. Evaluating the efficacy of a self-guided web-based CBT intervention for reducing cancer-distress: a randomised controlled trial. *Support. Care Cancer* **24**, 1043–1051 (2016).
4. Buhrman, M., Fältenhag, S., Ström, L. & Andersson, G. Controlled trial of internet-based treatment with telephone support for chronic back pain. *Pain* **111**, 368–377 (2004).
5. Buhrman, M., Nilsson-Ihrfeldt, E., Jannert, M., Ström, L. & Andersson, G. Guided internet-based cognitive behavioural treatment for chronic back pain reduces pain catastrophizing: a randomized controlled trial. *J. Rehabil. Med*. **43**, 500–505 (2011).
6. Buhrman, M. et al. Guided internet-delivered cognitive behavioural therapy for chronic pain patients who have residual symptoms after rehabilitation treatment: randomized controlled trial. *Eur. J. Pain* **17**, 753–765 (2013).

7. Buhrman, M. et al. Individualized guided internet-delivered cognitive-behavior therapy for chronic pain patients with comorbid depression and anxiety. *Clin. J. Pain* **31**, 504–516 (2015).
8. Chambers, S. K. et al. Web-delivered cognitive behavioral therapy for distressed cancer patients: randomized controlled trial. *J. Med. Internet Res*. **20**, e42 (2018).
9. Chiauzzi, E. et al. painACTION-back pain: a self-management website for people with chronic back pain. *Pain Med*. **11**, 1044–1058 (2010).
10. Clarke, J. et al. A web-based cognitive behavior therapy intervention to improve social and occupational functioning in adults with type 2 diabetes (the springboard trial): randomized controlled trial. *J. Med. Internet Res*. **21**, e12246 (2019).
11. Cooper, C. L. et al. Computerised cognitive behavioural therapy for the treatment of depression in people with multiple sclerosis: external pilot trial. *Trials* **12**, 259 (2011).
12. Dear, B. F. et al. The Pain Course: a randomised controlled trial of a clinician-guided internet-delivered cognitive behaviour therapy program for managing chronic pain and emotional well-being. *Pain* **154**, 942–950 (2013).
13. Dear, B. F. et al. The Pain Course: a randomised controlled trial examining an internet-delivered pain management program when provided with different levels of clinician support. *Pain* **156**, 1920–1935 (2015).
14. Doorley, J. D. et al. Feasibility randomized controlled trial of a mind–body activity program for older adults with chronic pain and cognitive decline: the virtual "Active Brains" study. *Gerontologist* **62**, 1082–1094 (2022).
15. Dowd, H. et al. Comparison of an online mindfulness-based cognitive therapy intervention with online pain management psychoeducation. *Clin. J. Pain* **31**, 517–527 (2015).

16. Ferguson, R. J. et al. A randomized trial of videoconference-delivered cognitive behavioral therapy for survivors of breast cancer with self-reported cognitive dysfunction. *Cancer* **122**, 1782–1791 (2016).
17. Ferwerda, M. et al. A tailored-guided internet-based cognitive-behavioral intervention for patients with rheumatoid arthritis as an adjunct to standard rheumatological care: results of a randomized controlled trial. *Pain* **158**, 868–878 (2017).
18. Friesen, L. N. et al. Examination of an internet-delivered cognitive behavioural pain management course for adults with fibromyalgia: a randomized controlled trial. *Pain* **158**, 593–604 (2017).
19. Gasslander, N. et al. Tailored internet-based cognitive behavioral therapy for individuals with chronic pain and comorbid psychological distress: a randomized controlled trial. *Cogn. Behav. Ther*. **51**, 408–434 (2022).
20. Geirhos, A. et al. Feasibility and potential efficacy of a guided internet-and mobile-based CBT for adolescents and young adults with chronic medical conditions and comorbid depression or anxiety symptoms (youthCOACHCD): a randomized controlled pilot trial. *BMC Pediatr*. **22**, 69 (2022).
21. Glozier, N. et al. Internet-delivered cognitive behavioural therapy for adults with mild to moderate depression and high cardiovascular disease risks: a randomised attention-controlled trial. *PLoS One* **8**, e59139 (2013).
22. Ham, K. et al. Preliminary results from a randomized controlled study for an app-based cognitive behavioral therapy program for depression and anxiety in cancer patients. *Front. Psychol*. **10**, 1592 (2019).

23. Hummel, S. B. et al. Efficacy of internet-based cognitive behavioral therapy in improving sexual functioning of breast cancer survivors: results of a randomized controlled trial. *J. Clin. Oncol*. **35**, 1328–1340 (2017).

24. Johansson, P. et al. An internet-based cognitive behavioral therapy program adapted to patients with cardiovascular disease and depression: randomized controlled trial. *JMIR Ment. Health* **6**, e14648 (2019).

25. Lundgren, J. G. et al. The effect of guided web-based cognitive behavioral therapy on patients with depressive symptoms and heart failure: a pilot randomized controlled trial. *J. Med. Internet Res*. **18**, e5556 (2016).

26. Migliorini, C., Sinclair, A., Brown, D., Tonge, B. & New, P. A randomised control trial of an internet-based cognitive behaviour treatment for mood disorder in adults with chronic spinal cord injury. *Spinal Cord* **54**, 695–701 (2016).

27. Mourad, G. et al. Guided Internet-delivered cognitive behavioural therapy in patients with non-cardiac chest pain–a pilot randomized controlled study. *Trials* **17**, 352 (2016).

28. Murphy, M. J. et al. Randomised controlled trial of internet-delivered cognitive behaviour therapy for clinical depression and/or anxiety in cancer survivors (iCanADAPT early). *Psycho-Oncol*. **29**, 76–85 (2020).

29. Newby, J. et al. Web-based cognitive behavior therapy for depression in people with diabetes mellitus: a randomized controlled trial. *J. Med. Internet Res*. **19**, e7274 (2017).

30. O'moore, K. A. et al. Internet cognitive–behavioral therapy for depression in older adults with knee osteoarthritis: a randomized controlled trial. *Arthritis Care Res*. **70**, 61–70 (2018).

31. Palermo, T. M., Wilson, A. C., Peters, M., Lewandowski, A. & Somhegyi, H. Randomized controlled trial of an internet-delivered family cognitive–behavioral therapy intervention for children and adolescents with chronic pain. *Pain* **146**, 205–213 (2009).

32. Palermo, T. M. et al. Internet-delivered cognitive-behavioral treatment for adolescents with chronic pain and their parents: a randomized controlled multicenter trial. *Pain* **157**, 174–185 (2016).

33. Peters, M. L. et al. Happy despite pain: a randomized controlled trial of an 8-week internet-delivered positive psychology intervention for enhancing well-being in patients with chronic pain. *Clin. J. Pain* **33**, 962–975 (2017).

34. Shigaki, C. L. et al. RAHelp: an online intervention for individuals with rheumatoid arthritis. *Arthritis Care Res*. **65**, 1573–1581 (2013).

35. Simblett, S. K. et al. Computerized cognitive behavioral therapy to treat emotional distress after stroke: a feasibility randomized controlled trial. *JMIR Ment. Health* **4**, e6022 (2017).

36. Stinson, J. N. et al. An internet-based self-management program with telephone support for adolescents with arthritis: a pilot randomized controlled trial. *J. Rheumatol*. **37**, 1944–1952 (2010).

37. Taguchi, K. et al. Clinical effectiveness and cost-effectiveness of videoconference-based integrated cognitive behavioral therapy for chronic pain: randomized controlled trial. *J. Med. Internet Res*. **23**, e30690 (2021).

38. Thesen, T. et al. Effectiveness of internet-based cognitive behavioral therapy with telephone support for noncardiac chest pain: randomized controlled trial. *J. Med. Internet Res*. **24**, e33631 (2022).

39. Trautmann, E. & Kröner-Herwig, B. A randomized controlled trial of internet-based self-help training for recurrent headache in childhood and adolescence. *Behav. Res. Ther*. **48**, 28–37 (2010).

40. Trudeau, K. J. et al. A randomized controlled trial of an online self-management program for adults with arthritis pain. *J. Behav. Med*. **38**, 483–496 (2015).

41. Westas, M., Lundgren, J., Andersson, G., Mourad, G. & Johansson, P. Effects of internet-delivered cognitive behavioural therapy adapted for patients with cardiovascular disease and depression: a long-term follow-up of a randomized controlled trial at 6 and 12 months posttreatment. *Eur. J. Cardiovasc. Nurs*. **21**, 559–567 (2022).

42. Wiklund, T. et al. Internet-delivered cognitive behavioral therapy for insomnia comorbid with chronic pain: randomized controlled trial. *J. Med. Internet Res*. **24**, e29258 (2022).

43. Williams, D. A. et al. Internet-enhanced management of fibromyalgia: a randomized controlled trial. *Pain* **151**, 694–702 (2010).

44. Wilson, M. et al. Engaging adults with chronic disease in online depressive symptom self-management. *West. J. Nurs. Res*. **40**, 834–853 (2018).

Eligible articles included in the systematic review and meta-analysis (organized according to alphabetical order) (*14 studies in appendix only*)

1. Andreae, S. J., Andreae, L. J., Richman, J. S., Cherrington, A. L. & Safford, M. M. Peer-delivered cognitive behavioral therapy-based intervention reduced depression and stress in community dwelling adults with diabetes and chronic pain: a cluster randomized trial. *Ann. Behav. Med*. **55**, 970–980 (2021).

2. Carmody, T. P. et al. Telephone-delivered cognitive–behavioral therapy for pain management among older military veterans: a randomized trial. *Psychol. Serv*. **10**, 265–275 (2013).

3. Casault, L., Savard, J., Ivers, H. & Savard, M. H. A randomized-controlled trial of an early minimal cognitive-behavioural therapy for insomnia comorbid with cancer. *Behav. Res. Ther*. **67**, 45–54 (2015).

4. Doyle, C. et al. The impact of telephone-delivered cognitive behaviour therapy and befriending on mood disorders in people with chronic obstructive pulmonary disease: a randomized controlled trial. *Br. J. Health Psychol*. **22**, 542–556 (2017).

5. Eller, L. S. Effects of two cognitive-behavioral interventions on immunity and symptoms in persons with HIV. *Ann. Behav. Med*. **17**, 339–348 (1995).

6. Howard, C. & Dupont, S. 'The COPD breathlessness manual': a randomised controlled trial to test a cognitive-behavioural manual versus information booklets on health service use, mood and health status, in patients with chronic obstructive pulmonary disease. *npj Prim. Care Respir. Med*. **24**, 14076 (2014).

7. Kelleher, S. A. et al. Feasibility, engagement, and acceptability of a behavioral pain management intervention for colorectal cancer survivors with pain and psychological distress: data from a pilot randomized controlled trial. *Support. Care Cancer* **29**, 5361–5369 (2021).

8. Kraaij, V. et al. Effects of a cognitive behavioral self-help program and a computerized structured writing intervention on depressed mood for HIV-infected people: a pilot randomized controlled trial. *Patient Educ. Couns*. **80**, 200–204 (2010).

9. McAndrew, L. M., Greenberg, L. M., Ciccone, D. S., Helmer, D. A. & Chandler, H. K. Telephone-based versus in-person delivery of cognitive behavioral treatment for veterans with chronic multisymptom illness: a controlled, randomized trial. *Mil. Behav. Health*. **6**, 56–65 (2018).
10. McCurry, S. M. et al. Effect of telephone cognitive behavioral therapy for insomnia in older adults with osteoarthritis pain: a randomized clinical trial. *JAMA Intern. Med*. **181**, 530–538 (2021).
11. Mohr, D. C. et al. Telephone-administered cognitive–behavioral therapy for the treatment of depressive symptoms in multiple sclerosis. *J. Consult. Clin. Psychol*. **68**, 356–361 (2000).
12. Rawlings, G. H., Beail, N., Armstrong, I. & Thompson, A. R. Self-help cognitive behavioural therapy for anxiety in pulmonary hypertension: pilot randomised controlled trial. *ERJ Open Res*. **8**, 00526-2021 (2022).
13. Savard, J., Ivers, H., Savard, M. H. & Morin, C. M. Is a video-based cognitive behavioral therapy for insomnia as efficacious as a professionally administered treatment in breast cancer? results of a randomized controlled trial. *Sleep* **37**, 1305–1314 (2014).
14. Stefanopoulou, E., Yousaf, O., Grunfeld, E. A. & Hunter, M. S. A randomised controlled trial of a brief cognitive behavioural intervention for men who have hot flushes following prostate cancer treatment (MANCAN). *Psycho-Oncol*. **24**, 1159–1166 (2015).

Other references cited within this document (organized according to alphabetical order)

1. Beck, J. S. *Cognitive Behavior Therapy: Basics and Beyond* (Guilford Publications, 2020).
2. Craske, M. G. *Cognitive-Behavioral Therapy* (2nd ed.) (American Psychological Association, 2017).

3. Dryden, W. & Branch, R. *The CBT Handbook* (Sage, 2011).
4. Grissom, R. J. & Kim, J. J. *Effect Sizes for Research: A Broad Practical Approach* (Lawrence Erlbaum Associates Publishers, 2005).
5. Higgins, J. P.T., Eldridge, S. & Li, T. Chapter 23: Including variants on randomized trials. In: Higgins, J. P. T., Thomas, J., Chandler, J., Cumpston, M., Li, T., Page, M. J. & Welch, V. A. (eds.) *Cochrane Handbook for Systematic Reviews of Interventions* (Version 6.3). www.training.cochrane.org/handbook (2022).
6. Higgins, J. P., Thompson, S. G., Deeks, J. J. & Altman, D. G. Measuring inconsistency in meta-analyses. *BMJ* **327**, 557–560 (2003).
7. Josefowitz, N. & Myran, D. *CBT Made Simple: A Clinician's Guide to Practicing Cognitive Behavioral Therapy* (New Harbinger Publications, 2021).
8. Karyotaki, E. et al. Internet-based cognitive behavioral therapy for depression: a systematic review and individual patient data network meta-analysis. *JAMA Psychiatry* **78**, 361–371 (2021).
9. Luo, D., Wan, X., Liu, J. & Tong, T. Optimally estimating the sample mean from the sample size, median, mid-range, and/or mid-quartile range. *Stat. Methods Med. Res*. **27**, 1785–1805 (2018).
10. Schulz, K. F. & Grimes, D. A. Sample size slippages in randomised trials: exclusions and the lost and wayward. *Lancet* **359**, 781–785 (2002).
11. van Beugen, S. et al. Internet-based cognitive behavioral therapy for patients with chronic somatic conditions: a meta-analytic review. *J. Med. Internet Res*. **16**, e2777 (2014).

12. Wan, X., Wang, W., Liu, J. & Tong, T. Estimating the sample mean and standard deviation from the sample size, median, range and/or interquartile range. *BMC Med. Res. Methodol.* **14**, 135 (2014).

13. Wenzel, A. Basic strategies of cognitive behavioral therapy. *Psychiatric Clinics* **40**, 597–609 (2017).

14. Wenzel, A., Dobson, K. S. & Hays, P. A. *Cognitive Behavioral Therapy Techniques and Strategies* (American Psychological Association, 2016).
